# Azadiphosphaindane‐1,3‐diyls: A Class of Resonance‐Stabilized Biradicals

**DOI:** 10.1002/anie.202011886

**Published:** 2020-11-19

**Authors:** Jonas Bresien, Dirk Michalik, Axel Schulz, Alexander Villinger, Edgar Zander

**Affiliations:** ^1^ Institut für Chemie Universität Rostock Albert-Einstein-Straße 3a 18059 Rostock Germany; ^2^ Leibniz-Institut für Katalyse e.V. Albert-Einstein-Straße 29a 18059 Rostock Germany

**Keywords:** aromaticity, biradicals, heterocycles, molecule activation, phosphorus

## Abstract

Conversion of 1,2‐bis(dichlorophosphino)benzene with sterically demanding primary amines led to the formation of 1,3‐dichloro‐2‐aza‐1,3‐diphosphaindanes of the type C_6_H_4_(μ‐PCl)_2_N‐R. Reduction yielded the corresponding 2‐aza‐1,3‐diphosphaindane‐1,3‐diyls (**1**), which can be described as phosphorus‐centered singlet biradical(oid)s. Their stability depends on the size of the substituent R: While derivatives with R=Dmp (2,6‐dimethylphenyl) or Ter (2,6‐dimesitylphenyl) underwent oligomerization, the derivative with very bulky R=^tBu^Bhp (2,6‐bis(benzhydryl)‐4‐tert‐butylphenyl) was stable with respect to oligomerization in its monomeric form. Oligomerization involved activation of the fused benzene ring by a second equivalent of the monomeric biradical and can be regarded as formal [2+2] (poly)addition reaction. Calculations indicate that the biradical character in **1** is comparable with literature‐known P‐centered biradicals. Ring‐current calculations show aromaticity within the entire ring system of **1**.

## Graphical abstract

Only one very bulky substituent R is required to stabilize the first stable heteroindanediyls (see picture), which represent a group of resonance‐stabilized phosphorus‐centered biradicals. Different types of oligomers formed by self‐activation of the aromatic backbone were observed for smaller substituents.

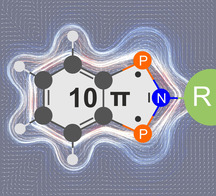

Singlet biradical(oid)s are molecules with two electrons in two nearly degenerate orbitals.[^1^, ^2^, ^3^, ^4^] Although their spin density is zero at every point in space, biradicals can show extraordinary reactivity that ranges between monoradicals and closed‐shell molecules.^[5]^ Starting with pioneering work by Niecke et al., who synthesized the 1,3‐diphosphacyclobutane‐2,4‐diyl [Mes*P(μ‐CCl)]_2_ in 1995,^[6]^ stable main‐group‐centered biradicals came into focus of many further investigations.[^7^, ^8^, ^9^, ^10^, ^11^] For example, our group performed comprehensive research on the phosphorus‐centered biradical [P(μ‐NTer)]_2_ (**A**), which was synthesized from a chlorinated precursor by reduction with elemental magnesium (Scheme 1).^[12]^ Biradical **A** is highly reactive towards polar and non‐polar single, double, and triple bonds (e.g., H_2_, S_8_, O_2_, ketones, alkenes, alkynes, nitriles), typically resulting in addition products with tri‐ or penta‐valent phosphorus atoms.^[13]^

**Scheme 1:**
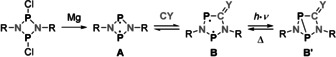
Synthesis of [P(μ‐NR)]_2_ with R=Ter^[12]^ (**A**) and ring expansion with CY (Y=O^[14]^ or NR′^[15]^) to biradicals of type **B** (heterocyclopentanediyls). **B** can be photo‐isomerized to the housane‐type isomer **B′**.

In contrast, CO^[14]^ or isonitriles^[15]^ insert into the four‐membered ring system, leading to stable five‐membered cyclic biradicals of type **B** (Scheme 1). Other pnictogen‐based, five‐membered cyclic biradicals (heterocyclopentane‐1,3‐diyls) are synthesized using the same approach, with varying substituents or pnictogen atoms.[^15^, ^16^] Yet, the activation chemistry of biradicals **B** is often limited by the reversibility of the CO or isonitrile insertion, as the utilization of biradicals **A** and **B** often leads to the same activation products (Scheme 2).[^14^, ^15^, ^17^, ^18^] Still, biradicals of the type **B** are worthwhile target molecules, as they can be reversibly photo‐isomerized to a closed‐shell housane‐type isomer **B′** with a transannular P−P bond, leading to potential applications as molecular switches (Scheme 1).[^19^, ^20^]

**Scheme 2:**
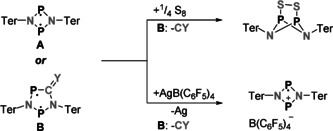
Due to elimination of CY, reactions with biradicals **B** often lead to the same reaction products as found for biradical **A**.[^14^, ^15^, ^17^, ^18^]

To overcome the instability of **B** with respect to elimination of the CY moiety, we chose to investigate structurally related benzo‐fused cyclopentane‐1,3‐diyls (i.e., heteroindanediyls **1**, Scheme 3), which might also provide aromatic stabilization of the biradical moiety.

**Scheme 3:**
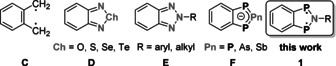
*Ortho*‐quinodimethane^[21]^ (**C**) and heteroindane derivatives **D**,[^22^, ^23^, ^24^, ^25^, ^26^, ^27^] **E**,^[28]^ and **F**[^29^, ^30^] with pnictogen atoms in 1,3‐position.

An example of closely related, delocalized biradicals are *ortho*‐quinodimethanes (**C**, Scheme 3), which are known as reactive intermediates in organic synthesis.^[21]^ Furthermore, isoelectronic heteroindane derivatives with Group‐15 elements in 1,3‐position were reported, such as a variety of stable benzo‐2‐chalco‐1,3‐diazoles[^22^, ^23^, ^24^, ^25^, ^26^, ^27^] (**D**), 2‐substituted benzotriazoles^[28]^ (**E**), and 2‐pnicta‐1,3‐diphosphaindenyl anions[^29^, ^30^] (**F**). The biradical character of these compounds (**D**–**F**) has not been evaluated yet.

As no reports about target compound **1** were found in the literature, we opted to synthesize different derivatives with differently sized subtituents (Dmp, Ter, and ^*t*Bu^Bhp) in order to investigate the kinetic stability of **1** towards di‐ or oligomerization (for descriptors of steric demand, see Supporting Information, p. S44 ff).[^17^, ^31^, ^32^] In a first step, a suitable precursor for biradical **1** was synthesized: By analogy with the synthesis of **A** (Scheme 1), chlorinated azadiphosphaindanes (**2**) were prepared by reaction of primary amines with 1,2‐bis(dichlorophosphino)benzene[^33^, ^34^] (Scheme 4).^[66]^ For all substituents (Dmp, Ter, ^*t*Bu^Bhp), the *cis* isomer of **2** (*cis* with respect to the Cl atoms, Figures S1, S2) was obtained (^31^P NMR: *δ*=147–149 ppm). Only in the case of **2Dmp** the *trans* isomer was observed as side product (^31^P NMR: *δ*=171 ppm). DFT calculations showed that the *cis* isomers of **2** are energetically favored for all substituents (Δ*G*_r_°=9–20 kJ mol^−1^, cf. SI).

**Scheme 4:**
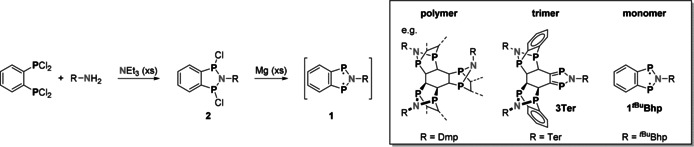
Synthesis of differently substituted 2‐aza‐1,3‐diphosphaindane‐1,3‐diyls **1**. Their stability depends on the sterical demand of the substituent R, as depicted on the right.

The synthesis of heteroindanediyls **1** (R=Dmp, Ter, ^*t*Bu^Bhp) was achieved by reduction of **2** with elemental Mg analogous to the synthesis of **A**.[^12^, ^19^] During the reaction, the colorless solutions turned orange, indicating the formation of the desired product (Scheme 4). This could be confirmed by ^31^P NMR spectroscopy; all heteroindanediyls **1** (R=Dmp, Ter, ^*t*Bu^Bhp) could be identified by a characteristic singlet resonance (*δ*=280–285 ppm), which compares well with related 1,2,5‐azadiphospholes (*t*BuC)_2_(μ‐P)_2_N*t*Bu (*δ*=286 ppm)[^35^, ^36^] or biradicals **A** (276 ppm)^[19]^ and **B** (221, 258 ppm).^[19]^ However, depending on the steric demand of R, different follow‐up reactions were observed (Scheme 4). Biradical **1Dmp** fully converted to an insoluble red polymer within one day, as evidenced by ^31^P NMR spectroscopy (Figure S8). The polymer was isolated and analyzed by elemental analysis and vibrational spectroscopy (cf. SI, p. S27 ff).

In the case of **1Ter**, a selective trimerization to **3Ter** was observed. **3Ter** was formed via activation of the fused benzene ring of **1Ter** by two further equivalents of the monomeric biradical (time‐dependent ^31^P NMR spectra cf. Figure S10). This self‐activation process can be regarded as formal [2+2] addition reaction. The structural motif of **3Ter** is yet unknown and represents the first example of a six‐membered carbon cycle substituted by six P atoms.

In the ^31^P{^1^H} NMR spectrum, **3Ter** displays an AA′BB′XX′ spin system (Figure 1) due to its *C*_2_ symmetry in solution. The shift of the P_X_ nuclei (287 ppm) is comparable to the resonance of monomeric **1**. The three‐valent P_A_ (*δ*=82 ppm) and P_B_ (*δ*=89 ppm) nuclei show a significant upfield shift, with well resolved *J*_AB_ (−31 Hz), *J*_BX_ (98 Hz), and *J*_XX′_ (−18 Hz) coupling constants. The absolute values of all other coupling constants are significantly smaller than 5 Hz, but essential for the coupling pattern. The experimental data agree well with calculated NMR shifts and coupling constants (cf. Table S3).

**Figure 1:**
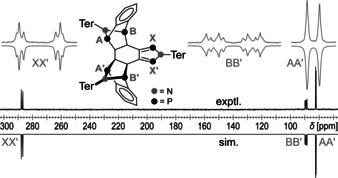
Experimental and simulated^[37]^ ^31^P{^1^H} NMR spectrum of **3Ter**.

Crystallization of **3Ter** from benzene yielded colorless crystals. The solid‐state structure was determined by single‐crystal X‐ray diffraction (Figure 2). **3Ter** crystallized in the triclinic space group *P* $\bar{1}$ with two molecules **3Ter** and eight highly disordered benzene molecules per unit cell. The central condensed ring system is nearly planar (∡(N1‐P2‐P1‐C6)=−179.7(3)°, ∡P1‐C6‐C1‐C2)=175.4(3)° and (∡(C1‐C2‐C5‐C4)=178.6(4)°). The P1−C6 and P2−C1 bond lengths (1.698(3) and 1.719(3) Å) are almost identical and lie in the range of the sum of the covalent radii of a P=C double bond (∑*r*_cov_(P−C)=1.86 Å, ∑*r*_cov_(P=C)=1.69 Å),^[38]^ while the C1−C6 bond (1.397(5) Å) is slightly longer than the value expected for a C=C double bond (∑*r*_cov_(C−C)=1.50 Å, ∑*r*_cov_(C=C)=1.34 Å).^[38]^ These structural parameters indicate a dominant diene structure with localized P=C double bonds (see computations below). The transannular P1–P2 distance is 2.921(3) Å and therefore significantly longer than a P−P single bond (∑*r*_cov_(P−P)=2.22 Å).^[38]^

**Figure 2:**
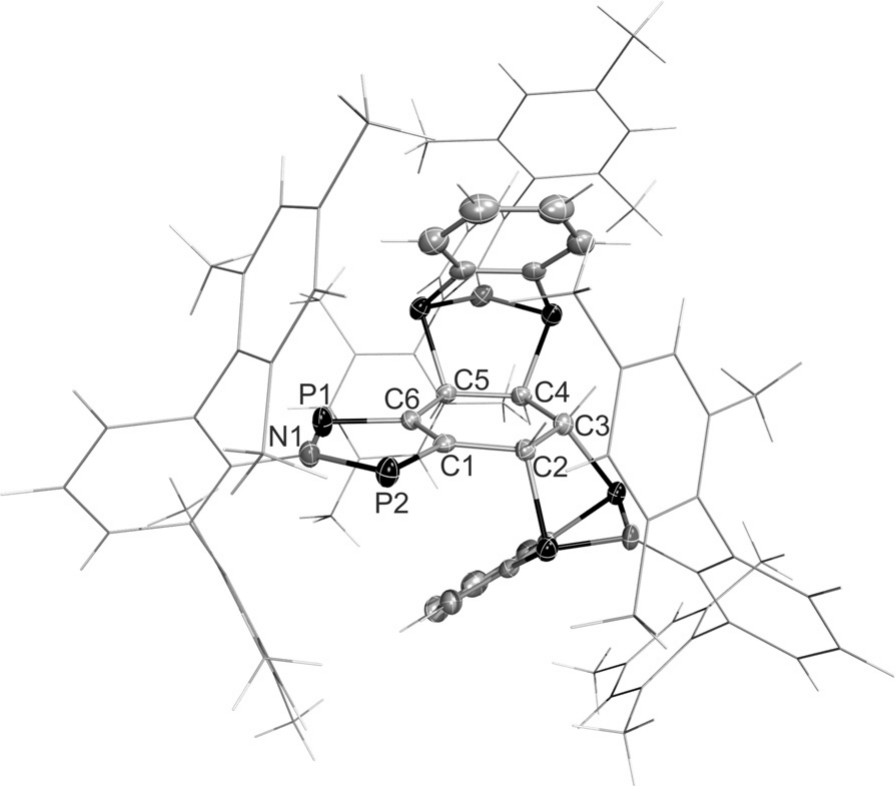
Molecular structure of **3Ter**.^[66]^ Ellipsoids are set at 50 % probability (123 K). Selected bond lengths [Å] and dihedral angles [°]:C1–C2=1.509(5), C1–C6=1.397(5), C2–C3=1.559(5), C3–C4= 1.554(5), C4–C5=1.558(5), C5–C6=1.505(5), N1–P1=1.698(3), N1–P2=1.698(3), P1–C6=1.698(3), P2–C1=1.719(3), P1–P2= 2.921(3); C1‐C2‐C5‐C4=178.6(4), N1‐P2‐P1‐C6=−179.7(3), P1‐C6‐C1‐C2=175.4(3).

**1** ^***t*****Bu**^ **Bhp**, the most sterically demanding derivative, was stable in benzene solution for several weeks, as verified by NMR spectroscopy. **1** ^***t*****Bu**^ **Bhp** is intensely yellow and shows absorption maxima at 407 and 424 nm in the UV/Vis spectrum (benzene solution). According to time‐dependent density functional theory (TD‐DFT) calculations, the main absorption at 424 nm can be attributed to the formal HOMO→LUMO transition (*λ*_calcd_=470 nm, PBE‐D3/def2‐TZVP).

**1** ^***t*****Bu**^ **Bhp** could be crystallized from toluene and was examined by single‐crystal X‐ray diffraction (Figure 3). It crystallized in the monoclinic space group *P*2_1_/*n* with four molecules per unit cell. Similarly to **3Ter**, the heteroindanediyl moiety is planar within the margin of error (∡(N1‐P1‐P2‐C37)=−179.4(2)°, ∡P1‐C37‐C42‐C41)=−177.9(2)°, ∡(C42‐C41‐C38‐C39)=179.3(3)°). Yet, both P−C bonds (1.742(2) Å) are significantly elongated compared to **3Ter**, indicating a reduced P−C double bond character, and thus a delocalized π‐bonding system (see computations below). The transannular P–P distance (2.9574(7) Å) is similar to **3Ter** and type **B** biradicals (Y=O: 2.961 Å^[14]^;Y=NDmp: 2.944 Å^[19]^).

**Figure 3:**
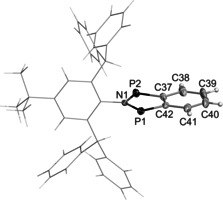
Molecular structure of **1** ^***t*****Bu**^ **Bhp**.^[66]^ Ellipsoids are set at 50 % probability (123 K). Selected bond lengths [Å] and dihedral angles [°]: P1–N1=1.696(2), P1–C42=1.742(2), P2–N1=1.692(2), P2–C37= 1.742(2), C37–C38=1.425(3), C37–C42=1.428(3), C38–C39= 1.365(3), C39–C40=1.406(3), C40–C41=1.357(3), C41–C42= 1.426(2); C42‐C41‐C38‐C39=179.3(3), N1‐P1‐P2‐C37=−179.4(2), P1‐C37‐C42‐C41=−177.9(2).

Theoretical investigations[^41^, ^42^, ^43^, ^44^, ^45^, ^46^, ^47^] were carried out to quantify the biradical character of compounds **1** ^***t*****Bu**^ **Bhp** and **3Ter**. CASSCF[^48^, ^49^, ^50^, ^51^, ^52^, ^53^, ^54^, ^55^, ^56^] calculations were performed to obtain a correct description of the multireference character (cf. SI, p. S41 ff). The biradical character was quantified by the LUMO occupation number and *β* scale (defined as *β*=2 c22 /(c21 +c22 ) by Xantheas et al.).^[3]^ First, simple CAS(2,2) calculations were performed, which ignore any dynamic correlation within the π‐bonding system. In this simple picture, LUMO occupancy and *β* are identical by definition. The biradical character of **1** ^***t*****Bu**^ **Bhp** amounts to 18 %, which is slightly lower in comparison with other biradicals such as **A** and **B** (Table 1). In contrast, the biradical character of **3Ter** (12 %) is significantly smaller, so it is better described as a diene. This is in accord with other literature reports.^[20]^

**Table 1:** LUMO occupancy and biradical character *β* ^[3]^ for selected compounds. Further descriptors can be found in Table S16.[^39^, ^40^]

		**A**	**B^[a]^**	**1** ^***t*****Bu**^ **Bhp**	**3Ter**
**CAS(2,2)**	**LUMO occ**.	0.28	0.28	0.18	0.12
	***β***	0.28	0.28	0.18	0.12
					
**full π** **CAS^[b]^**	**LUMO occ**.	0.27	0.28	0.21	0.12
	***β***	0.26	0.27	0.14	0.11

[a] with Y=NDmp. [b] All π‐type electrons of the central ring fragment were included in the active space (**A**: CAS(6,4), **B**: CAS(8,6), **1** ^***t*****Bu**^ **Bhp**: CAS(10,9), **3Ter**: CAS(6,4)).

Secondly, CAS calculations including all π‐type orbitals of the main ring fragment were performed, thus including non‐dynamic and dynamic correlation within the π‐bonding system (Figure 4, Figures S15–S18). In case of **1** ^***t*****Bu**^ **Bhp**, this procedure led to significantly different values for LUMO occupancy and *β*, while these values hardly differed in case of **A**, **B**, and **3Ter** (Table 1). As *β* is based on only two coefficients of the CAS wave function, whereas the LUMO occupancy reflects a sum over many determinants, large deviations indicate a strongly correlated wave function. Nonetheless, considering that all coefficients apart from *c*_1_ and *c*_2_ individually contributed about 1 % or less to the CAS wave function, the difference between LUMO occupancy and *β* is primarily attributed to dynamic correlation.

**Figure 4:**
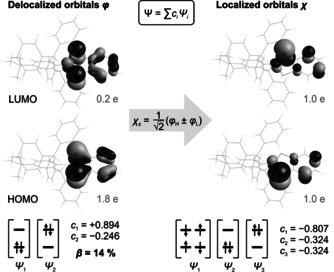
Frontier orbitals of **1** ^***t*****Bu**^ **Bhp** (CAS(10,9)/def2‐TZVP//PBE‐D3/def2‐TZVP). Only the main contributions to the wave function are given. For an illustration of all molecular orbitals within the active space see Figure S17.

In the localized orbital picture, it is apparent that biradical **1** ^***t*****Bu**^ **Bhp** also possesses some zwitterionic character (approx. 80 % covalent, 20 % ionic), as evidenced by contributions of determinants *Ψ*_2_ and *Ψ*_3_ (Figure 4, right). The “biradical electrons” are mainly localized at the P atoms, but also somewhat delocalized across the fused benzene ring. This is, of course, a unique feature of the benzo‐fused ring system in **1** ^***t*****Bu**^ **Bhp** compared to biradicals **A** or **B** (Scheme 1).

All these apparent differences in their electronic structures prompted us to revisit the aromaticity of compounds **1** ^***t*****Bu**^ **Bhp**, **A**, and **B**. One essential parameter is the magnetically induced ring current,[^57^, ^58^] which was estimated by GIMIC calculations[^57^, ^59^, ^60^, ^61^, ^62^] using proton‐substituted model systems (**1 H**, **AH**, **BH**). Additionally, benzene, naphthalene, indole, and borazine were computed as reference molecules (cf. SI, p. S53 ff). The current density susceptibility of selected systems is visualized in Figure 5 by streamline representations. The typical aromatic compounds benzene and indole clearly display a distinct diatropic π ring current, which encircles the ring system above and below the ring plane. In **AH**, on the other hand, only atomic vortices are found, whereas the current density of biradical **1H** is again very similar to benzene and indole.

**Figure 5:**
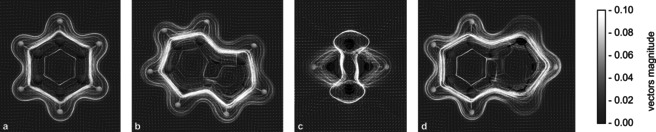
Streamline plot of the current density susceptibility^[57]^ for benzene (a), indole (b), [P(μ‐NH)]_2_ (**AH**) (c), and **1H** (d). For a color version see Figure S19.

The net induced ring current susceptibility can be quantified by integration of the current density (Table 2). The net induced current of **1H** (five‐membered ring: 11.2 nA T^−1^; six‐membered ring: 13.5 nA T^−1^) is very similar to the values of benzene and indole (≥12.1 nA T^−1^), whereas the values of **AH** (2.1 nA T^−1^) and **BH** (3.4 nA T^−1^) are significantly smaller. The NICS(1)_zz_ values (NICS=Nucleus‐Independent Chemical Shifts, Table 2),[^58^, ^63^, ^64^] which can also be used to describe aromaticity, exhibit the same trends as the magnetically induced currents. Thus, biradical **1** ^***t*****Bu**^ **Bhp** can be regarded as an aromatic system, while **A** and **B** are non‐aromatic, in accordance with earlier literature reports.^[65]^

**Table 2:** Net induced currents and NICS(1)_zz_ values of selected model systems. For fused ring systems, values are given for the five‐membered (⑤) and six‐membered part (⑥). Further information can be found in Table S16.

	C_6_H_6_	indole	**AH**	**BH**	**1H**
Net induced current [nA T^−1^]	12.1	13.1 (⑥) 12.1 (⑤)	2.1	3.5	11.2 (⑥) 13.5 (⑤)
					
NICS(1)_zz_ [ppm]	−30.2	−30.6 (⑥) −30.3 (⑤)	−9.4	−7.2	−24.9 (⑥) −31.1 (⑤)

In conclusion, compound **1** ^***t*****Bu**^ **Bhp** represents a new type of stable, P‐centered biradicals. It is, to the best of our knowledge, the first stable heteroindane‐1,3‐diyl. The biradical character of **1** ^***t*****Bu**^ **Bhp** is somewhat lower than the biradical character of other P‐centered biradicals, which is due to its aromatic stabilization. The self‐activation of **1Ter** yielding trimer **3Ter** demonstrates that this new substance class has potential for further activation chemistry, which was limited in case of previously reported five‐membered cyclic biradicals **B** owing to elimination problems.^[13]^ Reactivity studies and the investigation of the photochemistry of **1** ^***t*****Bu**^ **Bhp** are underway. Moreover, we plan to analyze the effect of substitutions in the aromatic backbone or replacement of P by heavier pnictogens on the reactivity and stability of the resulting biradicals.

## Conflict of interest

The authors declare no conflict of interest.

## Supporting information

As a service to our authors and readers, this journal provides supporting information supplied by the authors. Such materials are peer reviewed and may be re‐organized for online delivery, but are not copy‐edited or typeset. Technical support issues arising from supporting information (other than missing files) should be addressed to the authors.

Supplementary
