# Supplementary material for: Azadiphosphaindane‐1,3‐diyls: A Class of Resonance‐Stabilized Biradicals
Source: Angew Chem Int Ed Engl. 2020 Nov 19;60(3):1507–12. doi: 10.1002/anie.202011886 (PMC7839750; doi:10.1002/anie.202011886)
Supplement: Supplementary file 1 — Supplementary [file ANIE-60-1507-s001.pdf]

Angewandte Chemie
Eine Zeitschrift der Gesellschaft Deutscher Chemiker

Supporting Information

Azadiphosphaindane-1,3-diyls: A Class of Resonance-Stabilized Biradicals

Jonas Bresien, Dirk Michalik, Axel Schulz,* Alexander Villinger, and Edgar Zander*

anie_202011886_sm_miscellaneous_information.pdf

This file includes:

1 Experimental 3

2 Structure elucidation 6

3 Syntheses of starting materials 10

3.1 ${}^{tBu}BhpNH_2$ 10

3.2 $C_6H_4(PCl_2)_2$ 13

4 Syntheses of compounds 18

4.1 $C_6H_4P_2Cl_2N$-Dmp (**2Dmp**) 18

4.2 $C_6H_4P_2Cl_2N$-Ter (**2Ter**) 21

4.3 $C_6H_4P_2Cl_2N$-^{tBu}Bhp (**2^{tBu}Bhp**) 24

4.4 Reduction of **2Dmp** 27

4.5 Synthesis of **3Ter** 30

4.6 Synthesis of **1^{tBu}Bhp** 35

5 Additional spectroscopic details 39

5.1 ^{31}P NMR data of **3Ter** 39

6 Computational details 41

6.1 General remarks 41

6.2 Summary of calculated data 42

6.3 Comparison of the isomers of **2Dmp**, **2Ter** and **2^{tBu}Bhp** 43

6.4 Buried volumes and cone angles in **1Dmp**, **1Ter** and **1^{tBu}Bhp** 44

6.5 CASSCF computations of **A**, **B**, **1^{tBu}Bhp** and **3Ter** 46

6.6 Induced ring currents 53

6.7 Comparison of optimized and crystal structures 55

6.8 Optimized structures (.xyz-files) 61

7 References.. 85

1 Experimental

General Information: If not stated otherwise, all manipulations were carried out under oxygen- and moisture-free conditions under an inert atmosphere of argon using standard Schlenk or Drybox techniques. Solvents and reactants were obtained from commercial sources or synthesized as detailed in Table S1.

Table S1: Origin and purification of solvents and reactants.

Substance	Origin	Purification
CH_2Cl_2	local trade	purified according to literature procedure[1]<br>dried over P_4O_{10}, stored over CaH_2<br>freshly distilled and degassed (freeze-pump-thaw)
Et_2O, THF, benzene, toluene	local trade	dried over Na/benzophenone<br>freshly distilled prior to use
n-pentane	local trade	dried over Na/benzophenone/tetraglyme<br>freshly distilled prior to use
CD_2Cl_2	euriso-top	dried over P_4O_{10} and CaH_2<br>freshly distilled prior to use
C_6D_6	euriso-top	dried over Na<br>freshly distilled prior to use
1,2-dibromobenzene	abcr	dried over molecular sieves (4 Å), filtrated, distilled
4-*tert*-butylaniline	abcr	distilled
$DmpNH_2$	Arcos, 99%	dried over Na<br>freshly distilled prior to use
HCl	Linde, 99.8%	passed through SICAPENT®
$HNEt_2$	J.T. Baker	dried over Na<br>freshly distilled and degassed (freeze-pump-thaw)
Mg	abcr, 99.8% for Gringnards	stored under Ar, activated by stirring with glass stir bar for several weeks
NEt_3	Sigma Aldrich, 99%	dried over Na<br>freshly distilled prior to use
Ph_2COH	TCI	used as received
$ClP(NEt_2)$	synthesized[2]	distilled according to literature procedure
$TerNH_2$	synthesized[3,4]	recrystallized according to literature procedure
$ZnCl_2$	Riedel-de Haën	used as received

NMR spectra were recorded on Bruker spectrometers (AVANCE 250, AVANCE 300 or AVANCE 500) and were referenced internally to the deuterated solvent (^{13}C: CD_2Cl_2 δ_{ref} = 54.0 ppm, C_6D_6 δ_{ref} = 128.4 ppm), to protic impurities in the deuterated solvent (^{1}H: $CHDCl_2$ δ_{ref} = 5.32 ppm, C_6HD_5 δ_{ref} = 7.16 ppm) or externally (^{31}P: 85% H_3PO_4 δ_{ref} = 0 ppm). All measurements were carried out at ambient temperature unless denoted otherwise. NMR signals were assigned using experimental data (e.g. chemical shifts, coupling constants, integrals where applicable) in conjunction with computed NMR data (GIAO method, *cf.* Computational details, p. S41). The signs of $^{n}J(^{31}P,^{31}P)$ coupling constants were derived from the calculated spectra.

For NMR spectra simulation, the calculated and experimental ^{31}P NMR spectra were transferred to gNMR.[5] The full lineshape iteration procedure of gNMR was applied to match the calculated to the experimental spectrum.

IR spectra of crystalline and liquid samples were recorded on a Bruker Alpha II FT-IR spectrometer equipped with an ATR unit at ambient temperature under argon atmosphere.

Raman spectra of crystalline and liquid samples were recorded using a LabRAM HR 800 Horiba Jobin YVON Raman spectrometer equipped with an Olympus BX41 microscope with variable lenses. The samples were excited by an infrared laser (758 nm, 100 mW, air-cooled diode laser) or a red laser (633 nm, 17 mW, air-cooled HeNe laser). All measurements were carried out at ambient temperature unless stated otherwise.

Elemental analyses were obtained using an Elementar vario Micro cube CHNS analyser.

Melting points (uncorrected) were determined using a Stanford Research Systems EZ Melt at a heating rate of 20 °C/min.

DSC analyses were carried out at a heating rate of 5 °C/min using a Mettler-Toledo DSC 823e.

Mass spectra were recorded on a Thermo Electron MAT 95-XP sector field mass spectrometer using crystalline samples.

UV-Vis spectra were acquired on a Perkin-Elmer Lambda 19 UV-Vis spectrometer.

2 Structure elucidation

X-ray Structure Determination: X-ray quality crystals were selected in Fomblin YR-1800 perfluoroether (Alfa Aesar) at ambient temperature. The samples were cooled to 123(2) K during measurement. The data were collected on a Bruker D8 Quest diffractometer or a Bruker Kappa Apex II diffractometer using Mo K_{α} radiation (λ = 0.71073 Å). The structures were solved by iterative methods (SHELXT)[6] and refined by full matrix least squares procedures (SHELXL).[7] Semi-empirical absorption corrections were applied (SADABS).[8] All non-hydrogen atoms were refined anisotropically, hydrogen atoms were included in the refinement at calculated positions using a riding model.

Table S2: Crystallographic details.

Compound	**2Dmp**	**2^{tBu}Bhp**
Chem. formula	$C_{14}H_{13}Cl_2NP_2$	$C_{42}H_{37}Cl_2NP_2 \cdot C_4H_{10}O$
Formula weight [g/mol]	328.09	762.68
Color	colorless	colorless
Crystal system	monoclinic	triclinic
Space group	$P2_1$	$P1$
a [Å]	8.2407(8)	12.2513(19)
b [Å]	6.9612(7)	14.346(2)
c [Å]	13.2175(13)	24.404(4)
α [°]	90	89.579(5)
β [°]	97.107(2)	86.361(5)
γ [°]	90	71.945(5)
V [Å^3]	752.40(13)	4069.6(11)
Z	2	4
$\rho_{calcd.}$ [g/cm^3]	1.448	1.245
μ [mm^{-1}]	0.629	0.274
T [K]	123(2)	123(2)
Measured reflections	16385	243870
Independent reflections	6105	51838
Reflections with $I > 2\sigma(I)$	5847	40994
R_{int}	0.0209	0.0591
F(000)	336	1608
$R_1(R[F^2>2\sigma(F^2)])$	0.0239	0.0464
$wR_2(F^2)$	0.0591	0.1121
GooF	1.064	1.006
No. of Parameters	175	1916
CCDC #	2024625	2024626

Compound	**1^{tBu}Bhp**	**3Ter**
Chem. formula	$C_{42}H_{37}NP_2$	$C_{90}H_{87}N_3P_6{\cdot}4(C_6H_6)$
Formula weight [g/mol]	617.66	1708.87
Color	yellow	colorless
Crystal system	monoclinic	triclinic
Space group	$P2_1/n$	P
a [Å]	12.8998(4)	14.3024(7)
b [Å]	12.6520(4)	14.8132(7)
c [Å]	20.9865(7)	25.6310(13)
α [°]	90	75.897(3)
β [°]	100.6130(10)	81.204(3)
γ [°]	90	64.993(3)
V [Å^3]	3366.58(19)	4765.2(4)
Z	4	2
$\rho_{calcd.}$ [g/cm^3]	1.219	1.191
μ [mm^{-1}]	0.160	0.164
T [K]	123(2)	123(2)
Measured reflections	35370	125682
Independent reflections	8100	14913
Reflections with $I > 2\sigma(I)$	5476	10754
R_{int}	0.0679	0.0933
$F(000)$	1304	1812
$R_1(R[F^2>2\sigma(F^2)])$	0.0490	0.0689
w$R_2(F^2)$	0.1069	0.1868
GooF	1.035	1.058
No. of Parameters	409	894
CCDC #	2024627	2024628

Figure S1: Molecular structure of **2Dmp**. Ellipsoids are set at 50% probability (123 K). Selected bond lengths [Å] and angles [°]: C1–P1 1.806(2), C6–P2 1.808(2), P1–P2 2.9689(6), Cl1–P1 2.1035(5), Cl2–P2 2.0990(6), N1–P1 1.700(2), N1–P2 1.704(2), P1–N1–P2 121.41(7), C1–P1–P2–N1 −177.6(1), P1–C1–C6–P2 −0.7(2).

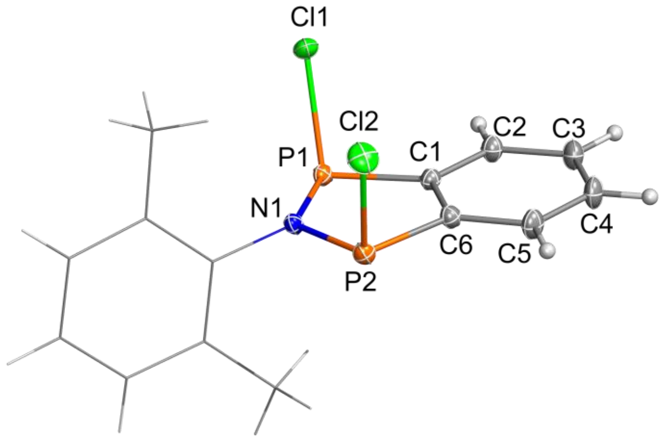

Figure S2: Molecular structure of **2^{tBu}Bhp**. Ellipsoids are set at 50% probability (123 K). Selected bond lengths [Å] and angles [°]: C37–P1 1.806(4), C42–P2 1.820(4), P1–P2 2.987(2), Cl1–P1 2.101(2), Cl2–P2 2.111(2), N1–P1 1.708(3), N1–P2 1.709(3), P1–N1–P2 121.9(2), C37–P1–P2–N1 −173.8(3), P1–C37–C24–P2 −0.2(4).

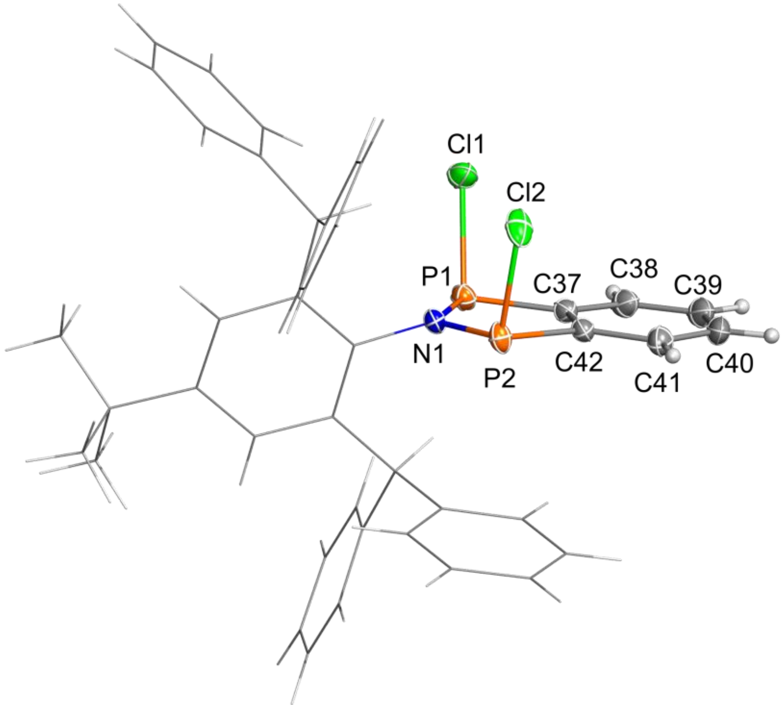

3 Syntheses of starting materials

3.1 ^{tBu}BhpNH$_2$

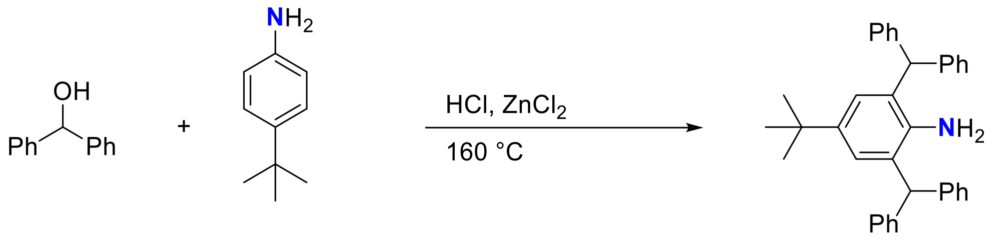

^{tBu}BhpNH$_2$ is prepared according to a slightly modified literature procedure.[9] The synthesis is not performed under oxygen- or moisture-free conditions. In a 1 L round-bottom flask, diphenylmethanol (46.73 g, 253.6 mmol), 4-*tert*-butylaniline (18.91 g, 126.7 mmol), $ZnCl_2$ (9.80 g, 71.9 mmol) and hydrochloric acid (37%, 15 mL) are mixed with a large stir bar. The flask is equipped with a reflux condenser and heated for 3 h at 160 °C in an oil bath. Afterwards, the oil bath is removed and CH_2Cl_2 (300 mL) is added through the reflux condenser to the still hot, liquid reaction mixture. The reaction mixture is neutralized by slow addition of an aqueous K_2CO_3 solution (25 g in 200 mL H_2O). This can lead to the formation of a colourless foam over the reaction solution, which is filtered off. The organic phase of the filtrate is separated and washed twice with water. Afterwards it is dried over $MgSO_4$ and concentrated in a rotary evaporator. The remaining solid is washed with cold MeOH. The product is recrystallized from boiling benzene (300 mL) and then dried *in vacuo* (1×10^{-3} mbar). Yield: 35.1 g (72.8 mmol, 57%).

Mp. 215 °C. CHN calc. (found) in %: C 89.77 (90.04); H 7.32 (7.13); N 2.91 (2.69). ^{1}H NMR (298 K, CD_2Cl_2, 500.1 MHz): δ = 0.99 (s, 9 H, p-*t*Bu), 3.33 (s, 2 H, N*H*$_2$), 5.46 (s, 2 H, C*H*Ph$_2$), 6.63 (s, 2 H, -C*H*), 7.13 (m, 8 H, *o*-C*H* (Ph)), 7.24 (m, 4 H, $^3J(^1H,^1H)$ = 7.3 Hz, *p*-C*H* (Ph)), 7.31 ppm (m, 8 H, $^3J(^1H, ^1H)$ = 7.3 Hz, *m*-C*H* (Ph)). $^{13}C\{^1H\}$ NMR (298 K, CD_2Cl_2, 125.8 MHz): δ = 31.6 (s, C(CH_3)$_3$), 34.4 (s, *C*(CH_3)$_3$), 53.3 (s, CHPh$_2$), 126.0 (s, *o*-*C*), 127.1

(s, *p-C* (Ph)), 128.9 (s, *o-C* (Ph)), 129.0 (s, *m-C*), 130.0 (s, *C-t*Bu), 140.2 (s, *m-C* (Ph)), 140.3 (s, *C*-NH_2), 143.6 ppm (s, *i-C* (Ph)). IR (ATR, 32 scans, cm^{-1}): = 3423 (w), 3359 (vw), 3101 (vw), 3081 (vw), 3060 (vw), 3027 (w), 2996 (vw), 2959 (w), 2903 (vw), 2864 (vw), 1628 (w), 1599 (w), 1492 (w), 1471 (m), 1447 (m), 1424 (w), 1391 (w), 1362 (w), 1339 (vw), 1321 (vw), 1294 (w), 1271 (w), 1255 (w), 1243 (w), 1201 (vw), 1189 (w), 1156 (vw), 1137 (vw), 1117 (vw), 1078 (w), 1030 (w), 1004 (vw), 973 (vw), 948 (vw), 925 (vw), 911 (w), 892 (w), 865 (w), 835 (w), 816 (vw), 760 (s), 736 (s), 696 (vs), 659 (m), 643 (m), 624 (s), 606 (s), 585 (m), 548 (w), 521 (w), 509 (w), 486 (w), 472 (m), 439 (w), 424 (w), 404 (w). Raman (633 nm, 20 s, 20 scans, cm^{-1}): = 3051 (2), 3028 (1), 3000 (1), 2966 (1), 2928 (1), 2903 (1), 2870 (1), 1600 (2), 1583 (1), 1559 (1), 1452 (1), 1299 (1), 1292 (1), 1243 (1), 1235 (1), 1224 (1), 1182 (1), 1171 (1), 1158 (1), 1120 (1), 1112 (1), 1075 (1), 1029 (4), 1001 (10), 951 (1), 922 (1), 889 (1), 863 (1), 833 (2), 764 (1), 744 (1), 673 (2), 648 (1), 633 (1), 617 (1), 605 (1), 558 (1), 510 (1), 494 (1), 482 (1), 453 (1), 439 (1), 417 (1), 363 (1), 299 (1), 286 (1), 252 (1), 233 (1), 217 (1), 182 (1), 160 (3), 149 (3), 139 (3). MS (EI, 70 eV, m/z (%)): 482 (26) $[M]^+$, 481 (80) $[M]^+$, 467 (41) $[M-Me]^+$, 466 $[M-Me]^+$, 167 (23) $[HCPh_2]^+$, 57 (14) $[tBu]^+$.

Figure S3: NMR, IR and Raman spectra of ^{tBu}BhpNH_2 (solvent signals indicated by asterisks).

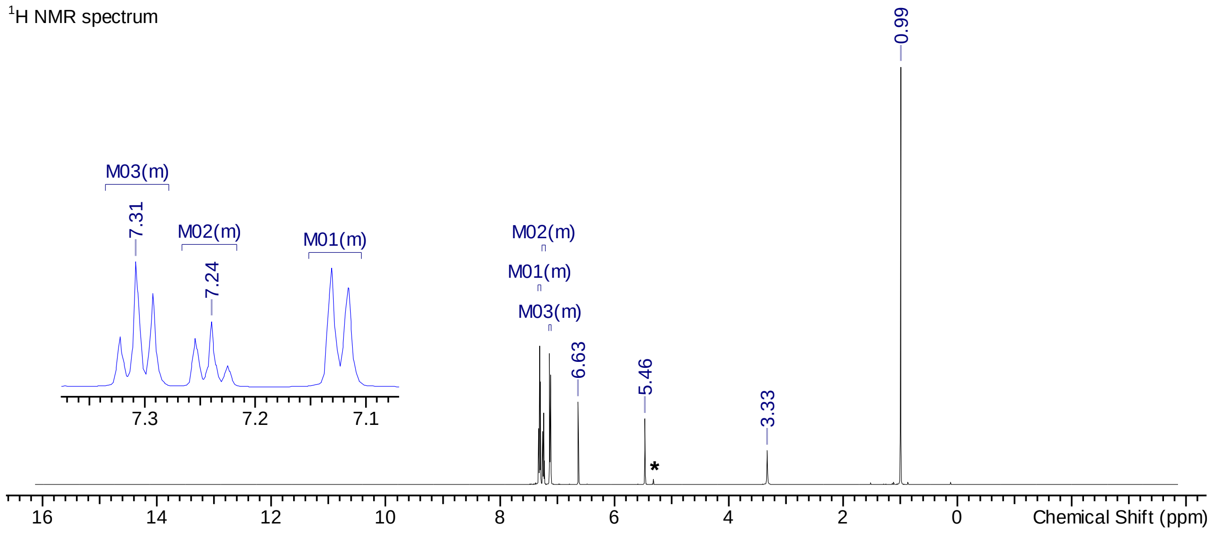

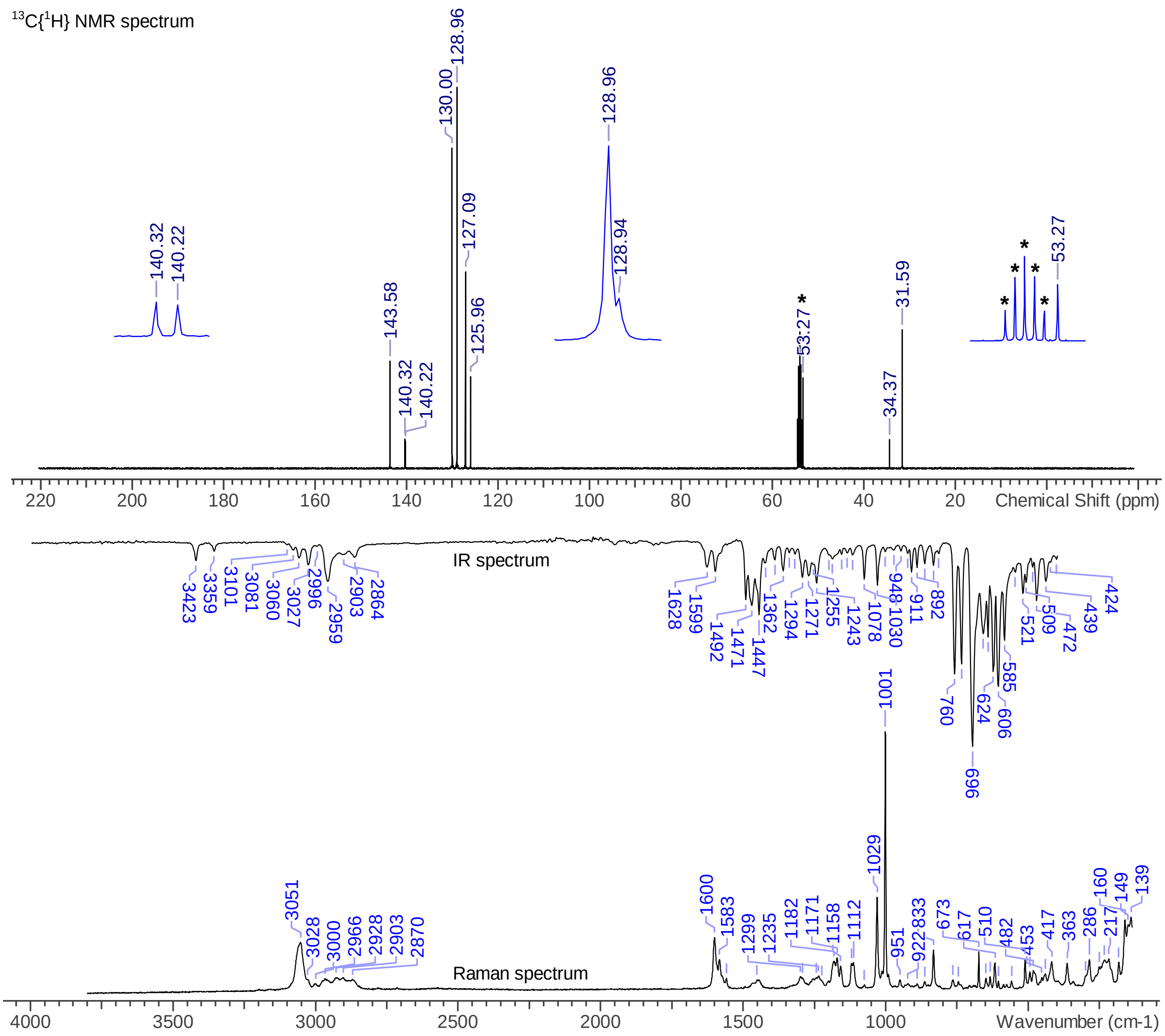

13C{1H} NMR spectrum
IR spectrum
Raman spectrum
Chemical Shift (ppm)
Wavenumber (cm-1)

3.2 $C_6H_4(PCl_2)_2$

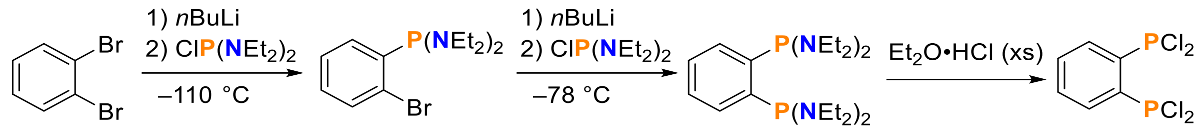

The synthesis of 1,2-bis(dichlorophosphino)benzene is carried out in three reaction steps, starting from 1,2-dibromobenzene according to a modified literature procedure by Xia, Ding and coworkers.[10] The reaction conditions for the first lithiation of 1,2-dibromobenzene are based on reports by Tamborski *et al.*[11]

Synthesis of $C_6H_4BrP(NEt_2)_2$

1,2-Dibromobenzene (14.21 g, 60.24 mmol) is dissolved in a mixture of Et_2O (100 mL) and THF (100 mL). The solution is cooled to −110 °C in an EtOH/N_2 cooling bath. At a constant temperature *n*BuLi (2.5 M in hexane, 24.6 mL, 61.5 mmol) is added over a period of 30 min. The solution is stirred for further 30 min at −110 °C. A solution of $ClP(NEt_2)_2$ (13.26 g, 62.9 mmol) in a mixture of THF (100 mL) and Et_2O (100 mL) is added via dropping funnel over a period of 60 min. The reaction solution turns dark green. It is kept at −110 °C for further 60 min and then left to warm to room temperature overnight. The workup is carried out under non-inert conditions: Ice water (200 mL) is added to the yellow solution, which is then extracted with *n*-pentane (400 mL). The organic phase is separated, dried over $MgSO_4$ and concentrated in the rotary evaporator. The residue is dried for 2 h *in vacuo* (1×10^{-3} mbar) at 55 °C (water bath), resulting in a yellowish viscous oil with a yield of 16.2 g (82%). The crude product is used without further purification.

$^{31}P\{^1H\}$ NMR (298 K, C_6D_6, 202.5 MHz): δ = 96.3 ppm (s). ^1H NMR (298 K, C_6D_6, 300.1 MHz): δ = 1.03 (t, $^3J(^1H,^1H)$ = 7.2 Hz, 12 H, CH_3), 3.03 (m, 8 H, CH_2), 6.76 (m, 1 H, C*H*), 7.07 (m, 1 H, C*H*), 7.45 (m, 1 H, C*H*), 7.51 ppm (m, 1 H, C*H*).

Synthesis of $C_6H_4(P(NEt_2)_2)_2$

$C_6H_4BrP(NEt_2)_2$ (13.98 g, 42.21 mmol) is dissolved in THF (100 mL) and cooled down to −78 °C in an *i*PrOH/N_2 cooling bath. *n*BuLi (2.5 M in hexane, 16.9 mL, 42.25 mmol) is added over a period of 30 min. After stirring for 30 min, a solution of $ClP(NEt_2)_2$ (8.89 g, 42.21 mmol) in THF (20 mL) is added slowly. The reaction solution is stirred at −78 °C for further 30 min and then slowly warmed to room temperature overnight. All volatiles are removed *in vacuo* (1×10^{-3} mbar). The residue is dissolved in *n*-pentane (100 mL) and filtered. The filtrate is dried for 2 h *in vacuo* (1×10^{-3} mbar) at 45 °C (water bath), resulting in a yellowish oil with a yield of 17.22 g (40.37 mmol, 96%). Again, the crude product is used without further purification.

$^{31}P\{^1H\}$ NMR (298 K, C_6D_6, 202.5 MHz): δ = 97.5 ppm (s). ^1H NMR (298 K, C_6D_6, 300.1 MHz): δ = 1.10 (t, $^3J(^1H,^1H)$ = 7.2 Hz, 24 H, CH_3), 3.10 (m, 16 H, CH_2), 7.26 (m, 2 H, C*H*), 7.78 ppm (m, 2 H, C*H*).

Synthesis of $C_6H_4(PCl_2)_2$

$C_6H_4(P(NEt_2)_2)_2$ (10.00 g, 23.44 mmol) is dissolved in Et_2O (50 mL). A solution of dry HCl in Et_2O (80 mL, 6.15 M, 492 mmol) is added over a period of 10 min at −40 °C. A colorless voluminous precipitate is formed. The solution is kept at −40 °C for 15 min and then stirred overnight at room temperature. The solvent is removed *in vacuo* (1×10^{-3} mbar) at 45 °C (water bath), and the residue is dried under the same conditions over a period of 2 h. Then *n*-pentane (40 mL) is added, and the suspension is extracted by repeated filtration and recondensation of the solvent (ca. four times). The filtrate is concentrated *in vacuo* (1×10^{-3} mbar), until the crude product is obtained as a yellowish oil. The product is purified by fractional distillation ($1\cdot\times10^{-3}$ mbar, 130 °C, oil bath). The distillate (the second of three fractions) is obtained as a clear colorless oil, yielding 5.27 g of $C_6H_4(PCl_2)_2$ (18.8 mmol, 80%).

Alternatively, the reaction can be carried out by passing dry HCl gas through a solution of $C_6H_4(P(NEt_2)_2)_2$ in *n*-hexane. For this purpose, a solution of $C_6H_4(P(NEt_2)_2)$ (17.31 g,

40.59 mmol) in *n*-hexane (250 mL) is placed in a 500 mL two-neck Schlenk flask equipped with a gas bubbler and gas inlet tube. HCl gas is dried by passing over Sicapent®, and is then bubbled through the stirred reaction mixture at 0 °C Over the entire reaction period, the flow rate of HCl is carefully adjusted so that no gas leaves the reaction vessel through the gas bubbler (i.e. HCl is quantitatively consumed by the reaction). During the reaction, $HNEt_2 \cdot HCl$ is formed as a voluminous colorless precipitate. The precipitate is filtered off and is then washed six times by recondensation of the solvent. Afterwards, the solvent of the filtrate is removed *in vacuo* ($1x10^{-3}$ mbar), whereupon the crude product remains as a yellowish oil. This is purified by fractional distillation ($1 \cdot 10^{-3}$ mbar, oilbath at 130 °C). The second fraction ($C_6H_4(PCl_2)_2$, 91-100 °C) is obtained as a clear colorless oil, yielding 9.54 g (34.1 mmol, 84%).

CHN calc. (found) in %: C 25.75 (28.22), H 1.44 (1.80). Due to minor impurities (see ^{1}H and $^{13}C\{^{1}H\}$ NMR spectra) no better measurement results could be achieved. The product can however be used in follow-up reactions. $^{31}P\{^{1}H\}$ NMR (298 K, CD_2Cl_2, 202.5 MHz): δ = 151.9 ppm (s). ^{1}H NMR (298 K, CD_2Cl_2, 250.1 MHz): δ = 7.77 (m, 2 H, C*H*), 8.26 ppm (m, 2 H, C*H*). $^{13}C\{^{1}H\}$ NMR (298 K, CD_2Cl_2, 125.8 MHz): δ = 131.2 (m, *C*H), 134.2 (s, *C*H), 144.7 ppm (m, *C*P). IR (ATR, 32 scans, cm^{-1}): = 3052.59 (vw), 1572.67 (w), 1436.63 (w), 1290.29 (vw), 1166.62 (vw), 1131.58 (w), 1100.66 (s), 1036.77 (w), 853.32 (w), 750.26 (s), 719.35 (m), 696.67 (vw), 661.63 (w), 583.31 (w), 482.31 (vs), 463.76 (vs), 430.78 (s). Raman (633 nm, 20 s, 20 scans, cm^{-1}): = 155 (3), 202 (2), 241 (3), 264 (1), 278 (2), 319 (2), 394 (1), 434 (2), 457 (2), 471 (3), 513 (10), 662 (2), 694 (1), 717 (1), 812 (1), 1027 (3), 1038 (4), 1101 (3), 1125 (2), 1167 (2), 1218 (2), 1293 (2), 1471 (2), 1555 (2), 1573 (4), 1590 (3), 3056 (2), 3115 (1), 3146 (1). MS (CI, pos., isobutane, m/z): 283 $[M+H]^+$, 281 $[M+H]^+$, 280 $[M+H]^+$, 245 $[M-Cl]^+$, 243 $[M-Cl]^+$.

Figure S4: NMR, IR and Raman spectra of $C_6H_4(PCl_2)_2$ (solvent signals indicated by asterisks).

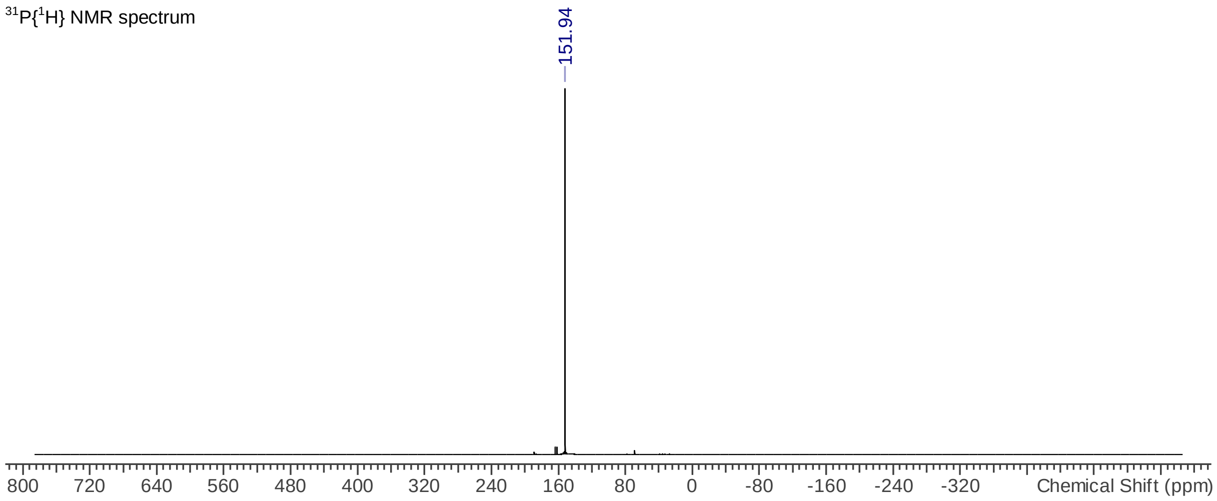

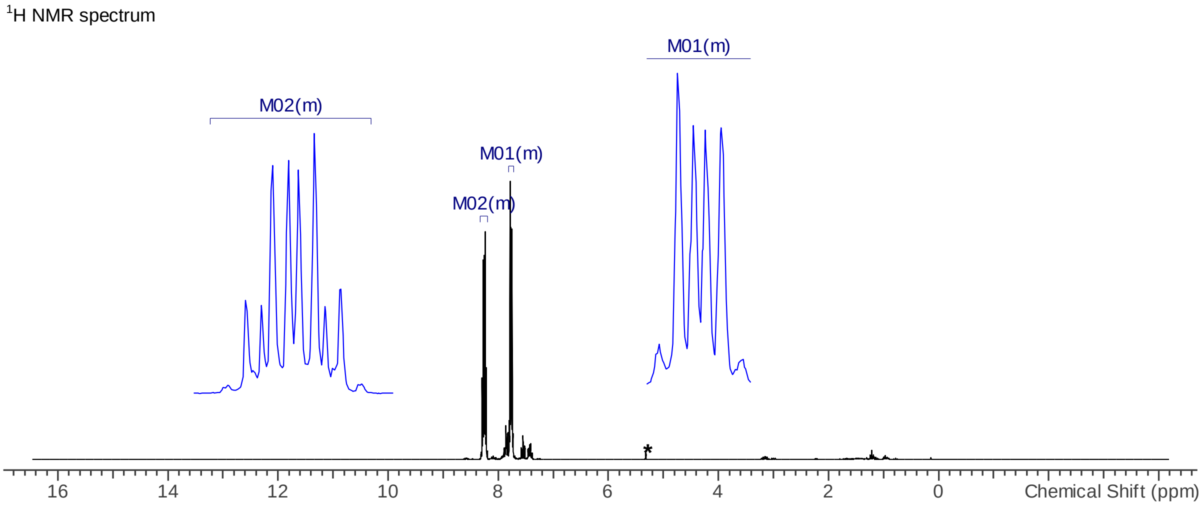

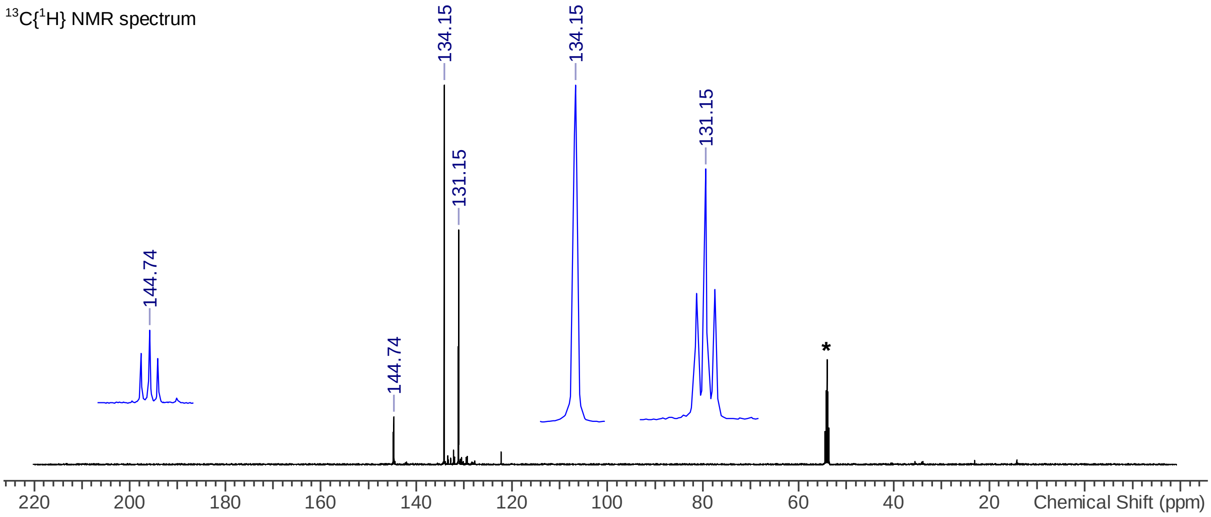

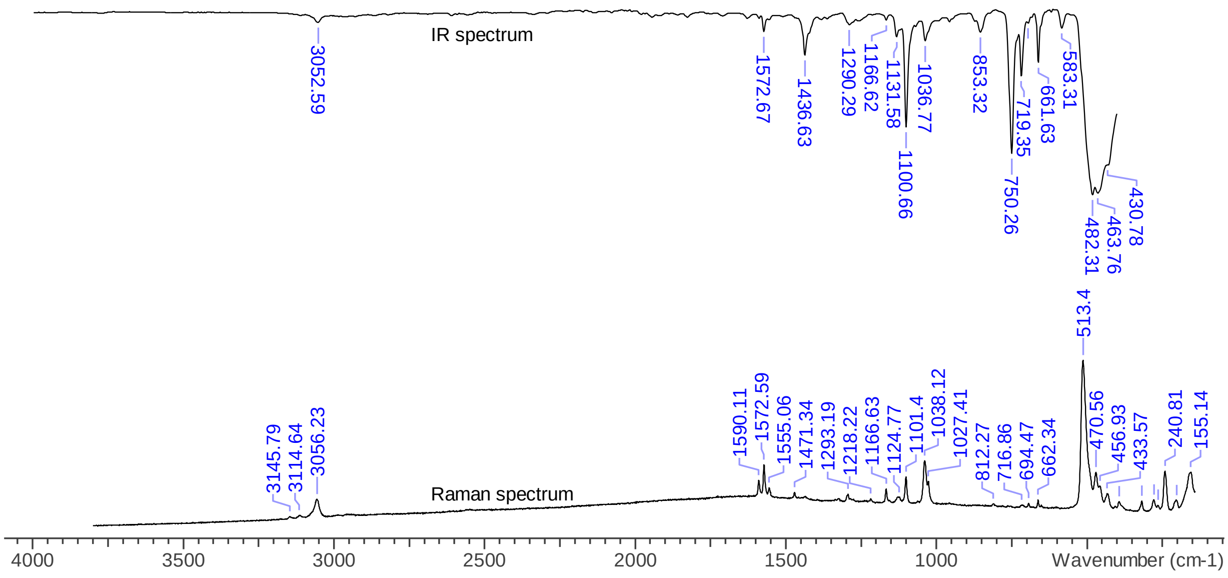
IR spectrum
3052.59
1572.67
1436.63
1290.29
1166.62
1131.58
1100.66
1036.77
853.32
750.26
719.35
661.63
583.31
482.31
463.76
430.78
Raman spectrum
3145.79
3114.64
3056.23
1590.11
1572.59
1555.06
1471.34
1293.19
1218.22
1166.63
1124.77
1101.4
1038.12
1027.41
812.27
716.86
694.47
662.34
513.4
470.56
456.93
433.57
240.81
155.14
4000
3500
3000
2500
2000
1500
1000
Wavenumber (cm-1)

4 Syntheses of compounds

4.1 $C_6H_4P_2Cl_2N$-Dmp (2Dmp)

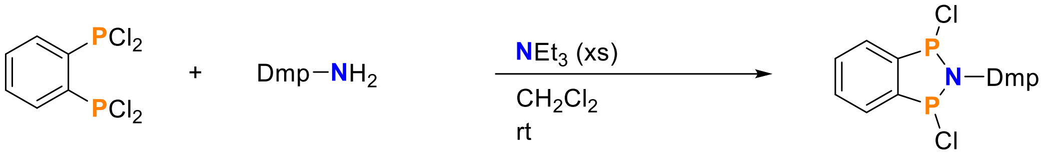

A solution of $DmpNH_2$ (368 mg, 3.04 mmol) in CH_2Cl_2 (15 mL) is added to a stirred solution of 1,2-bis(dichlorophosphino)benzene (850 mg, 3.04 mmol) and NEt_3 (3073 mg, 60.74 mmol) in CH_2Cl_2 (15 mL) at ambient temperature. After a reaction time of 21 h, the solvent is removed *in vacuo* (1×10^{-3} mbar) and the residue is dried *in vacuo* (1×10^{-3} mbar) at 45 °C (water bath) for three hours. The yellowish residue is extracted with Et_2O (20 mL) and the insoluble solids are filtered off. The filtrate is dried *in vacuo* ($1x10^{-3}$ mbar), leaving a colorless solid. The residue is dissolved in CH_2Cl_2 (3 mL) at room temperature. Cooling the solution down to 0 °C leads to crystallization of the colorless product. The supernatant is removed by syringe and can be used for crystallization of further fractions. The crystals are dried for 2 h *in vacuo* ($1x10^{-3}$ mbar) at 45 °C (water bath). Yield: 563 mg, (1.72 mmol, 56%).

Isomeric ratio (*cis*: *trans*): 92, 8.[1]

Single crystals of **2Dmp** suitable for SC-XRD were obtained as described above.

Mp. 169 °C. CHN calc. (found) in %: C 51.25 (51.39); H 3.99 (4.11); N 4.27 (4.23). $^{31}P\{^1H\}$ NMR (298 K, CD_2Cl_2, 121.5 MHz): δ = 146.5 (s, *cis* isomer), 171.2 ppm (s, *trans* isomer). ^1H NMR (298 K, CD_2Cl_2, 300.1 MHz): 2.04 (s, 3 H, CH_3 (*cis* isomer)), 2.52 (t, $^5J(^{31}P,^1H)$ = 1.6 Hz, 6 H, CH_3 (*trans* isomer)), 2.64 (t, $^5J(^{31}P,^1H)$ = 1.5 Hz, 3 H, CH_3 (*cis* isomer)),

[1] Determined from the integral ratio of the methyl group signals in the ^1H NMR spectrum.

7.10-7.17 (m, 1 H, *p*-C*H* (Dmp, *cis* and *trans* isomer)), 7.19-7.22 (m, 2 H, *m*-C*H* (Dmp, *cis* and *trans* isomer)), 7.68-7.75 (m, 2 H, C*H* (C_6H_4) (*cis* and *trans* isomer)), 7.90-7.98 ppm (m, 2 H, C*H* (C_6H_4) (*cis* and *trans* isomer)). $^{13}C\{^{1}H\}$ NMR (298 K, CD_2Cl_2, 75.5 MHz): δ = 19.6 (t, $J(^{13}C, ^{31}P)$ = 7 Hz, CH_3 (Dmp)), 20.7 (s, CH_3 (Dmp)), 128.8 (m, CH (Dmp)), 129.1 (m, CH (C_6H_4)), 129.2 (s, CH (Dmp)), 130.1 (t, $J(^{13}C,^{31}P)$ = 2 Hz, CH (Dmp)), 132.1 (m, (C_6H_4)), 136.9 (t, $J(^{13}C,^{31}P)$ = 4 Hz, quaternary *C* (Dmp)), 138.8 (t, $J(^{13}C,^{31}P)$ = 4 Hz, quaternary *C* (Dmp)), 139.6 (t, $J(^{13}C,^{31}P)$ = 17 Hz, quaternary *C* (Dmp)), 148.8 ppm (m, quaternary *C* (C_6H_4)). IR (ATR, 32 scans, cm^{-1}): = 3061 (vw), 2923 (vw), 1830 (vw), 1635 (vw), 1587 (vw), 1558 (vw), 1463 (w), 1447 (w), 1433 (m), 1377 (w), 1286 (w), 1259 (w), 1193 (m), 1162 (w), 1134 (vw), 1117 (m), 1097 (w), 1070 (w), 1053 (w), 1029 (vw), 985 (vw), 971 (vw), 905 (s), 878 (m), 771 (m), 756 (s), 736 (w), 726 (m), 676 (w), 587 (w), 557 (w), 536 (m), 517 (w), 491 (m), 474 (s), 449 (s), 445 (vs), 423 (s). Raman (633 nm, 20 s, 20 scans, cm^{-1}): = 3120 (1), 3067 (1), 3044 (1), 2916 (2), 2862 (1), 2734 (1), 2577 (1), 1587 (2), 1558 (1), 1467 (1), 1448 (1), 1433 (1), 1380 (1), 1301 (1), 1288 (1), 1259 (2), 1196 (1), 1172 (1), 1163 (1), 1134 (1), 1119 (6), 1099 (2), 1054 (1), 1042 (1), 1029 (5), 1008 (1), 988 (1), 906 (1), 893 (1), 877 (1), 756 (1), 734 (1), 721 (1), 676 (1), 588 (4), 557 (1), 536 (1), 517 (1), 505 (1), 491 (2), 476 (10), 453 (6), 446 (3), 422 (4), 385 (6), 341 (1), 285 (10), 230 (1), 210 (4), 179 (2), 151 (2). MS (CI, pos., isobutane, m/z): 328 $[M+H]^+$, 292 $[M-Cl]^+$.

Figure S5: NMR, IR and Raman spectra of **2Dmp** (solvent signals indicated by asterisks).

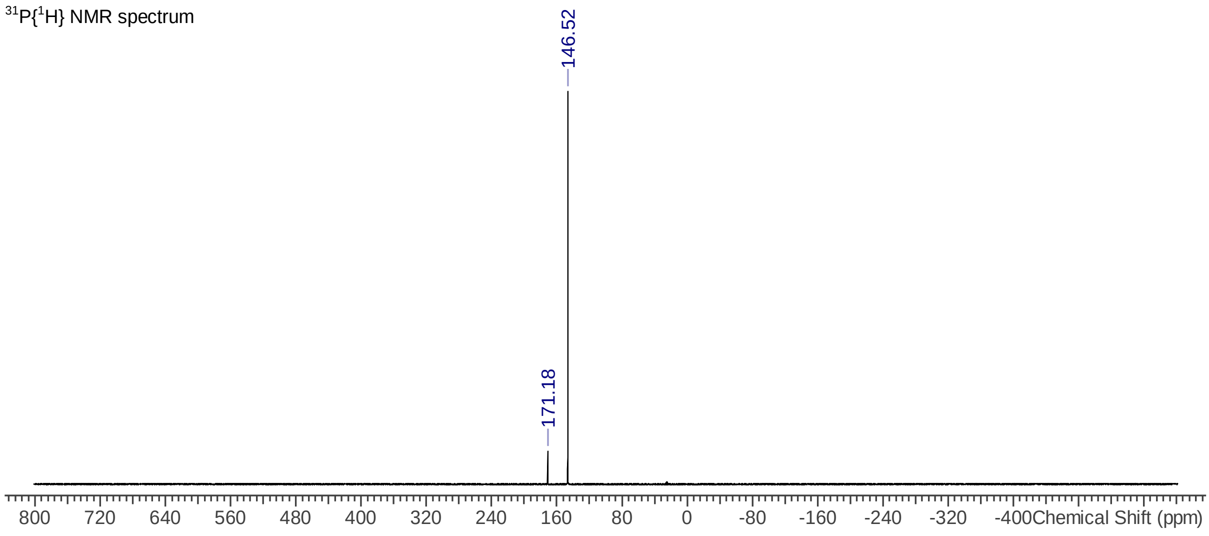

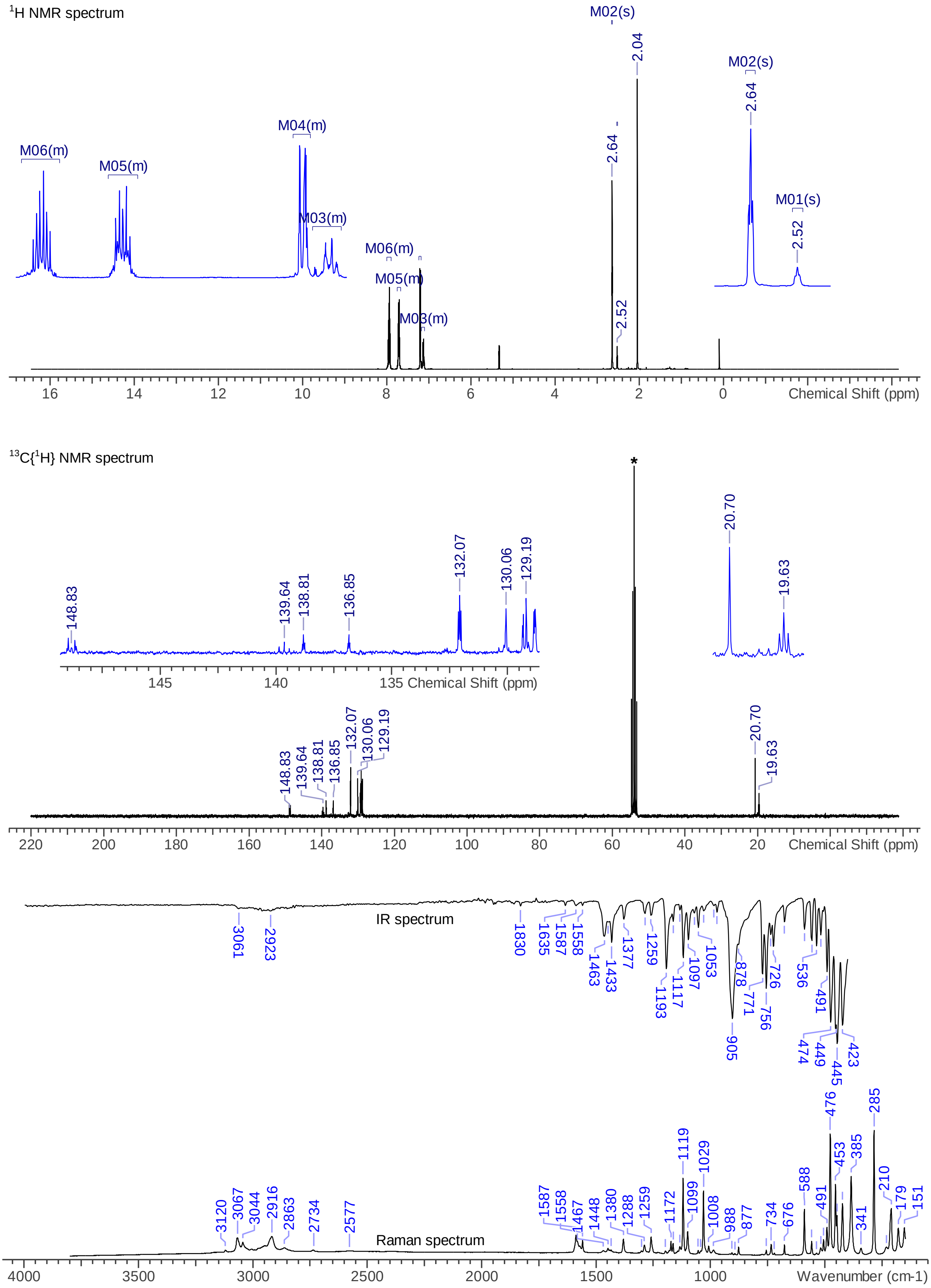
1H NMR spectrum
M06(m)
M05(m)
M04(m)
M03(m)
M02(s)
M01(s)
2.64
2.04
2.52
Chemical Shift (ppm)
13C{1H} NMR spectrum
148.83
139.64
138.81
136.85
132.07
130.06
129.19
20.70
19.63
Chemical Shift (ppm)
IR spectrum
3061
2923
1830
1635
1587
1558
1463
1433
1377
1259
1193
1117
1097
1053
905
878
771
756
726
536
491
474
449
445
423
Raman spectrum
3120
3067
3044
2916
2863
2734
2577
1587
1558
1467
1448
1380
1288
1259
1172
1119
1099
1029
1008
988
877
734
676
588
491
476
453
385
341
285
210
179
151
Wavenumber (cm-1)

4.2 $C_6H_4P_2Cl_2N$-Ter (2Ter)

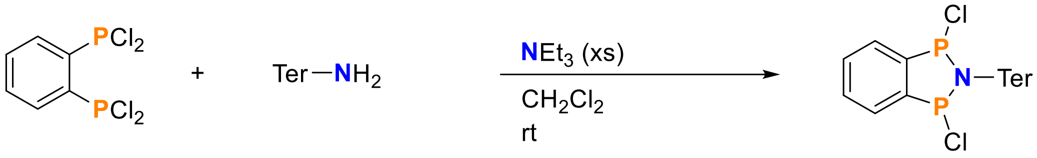

A solution of of $TerNH_2$ (632 mg, 1.92 mmol) in CH_2Cl_2 (7.5 mL) is added to a stirred solution of 1,2-bis(dichlorophosphino)benzene (537 mg, 1.92 mmol) and NEt_3 (3880 mg, 38.24 mmol) in CH_2Cl_2 (10 mL) at ambient temperature. After a reaction time of 2 h, the solvent is removed *in vacuo* (1×10^{-3} mbar) and the residue is dried *in vacuo* (1×10^{-3} mbar) at 45 °C (water bath) for two hours. To the yellowish residue is extracted with Et_2O (20 mL) and the insoluble solids are filtered off. The filtrate is dried *in vacuo* (1×10^{-3} mbar), resulting in a colorless solid, which is recrystallized from CH_2Cl_2/MeCN, yielding small colorless crystals. The crystals are isolated and dried for 2 h *in vacuo* (1×10^{-3} mbar) at 45 °C (water bath). Yield 333 mg (0.62 mmol, 32%.).

Mp. 181 °C. CHN calc. (found) in %: C 67.16 (67.37); H 5.45 (5.14); N 2.61 (2.46). $^{31}P\{^1H\}$ NMR (298 K, CD_2Cl_2, 101.3 MHz): δ = 146.8 (s) ppm. ^1H NMR (298 K, CD_2Cl_2, 500.1 MHz): δ = 1.83-2.40 (superimposed signals, 18 H, C*H*$_3$), 6.40-7.01 (4 H, C*H* (Mes)), 7.17 (d, 2 H , $^3J(^1H,^1H)$ = 7.4 Hz, *m*-C*H* (Ter)), 7.24-7.42 ppm (superimposed signals, 5 H, C*H* (C_6H_4) and *p*-C*H* (Ter)). $^{13}C\{^1H\}$ NMR (298 K, CD_2Cl_2, 125.8 MHz): δ = 20.4-22.1 (Me signals), 127.2-131.3 (CH signals), 137.2-150.7 ppm (quaternary *C*). IR (ATR, 32 scans, cm^{-1}): = 2968 (w), 2943 (w), 2914 (w), 2855 (w), 2731 (vw), 1733 (vw), 1610 (w), 1562 (vw), 1486 (vw), 1453 (w), 1435 (w), 1410 (m), 1377 (w), 1288 (vw), 1268 (vw), 1251 (vw), 1200 (m), 1162 (vw), 1144 (vw), 1134 (vw), 1119 (m), 1078 (w), 1053 (w), 1031 (w), 1012 (vw), 936 (vw), 897 (s), 849 (m), 804 (m), 783 (w), 752 (m), 736 (w), 693 (w), 676 (w), 600 (w), 575 (vw), 559 (vw), 530 (m), 497 (m), 480 (vs), 466 (s), 449 (m), 431 (s), 406 (m). Raman (633 nm, 30 s, 20 scans, cm^{-1}): = 3061 (3), 3043 (3), 3009 (2), 2919 (4), 2857 (2), 2734 (2), 1611 (5), 1578 (5), 1563 (4), 1486 (3), 1445 (3), 1382 (5), 1378 (4), 1306 (8), 1287 (3), 1268 (3), 1201 (4), 1188 (4), 1164 (4), 1121 (7), 1080 (4), 1030 (5), 1012 (4), 1008 (4), 946 (3), 900 (3), 735 (3), 693 (3), 677 (4), 601 (3), 578 (8), 562 (5), 525 (5), 508

(3), 501 (4), 481 (7), 466 (5), 448 (3), 431 (5), 423 (5), 408 (7), 397 (10), 340 (5), 295 (6), 260 (3), 256 (3), 237 (5), 207 (3), 169 (5), 159 (4), 154 (4), 136 (7). MS (CI, pos., isobutane, m/z): 536 $[M+H]^+$, 500 $[M-Cl]^+$.

Figure S6: NMR, IR and Raman spectra of **2Ter** (solvent signals indicated by asterisks).

$^{31}P\{^1H\}$ NMR spectrum

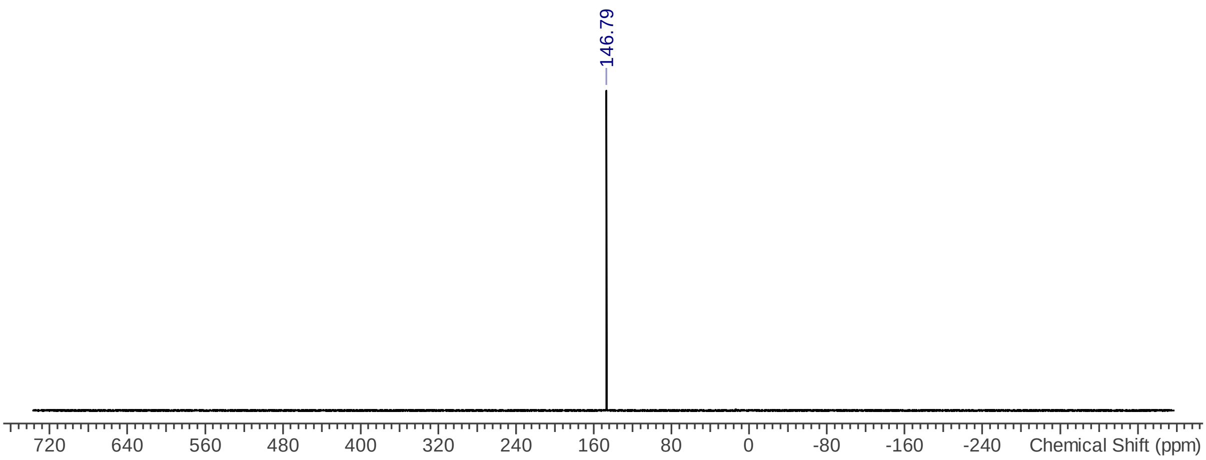

^1H NMR spectrum

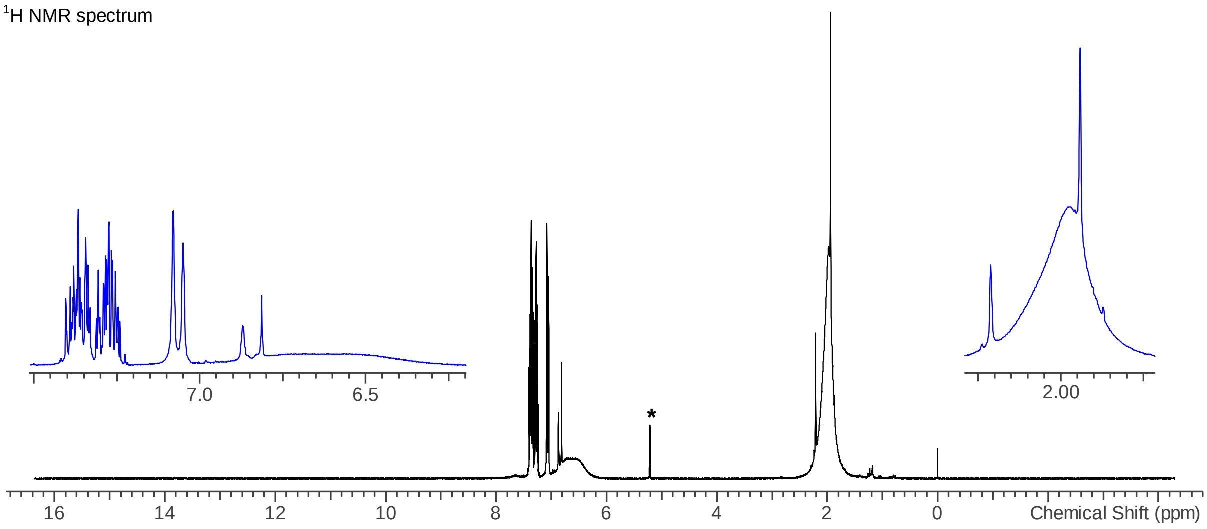

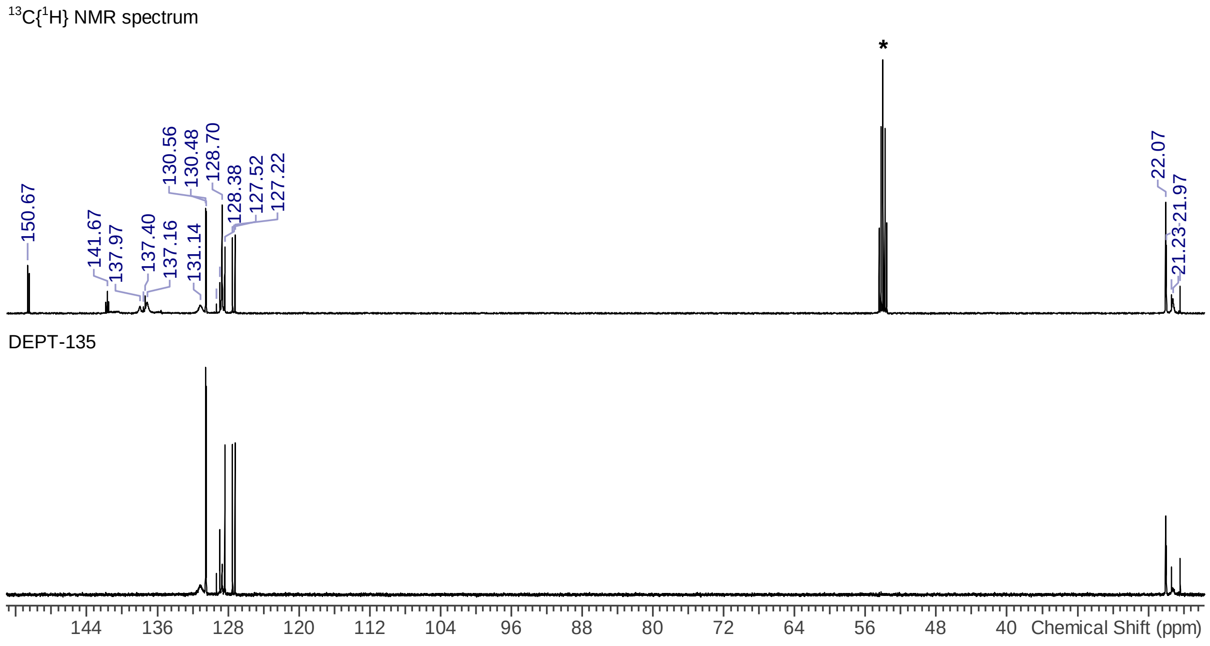

$^{13}C\{^1H\}$ NMR spectrum
*
150.67
141.67
137.97
137.40
137.16
131.14
130.56
130.48
128.70
128.38
127.52
127.22
22.07
21.97
21.23
DEPT-135
144
136
128
120
112
104
96
88
80
72
64
56
48
40
Chemical Shift (ppm)

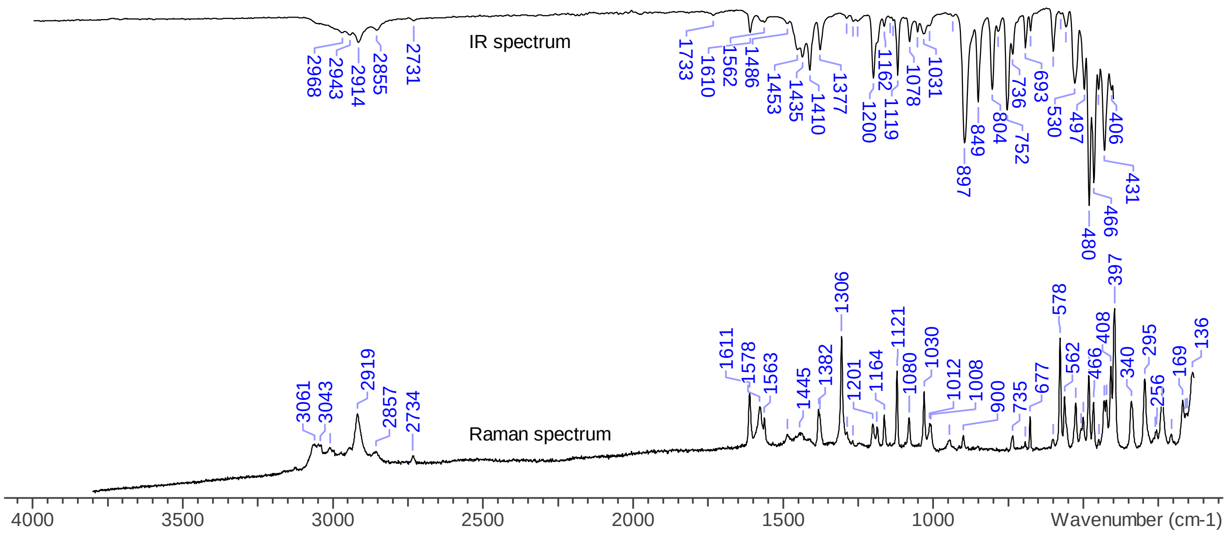

IR spectrum
2968
2943
2914
2855
2731
1733
1610
1562
1486
1453
1435
1410
1377
1200
1162
1119
1078
1031
897
849
804
736
752
693
530
497
480
466
431
406
Raman spectrum
3061
3043
2919
2857
2734
1611
1578
1563
1445
1382
1306
1201
1164
1121
1080
1030
1012
1008
900
735
677
578
562
466
408
397
340
295
256
169
136
4000
3500
3000
2500
2000
1500
1000
Wavenumber (cm-1)

4.3 $C_6H_4P_2Cl_2N$-^{tBu}Bhp (2^{tBu}Bhp)

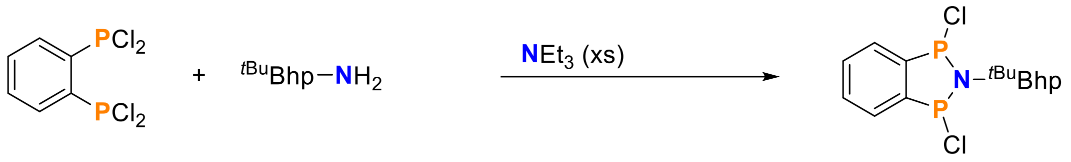

A solution of ^{tBu}BhpNH$_2$ (123 mg, 456 mmol) 5.85 g, 12.1 mmol) in CH_2Cl_2 (40 mL) is added to a stirred solution of 1,2-bis(dichlorophosphino)benzene (3.40 g, 12.1 mmol) and NEt_3 (24.6 g, 244 mmol) in CH_2Cl_2 (70 mL) at 0 °C (ice/water bath) over a period of 10 minutes, resulting in a yellowish suspension. The mixture is warmed to ambient temperature and stirred overnight. All volatiles are removed *in vacuo* (1×10^{-3} mbar) and the residue is dried *in vacuo* (1×10^{-3} mbar) at 45 °C (water bath) for one hour. Et_2O (100 mL) is added, and the suspension is extracted by repeated filtration and recondensation of the solvent (ca. six times). The yellowish filtrate is concentrated at 30 °C (water bath) to incipient crystallization. Crystallization overnight at ambient temperature yields colorless crystals of the product. The supernatant is removed by syringe and can be used for crystallization of further fractions. The crystals are dried *in vacuo* ($1x10^{-3}$ mbar) at 45 °C (water bath) for 2 h. Yield: 7.26 g (10.5 mmol, 86%).

Single crystals of **2^{tBu}Bhp** · Et_2O suitable for SC-XRD were obtained as described above.

Mp. 158 °C (dec.). CHN calc. (found) in %: C 73.26 (73.22); H 5.42 (5.80); N 2.03 (1.90). $^{31}P\{^1H\}$ NMR (298 K, CD_2Cl_2, 202.5 MHz): δ = 148.9 ppm (s). ^1H NMR (298 K, CD_2Cl_2, 500.1 MHz): δ = 1.08 (s, 9 H, C(C*H*$_3$)$_3$), 5.23 (t, $J(^{31}P,^1H)$ = 3.2 Hz, 1 H, C*H*Ph$_2$), 6.46 (s, 1 H), 6.75 (d, *J* = 7.5 Hz, 4 H), 6.88 (d, *J* = 2.1 Hz, 1 H), 7.15-7.24 (superimposed signals, 13 H), 7.30 (t, *J* = 7.5 Hz, 4 H), 7.63 (m, 2 H), 7.73 ppm (m, 2 H) (aromatic signals not assigned).$^{13}C\{^1H\}$ NMR (298 K, CD_2Cl_2, 125.8 MHz): δ = 31.2 (s, C(CH$_3$)$_3$), 35.1 (s, *C*(CH$_3$)$_3$), 51.7 (s, CHPh$_2$), 54.6 (t, $J(^{31}P,^{13}C)$ = 6 Hz), 126.6-131.7 (aromatic CH) 137.9-150.9 ppm (quaternary *C*). IR (ATR, 64 scans, cm^{-1}): = 3084 (vw), 3059 (w), 3026 (w), 2958 (w), 2929 (w), 2902 (w), 2867 (w), 1599 (w), 1581 (vw), 1492 (w), 1476 (w), 1445

(m), 1412 (w), 1395 (w), 1362 (w), 1336 (vw), 1323 (vw), 1290 (w), 1255 (w), 1237 (w), 1204 (w), 1181 (w), 1113 (m), 1076 (w), 1051 (w), 1031 (w), 1002 (vw), 895 (m), 866 (w), 851 (w), 812 (vw), 752 (m), 699 (vs), 674 (w), 649 (w), 633 (w), 620 (w), 606 (m), 587 (m), 577 (m), 559 (m), 532 (vw), 495 (m), 480 (s), 453 (m), 443 (s), 423 (m). Raman (633 nm, 10 s, 20 scans, cm^{-1}): = 3052 (3), 3027 (1), 3003 (1), 2980 (1), 2968 (1), 2927 (1), 2905 (1), 2890 (1), 2872 (1), 1603 (2), 1585 (1), 1577 (1), 1561 (1), 1452 (1), 1302 (1), 1258 (1), 1246 (1), 1238 (1), 1185 (1), 1173 (1), 1161 (1), 1123 (1), 1115 (1), 1032 (3), 1016 (1), 1004 (10), 993 (1), 952 (1), 867 (1), 836 (2), 768 (1), 748 (1), 708 (1), 676 (1), 651 (1), 636 (1), 620 (1), 589 (1), 579 (1), 562 (1), 513 (1), 498 (1), 485 (1), 479 (1), 455 (1), 442 (1), 421 (1), 367 (1), 303 (1), 289 (1), 266 (1), 249 (1), 236 (1), 229 (1), 223 (1), 211 (1), 185 (1), 164 (1), 152 (1), 142 (2). MS (CI, pos., isobutane, m/z): 610 $[M-C_6H_4]^+$, 652 $[M-Cl]^+$, 678 $[M+H]^+$.

Figure S7: NMR, IR and Raman spectra of $\mathbf{2}^{t\mathbf{Bu}}$**Bhp** (solvent signals indicated by asterisks).

$^{31}P\{^1H\}$ NMR spectrum

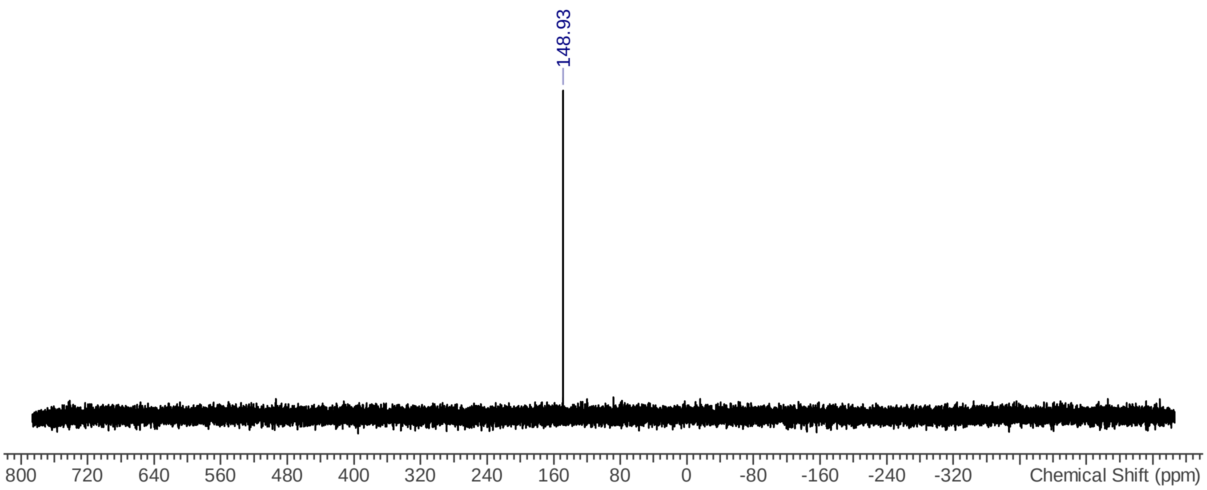

^{1}H NMR spectrum

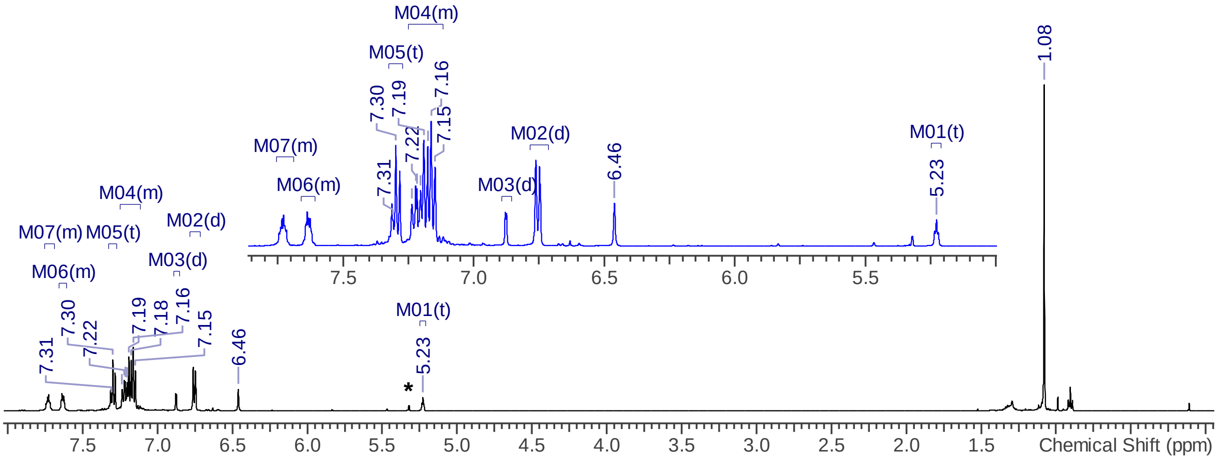

$^{13}C\{^{1}H\}$ NMR spectrum

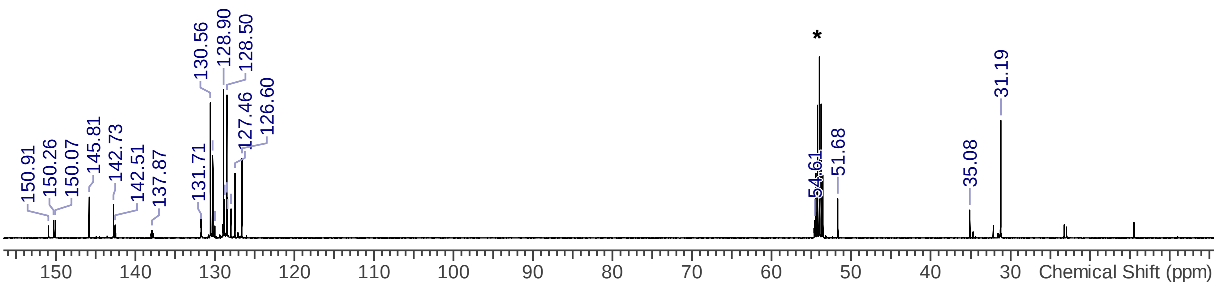

DEPT-135

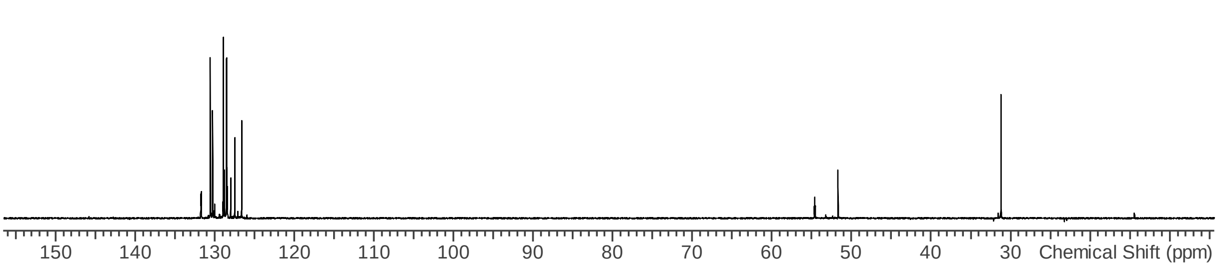

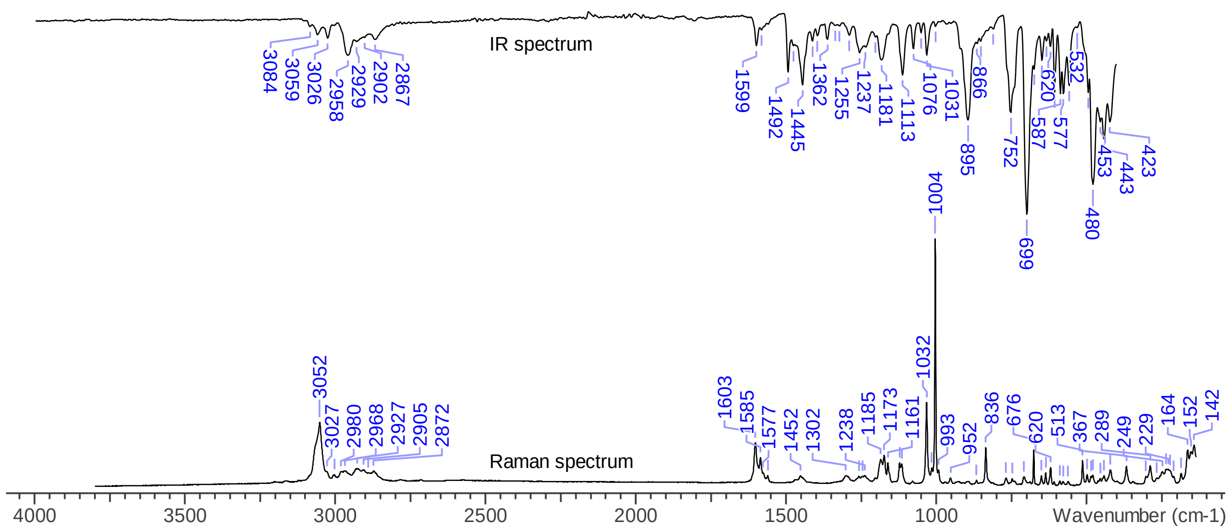

4.4 Reduction of 2Dmp

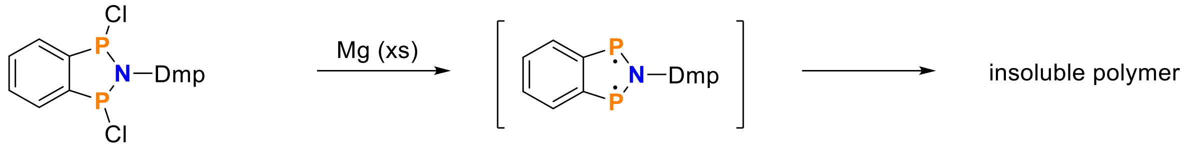

Mechanically activated magnesium chips (*cf*. Table S1; 210 mg, 8.64 mmol) and **2Dmp** (100 mg, 0.31 mmol) are combined in a Schlenk flask equipped with a glass covered magnetic stir bar. *Attention: It is paramount to ensure that no grease finds its way into the reaction vessel. Otherwise the reduction is not reproducible.* THF (5 mL) is added while stirring. The reaction solution turns orange within a few minutes and is stirred for 24 h. As an intermediate, the biradical **1Dmp** can be observed by $^{31}P\{^1H\}$ NMR spectroscopy (see Figure S8, p. 28). After one day reaction time **2Dmp** and **1Dmp** are completely reacted to a red polymer.

Intermediate:

$^{31}P\{^1H\}$ NMR (298 K, C_6D_6, 121.5 MHz): δ = 279.5 ppm (s).

To isolate the polymer, the reaction is carried out under modified conditions. Mechanically activated magnesium chips (*cf*. Table S1; 1 000 mg, 41.13 mmol) and **2Dmp** (250 mg, 0.765 mmol) are combined in a Schlenk flask equipped with a glass covered magnetic stir bar. THF (30 mL) is added while stirring. The reaction solution turns orange within a few minutes and a yellowish precipitate is formed. The reaction mixture is stirred for 3 h. Magnesium chips and the yellowish precipitate are filtered off. The solvent of the filtrate is reduced to half (ca. 15 mL) *in vacuo* ($1x10^{-3}$ mbar). The solution is stirred for two weeks at room temperature, while an orange precipitate is formed. The precipitate is separated by filtration and then washed six times by re-condensation of the solvent. Afterwards benzene (10 mL) is added to the precipitate, and the resulting suspension is filtrated. The remaining red oligo- /polymer is dried *in vacuo* (1×10^{-3} mbar) at 60 °C (water bath) for four hours. Yield: ca. 8 mg.

Product:

CHN calc. (found) in %: C 65.38 (65.19), H 5.09 (5.34), N 5.45 (5.28). IR (ATR, 64 scans, cm^{-1}): = 3044 (w), 3013 (w), 2976 (w), 2912 (w), 1587 (vw), 1574 (vw), 1465 (w), 1428 (m), 1372 (w), 1255 (w), 1195 (m), 1162 (w), 1096 (m), 983 (w), 896 (w), 859 (vs), 767 (m), 742 (s), 717 (s), 674 (m), 573 (m), 536 (s), 505 (m), 484 (m), 464 (m), 441 (m), 422 (m). Raman (633 nm, 10 s, 20 scans, cm^{-1}): =3046 (1), 2919 (1), 1767 (1), 1576 (1), 1552 (1), 1435 (4), 1377 (1), 1279 (1), 1259 (1), 1203 (1), 1171 (1), 1160 (1), 1096 (10), 1063 (1), 1046 (1), 1033 (2), 992 (1), 882 (1), 730 (1), 721 (1), 675 (1), 576 (1), 553 (1), 542 (1), 508 (1), 485 (6), 459 (3), 449 (8), 410 (2), 403 (2), 358 (5), 339 (2), 285 (1), 231 (4), 214 (2), 130 (7), 101 (3).

Figure S8: $^{31}P\{^1H\}$ NMR reaction monitoring of the reduction of **2Dmp**.

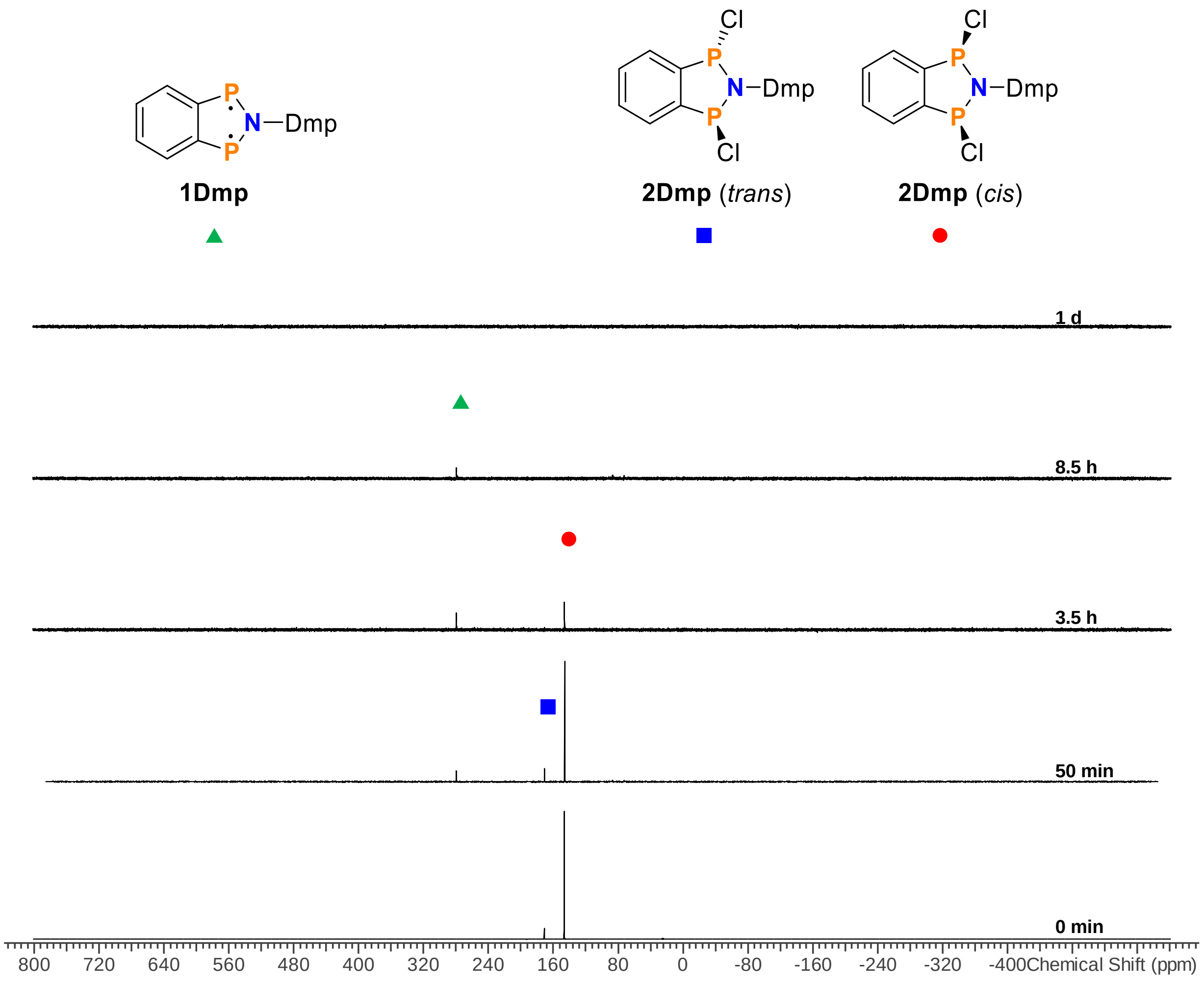

Figure S9: IR and Raman spectrum of the reaction product of the reduction of **2Dmp**.

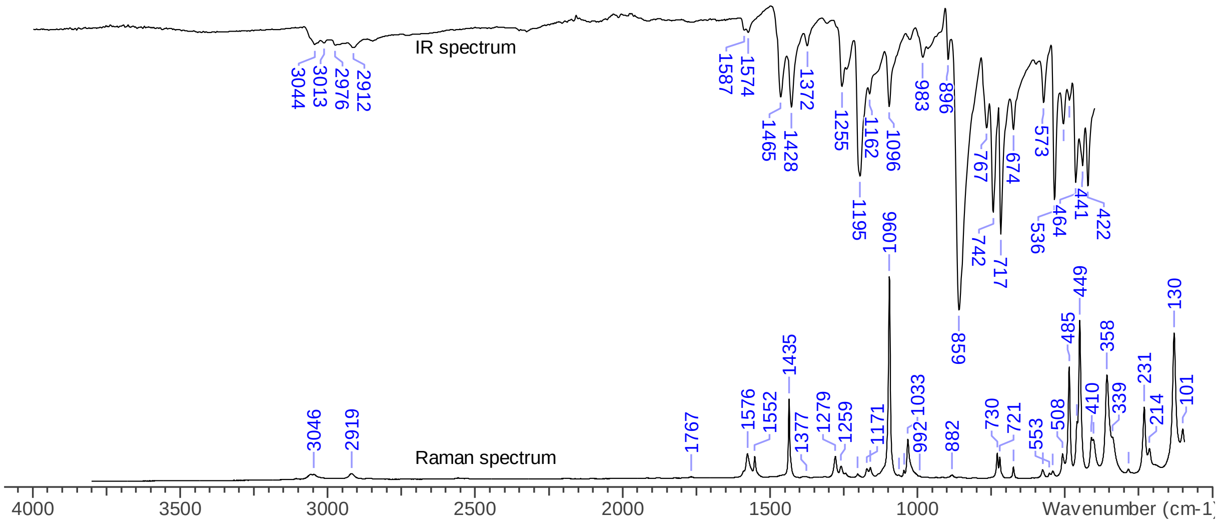

4.5 Synthesis of 3Ter

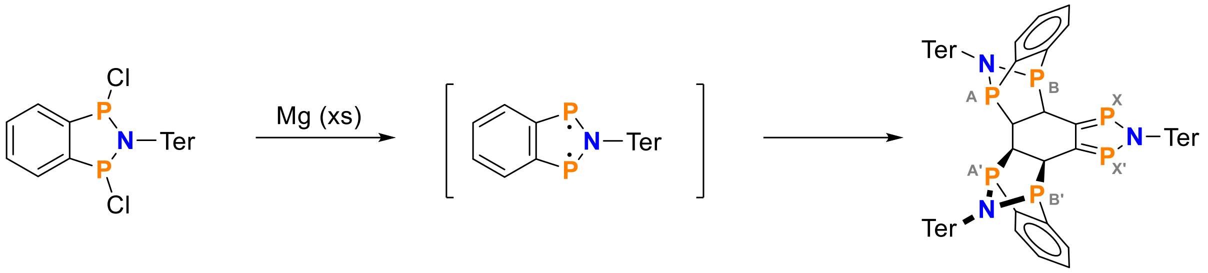

Mechanically activated magnesium chips (*cf.* Table S1; 863 mg, 35.5 mmol) and **2Ter** (658 mg, 1.23 mmol) are combined in a Schlenk flask equipped with a glass covered magnetic stir bar. *Attention: It is paramount to ensure that no grease finds its way into the reaction vessel. Otherwise the reduction is not reproducible.* THF (12 mL) is added while stirring. The reaction solution turns orange during a few minutes and is stirred for 40 h. As an intermediate, the biradical **1Ter** can be observed by $^{31}P\{^1H\}$ NMR spectroscopy. All volatiles are removed *in vacuo* (1×10^{-3} mbar) and the residue is dried *in vacuo* (1×10^{-3} mbar) at 40 °C (water bath) for two hours. The residue is extracted with benzene (15 mL) and the insoluble solids are filtered off. The intensively orange filtrate is concentrated to incipient crystallization. Storage at 0 °C overnight yields colorless crystals. The supernatant is removed by syringe and can be used for crystallization of further fractions. The colorless crystals are washed with *n*-pentane and dried *in vacuo* (1×10^{-3} mbar). Yield: 184 mg (0.132 mmol, 32%).

Single crystals of **3Ter** · 4 C_6H_6 suitable for SC-XRD were obtained as described above.

Intermediate:

$^{31}P\{^1H\}$ NMR (298 K, C_6D_6, 121.5 MHz): δ = 284.8 ppm (s).

Product:

Mp. 215 °C (Dec.). CHN calc. (found) in %: C 77.40 (72.21), H 6.28 (5.82), N 3.01 (2.40); deviations probably due to incomplete combustion, repeated measurements with and

without oxidizing agents did not result in better agreement (single crystals were used for EA). $^{31}P\{^1H\}$ NMR (298 K, CD_2Cl_2, 121.5 MHz): δ = 82.3 (m, P_A), 89.1 (m, P_B), 287.3 ppm (m, P_X); for assignment of the P atoms see reaction scheme above. $^1H\{^{31}P\}$ NMR (298 K, C_6D_6, 250.1 MHz): δ = 1.39 (m, 4 H, C*H* (C_6H_4)), 1.80 (br s, 12 H, C*H*$_3$), 2.00-2.40 (42 H, C*H*$_3$), 5.80-7.20 ppm (29 H, aromatic *H*). $^{13}C\{^1H\}$ NMR (298 K, C_6D_6, 62.9 MHz): δ = 20.6-23.6 (*C*H$_3$), 33.3-34.8 (*C*H), 119.0-131.1 (aromatic *C*H), 136.8-152.2 ppm (quaternary *C*). IR (ATR, 32 scans, cm^{-1}): = 3084 (vw), 3059 (w), 3026 (w), 2958 (w), 2929 (w), 2902 (w), 2867 (w), 1599 (w), 1581 (vw), 1492 (w), 1476 (w), 1445 (m), 1412 (w), 1395 (w), 1362 (w), 1336 (vw), 1323 (vw), 1290 (w), 1255 (w), 1237 (w), 1204 (w), 1181 (w), 1113 (m), 1076 (w), 1051 (w), 1031 (w), 1002 (vw), 895 (m), 866 (w), 851 (w), 812 (vw), 752 (m), 699 (vs), 674 (w), 649 (w), 633 (w), 620 (w), 606 (m), 587 (m), 577 (m), 559 (m), 532 (vw), 495 (m), 480 (s), 453 (m), 443 (s), 423 (m). Raman (633 nm, 10 s, 20 scans, cm^{-1})[2]: = 3049 (2), 3011 (1), 2917 (5), 2854 (1), 2730 (1), 1612 (4), 1580 (4), 1562 (1), 1484 (1), 1429 (2), 1382 (3), 1376 (3), 1305 (8), 1284 (2), 1267 (1), 1245 (4), 1218 (1), 1186 (1), 1163 (1), 1104 (2), 1079 (3), 1018 (4), 1007 (2), 947 (1), 834 (1), 739 (1), 705 (1), 677 (1), 667 (10), 656 (1), 604 (1), 575 (9), 559 (6), 536 (2), 526 (8), 516 (2), 505 (2), 455 (1), 419 (1), 407 (3), 394 (1), 339 (2), 331 (2), 296 (1), 287 (1), 252 (3), 234 (3), 209 (1), 199 (2). MS (CI, pos., isobutane, m/z): 933, 826, 509, 495 (an assignment was not possible.).

[2] Intensities were determined after baseline correction.

Figure S10: $^{31}P\{^{1}H\}$ reaction monitoring of the reduction of **3Ter**.

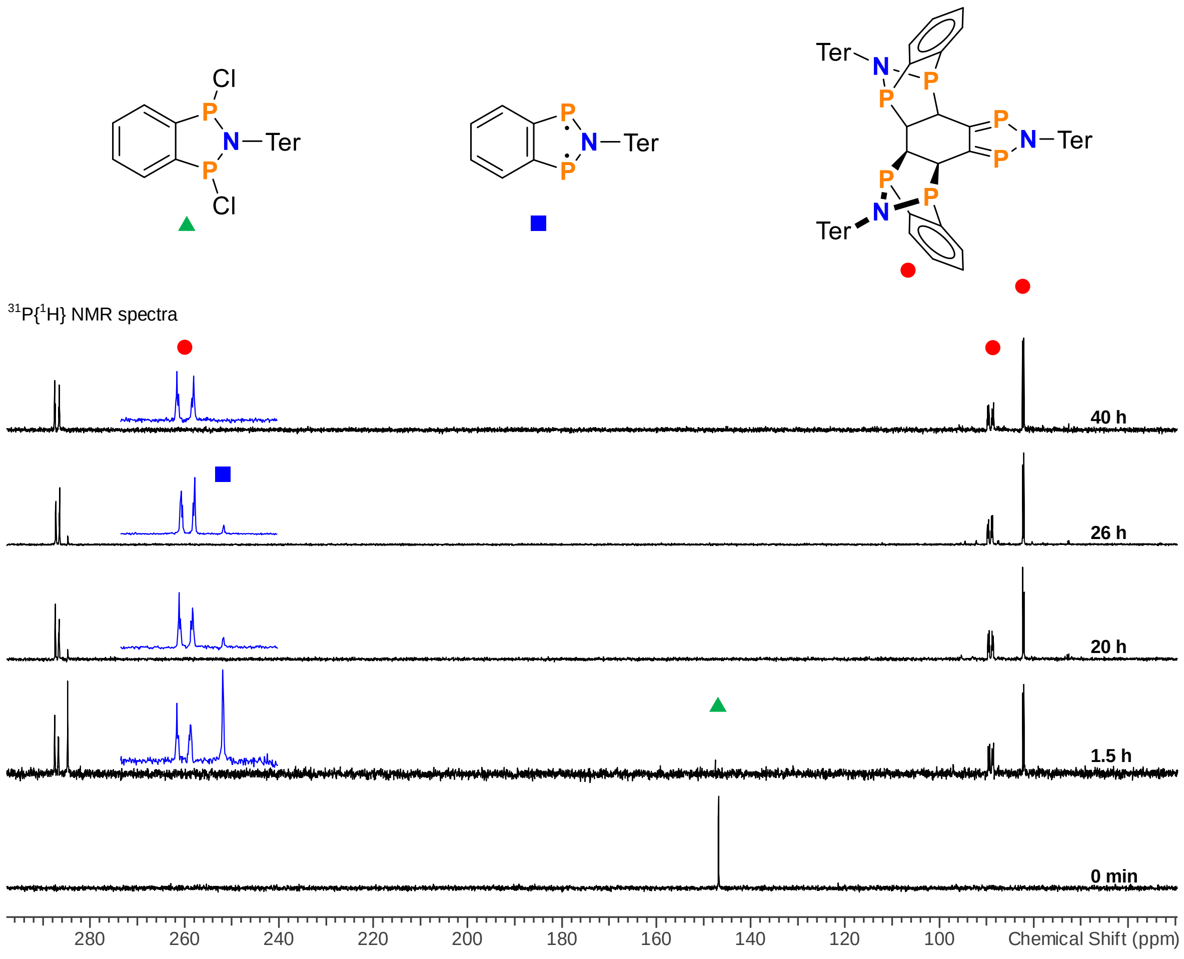

Figure S11: NMR, IR and Raman spectra of **3Ter** (solvent signals indicated by asterisks).

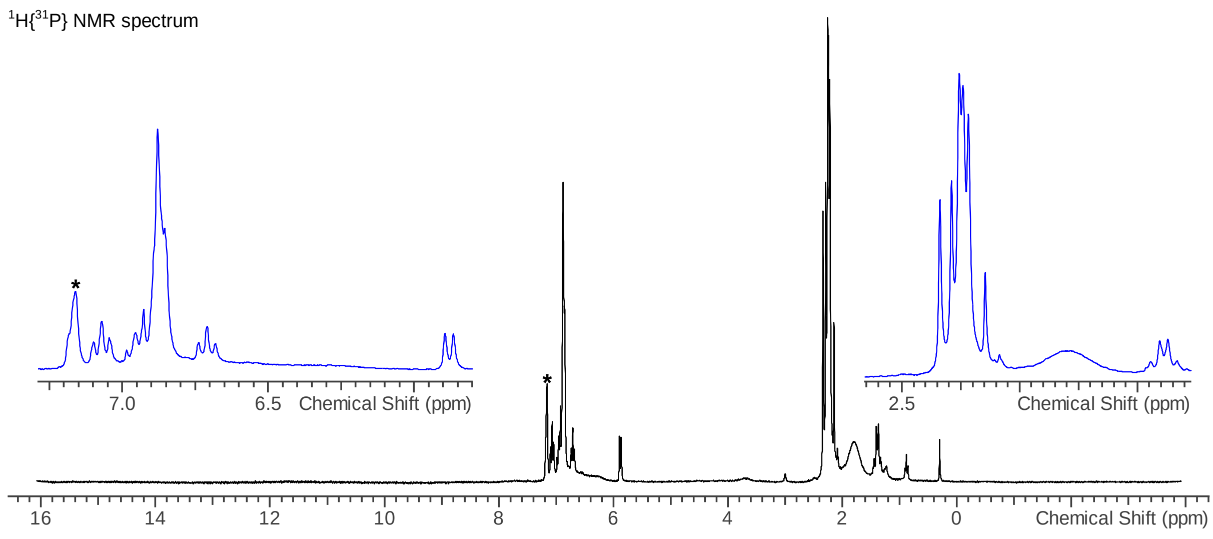

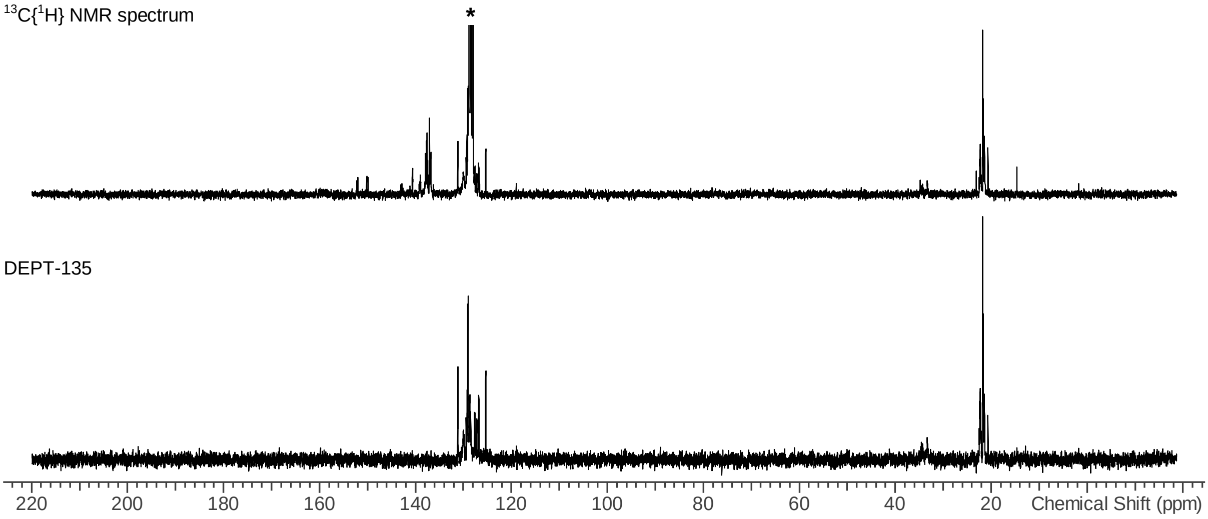
$^{13}C\{^1H\}$ NMR spectrum
*
DEPT-135
220
200
180
160
140
120
100
80
60
40
20
Chemical Shift (ppm)

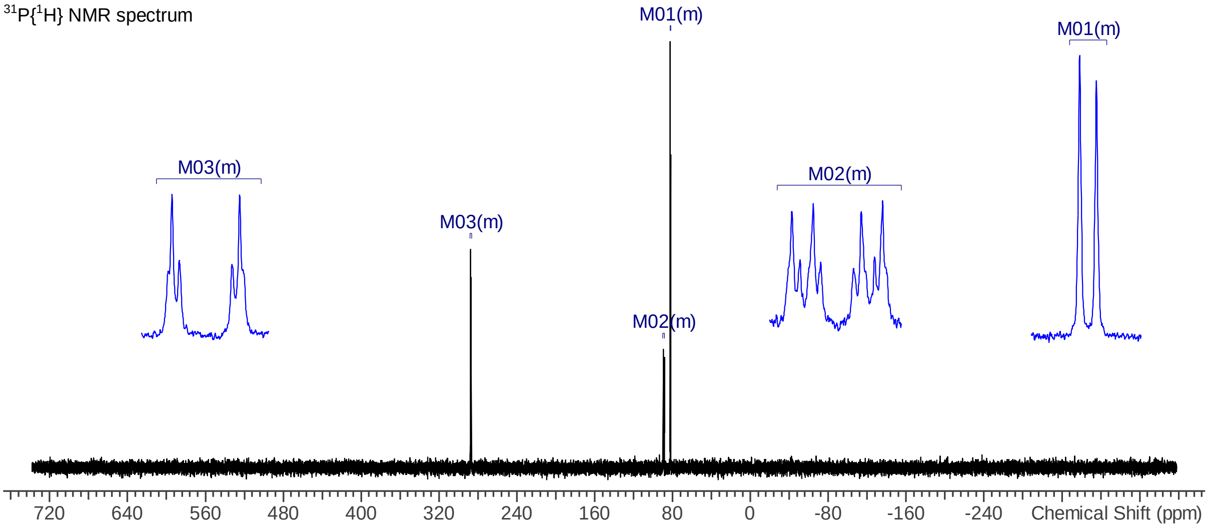
$^{31}P\{^1H\}$ NMR spectrum
M01(m)
M03(m)
M02(m)
M03(m)
M02(m)
M01(m)
720
640
560
480
400
320
240
160
80
0
-80
-160
-240
Chemical Shift (ppm)

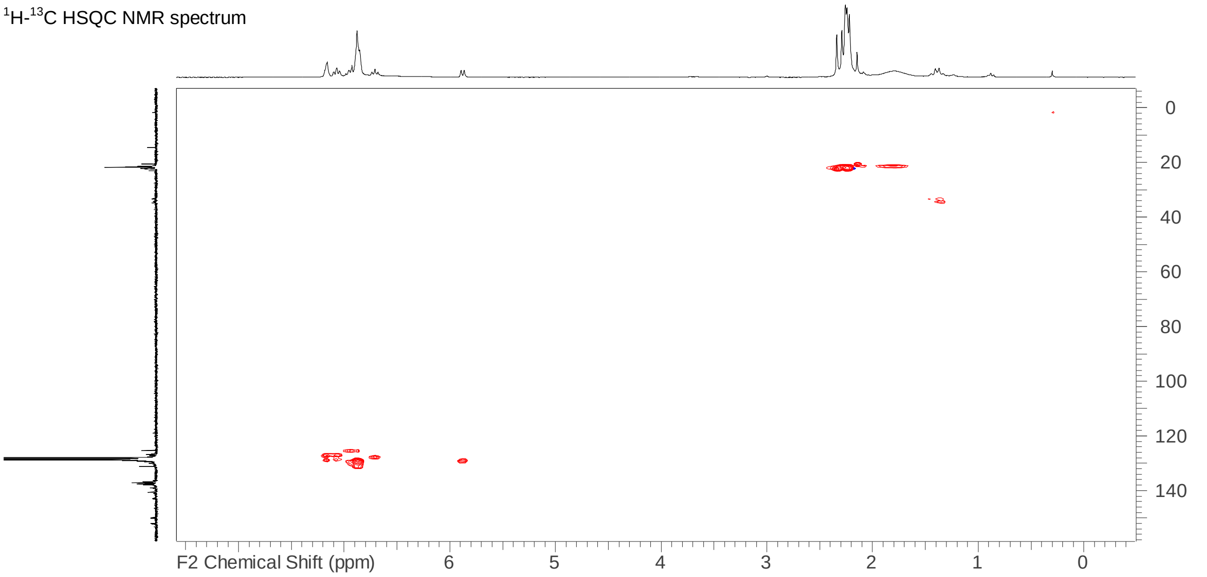
^1H-^{13}C HSQC NMR spectrum
0
20
40
60
80
100
120
140
F2 Chemical Shift (ppm)
6
5
4
3
2
1
0

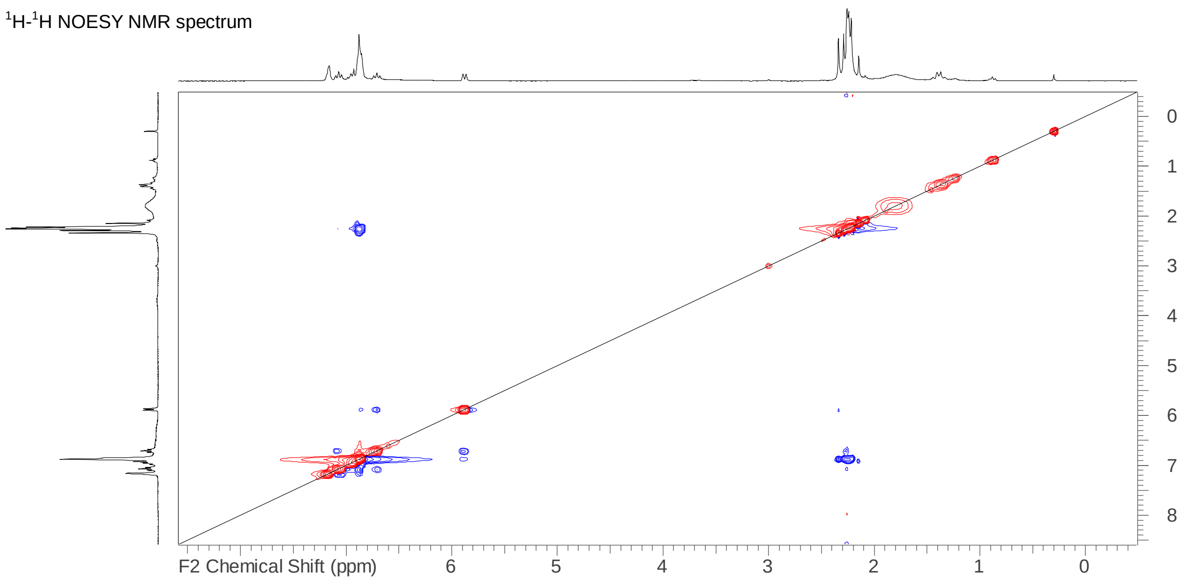

1H-1H NOESY NMR spectrum
F2 Chemical Shift (ppm)
6
5
4
3
2
1
0
8

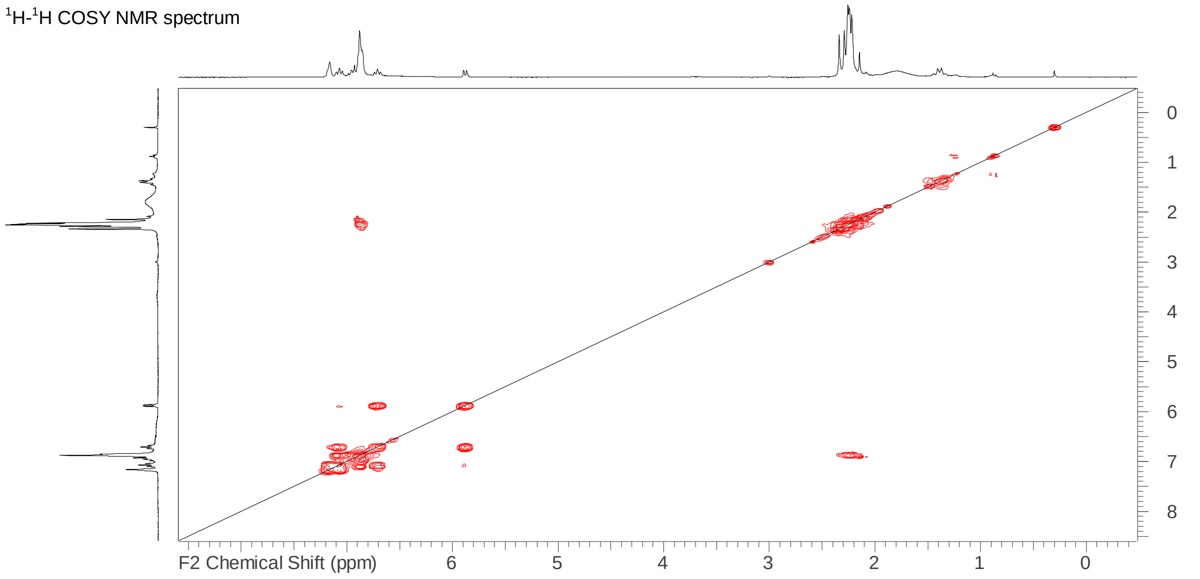

1H-1H COSY NMR spectrum
F2 Chemical Shift (ppm)
6
5
4
3
2
1
0
8

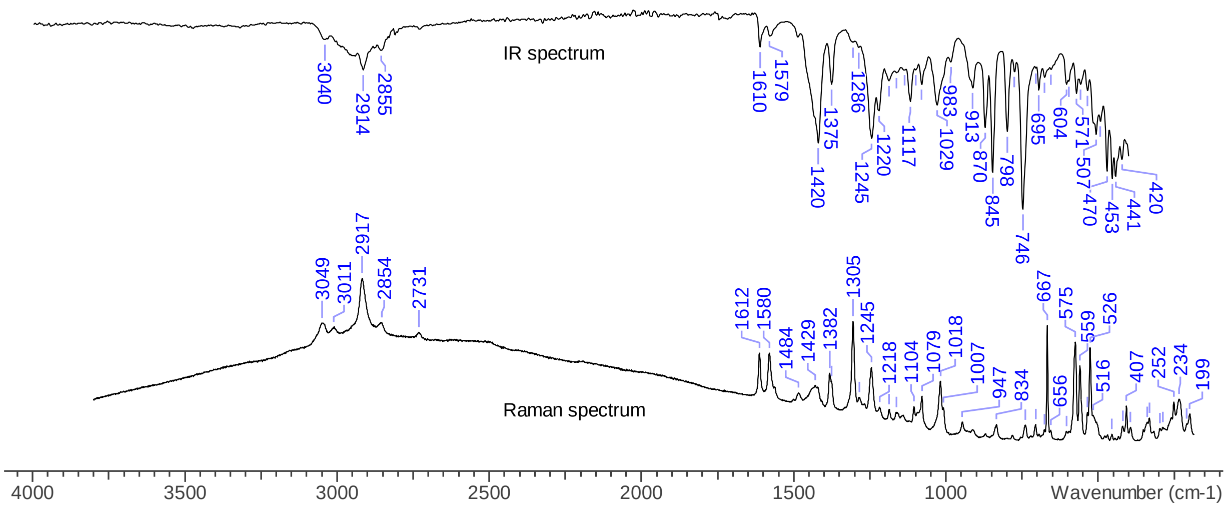

IR spectrum
3040
2914
2855
1610
1579
1420
1375
1286
1245
1220
1117
1029
983
913
870
845
798
746
695
604
571
507
470
453
441
420
Raman spectrum
3049
3011
2917
2854
2731
1612
1580
1484
1429
1382
1305
1245
1218
1104
1079
1018
1007
947
834
667
656
575
559
526
516
407
252
234
199
4000
3500
3000
2500
2000
1500
1000
Wavenumber (cm-1)

4.6 Synthesis of 1^{tBu}Bhp

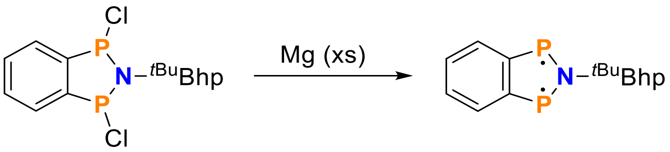

Mechanically activated magnesium chips (*cf.* Table S1; 320 mg, 11.1 mmol) and **2^{tBu}Bhp** (1.00 g, 1.46 mmol) are combined in a Schlenk flask equipped with a glass covered magnetic stir bar. *Attention: It is paramount to ensure that no grease finds its way into the reaction vessel. Otherwise the reduction is not reproducible.* THF (30 mL) is added while stirring. The reaction solution turns orange during a few minutes and is stirred for 24 h. The solvent is removed *in vacuo* (1×10^{-3} mbar) and the residue is dried *in* vacuo at 45 °C (water bath) for two hours. The residue is extracted with toluene (20 mL) and the insoluble solids are filtered off. The filtrate is concentrated to approx. 3 mL, yielding yellow crystals overnight. The supernatant is removed by syringe and can be used for crystallization of further fractions. The crystals are washed with *n-pentane* and dried *in vacuo* (1×10^{-3} mbar) at 60 °C (water bath) for two hours. Yield: 98 mg (0.16 mmol, 11%).

Single crystals of **1^{tBu}Bhp** suitable for SC-XRD were obtained as described above.

Mp. 252-258 °C. CHN calc. (found) in %: C 81.67 (79.57), H 6.04 (6.28), N 2.27 (2.19); deviations probably due to incomplete combustion, repeated measurements with and without oxidizing agents did not result in better agreement (single crystals were used for EA). ^{31}P{^{1}H} NMR (298 K, C_6D_6, 202.5 MHz): δ = 285.1 (s). ^{1}H NMR (298 K, C_6D_6, 500.1 MHz): δ = 1.11 (s, 9 H, C(C*H*$_3$)$_3$), 5.68 (s, 2 H, C*H*Ph$_2$), 6.83 (m, 2 H, C*H* (C_6H_4)), 6.95-7.03 (superimposed signals, 12 H, C*H* (*m*- and *p*-C*H* (Ph)), 7.08-7.12 (m,8 H, *o*-C*H* (Ph), 7.46 (s, 2 H, *m*-C*H* (^{tBu}Bhp)), 8.01 ppm (m, 2 H, C*H* (C_6H_4)). ^{13}C{^{1}H} NMR (298 K, C_6D_6, 125.8 MHz): δ = 31.5 (s, C(CH$_3$)$_3$), 35.2 (s, C(CH$_3$)$_3$), 52.2 (s, CHPh$_2$), 123.6 (t, $J(^{13}C,^{31}P)$ = 7 Hz, CH (C_6H_4)), 127.0 (s, *p*-CH (Ph)), 127.3 (s, *m*-CH (^{tBu}Bhp)), 128.0 (t, $J(^{13}C,^{31}P)$ = 15 Hz, CH (C_6H_4)), 129.0 (s, *m*-CH (Ph)), 130.0 (s, *o*-CH (Ph)), 139.5 (t, $^{2}J(^{13}C,^{31}P)$ = 11 Hz, *i-C*

($^{t\text{Bu}}$Bhp)),141.7 (s, *o-C* ($^{t\text{Bu}}$Bhp)), 145.1 (s, *i*-C (Ph)), 151.0 (s, *p-C* ($^{t\text{Bu}}$Bhp)), 163.8 (dd, $J(^{13}C,^{31}P)$ = 23 Hz, $J(^{13}C,^{31}P)$ = 27 Hz, quarternary *C* (C_6H_4)). IR (ATR, 32 scans, cm^{-1}): = 3049 (w), 3017 (w), 2953 (w), 1595 (w), 1488 (m), 1467 (w), 1440 (m), 1410 (w), 1391 (w), 1360 (w), 1260 (m), 1184 (w), 1112 (w), 1073 (w), 1027 (w), 1001 (w), 914 (w), 894 (w), 746 (m), 695 (vs), 674 (m), 649 (w), 635 (w), 621 (w), 604 (m), 584 (m), 575 (m), 495 (w), 471 (w), 456 (w), 438 (m). Raman (633 nm, 10 s, 20 scans, cm^{-1})[3]: = 121 (5), 145 (5), 185 (1), 222 (1), 237 (2), 253 (1), 265 (1), 297 (1), 434 (1), 455 (1), 463 (1), 476 (1), 491 (1), 515 (4), 567 (1), 594 (1), 619 (1), 619 (2), 633 (1), 643 (1), 683 (1), 698 (1), 711 (1), 741 (1), 751 (1), 771 (1), 814 (1), 836 (1), 861 (1), 899 (1), 922 (1), 951 (1), 1114 (11), 1131 (2), 1165 (2), 1121 (1), 1157 (1), 1171 (1), 1179 (1), 1237 (1), 1245 (1), 1261 (1), 1295 (1), 1315 (6), 1395 (1), 1412 (1), 1416 (1), 1429 (7), 1451 (1), 1461 (1), 1512 (2), 1585 (1), 1591 (1), 1611 (2), 2714 (1), 2782 (1), 2885 (1), 2914 (1), 2927 (1), 2953 (1), 2968 (1), 3111 (1), 3131 (1), 3148 (2), 3163 (3), 3161 (1), 3195 (1). MS (CI, pos., isobutane, m/z): 618 $[M+H]^+$, 482 $[^{t\text{Bu}}BhpNH_3]^+$. UV-Vis (benzene, 0.11 mmol/L, nm; absorbance given in brackets): λ_{max} = 347 (0.2), 407 (0.7), 427 (0.9).

Figure S12: NMR, IR and Raman and UV-Vis spectra of **1$^{t\text{Bu}}$Bhp** (solvent signals indicated by asterisks).

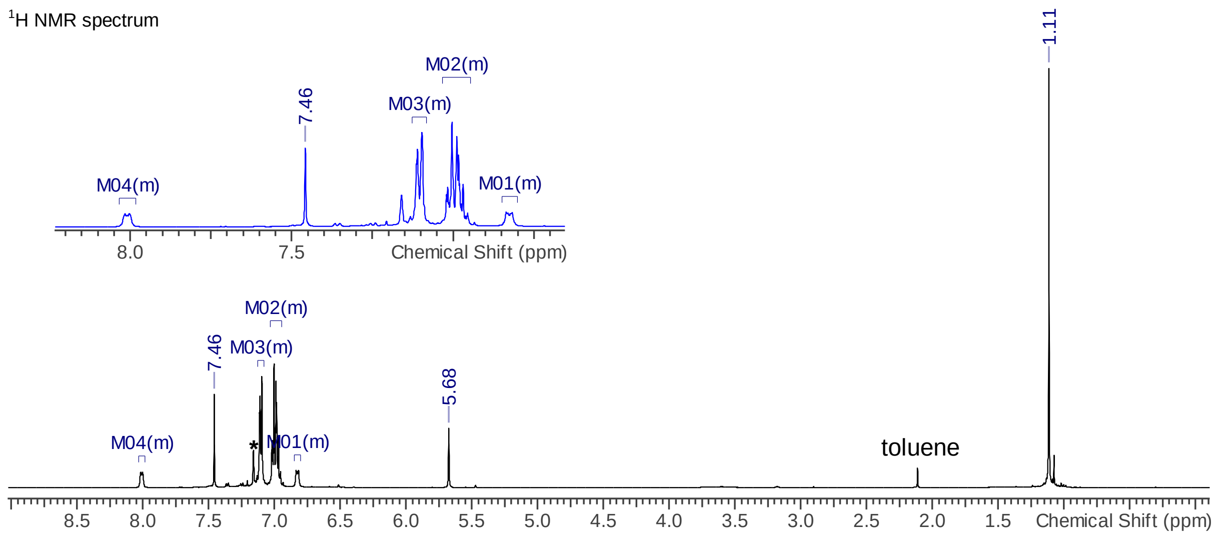

[3] Intensities were determined after baseline correction.

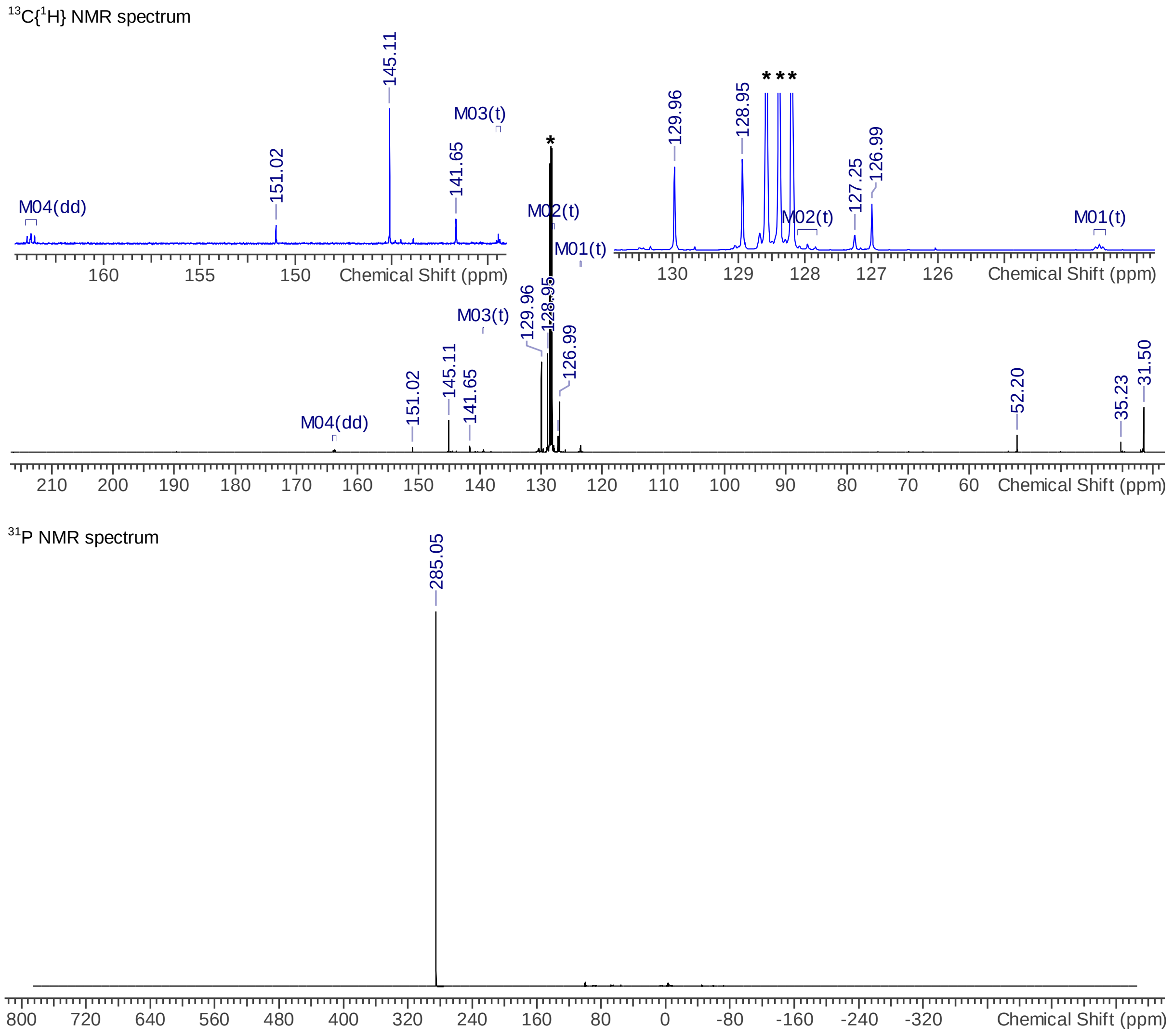
$^{13}C\{^1H\}$ NMR spectrum
145.11
M03(t)
151.02
141.65
M04(dd)
M02(t)
M01(t)
160
155
150
Chemical Shift (ppm)
129.96
128.95
127.25
126.99
M02(t)
M01(t)
130
129
128
127
126
Chemical Shift (ppm)
129.96
128.95
126.99
145.11
141.65
151.02
M04(dd)
52.20
35.23
31.50
210
200
190
180
170
160
150
140
130
120
110
100
90
80
70
60
Chemical Shift (ppm)
^{31}P NMR spectrum
285.05
800
720
640
560
480
400
320
240
160
80
0
-80
-160
-240
-320
Chemical Shift (ppm)

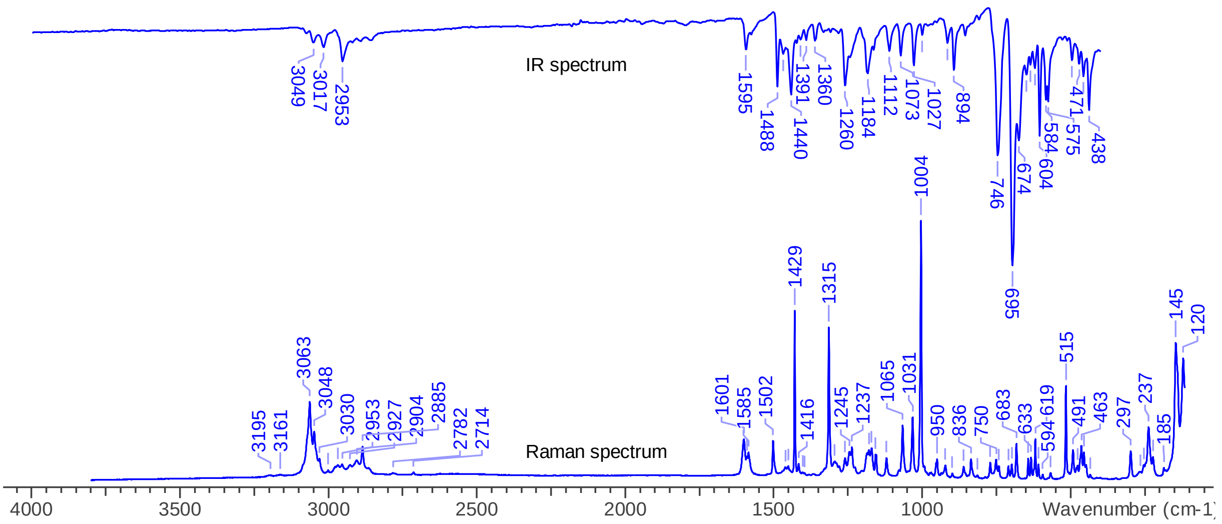
IR spectrum
3049
3017
2953
1595
1488
1391
1440
1360
1260
1184
1112
1073
1027
894
746
695
674
584
604
471
575
438
Raman spectrum
3195
3161
3063
3048
3030
2953
2927
2904
2885
2782
2714
1601
1585
1502
1429
1416
1315
1245
1237
1065
1031
1004
950
836
750
683
633
594
619
515
491
463
297
237
185
145
120
4000
3500
3000
2500
2000
1500
1000
Wavenumber (cm-1)

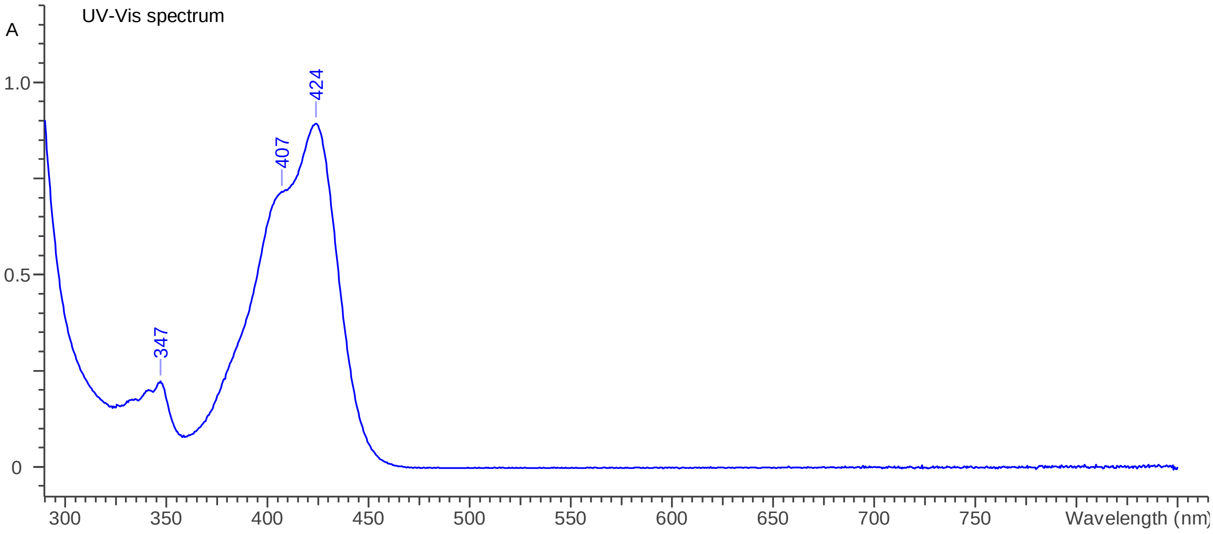

UV-Vis spectrum
A
1.0
0.5
0
347
407
424
300
350
400
450
500
550
600
650
700
750
Wavelength (nm)

5 Additional spectroscopic details

5.1 ^{31}P NMR data of 3Ter

The following table contains experimental ^{31}P NMR data of **3Ter** in comparison with calculated values (GIAO method, PBE-D3/def2-SVP; in brackets).

Table S3. Experimental ^{31}P NMR shifts and ^{31}P-^{31}P coupling constants of **3Ter**, calculated values in brackets (PBE-D3/def2-SVP). For the designation of the atoms see Figure S13.

X	*δ* [ppm]			*J* [Hz]		
		P_A-*X*	$P_{A'}$-*X*	P_B-*X*	$P_{B'}$-*X*	P_X-*X*
P_A	82.3<br>(78.9)					
$P_{A'}$	82.3<br>(78.9)	4.5<br>(4.5)				
P_B	89.1<br>(90.8)	−30.6<br>(−39.1)	−0.2<br>(−0.3)			
$P_{B'}$	89.1<br>(90.8)	−0.2<br>(−0.3)	−30.6<br>(−39.1)	−2.2<br>(−2.2)		
P_X	287.3<br>(285.3)	−1.0<br>(−1.7)	−0.9<br>(−0.4)	97.8<br>(83.2)	3.1<br>(0.2)	
$P_{X'}$	287.3<br>(285.3)	−0.9<br>−0.4	−1.0<br>(−1.7)	3.1<br>(0.2)	97.8<br>(83.2)	−17.7<br>(−10.3)

Figure S13: Experimental and simulated $^{31}P\{^{1}H\}$ NMR spectrum of **3Ter.**

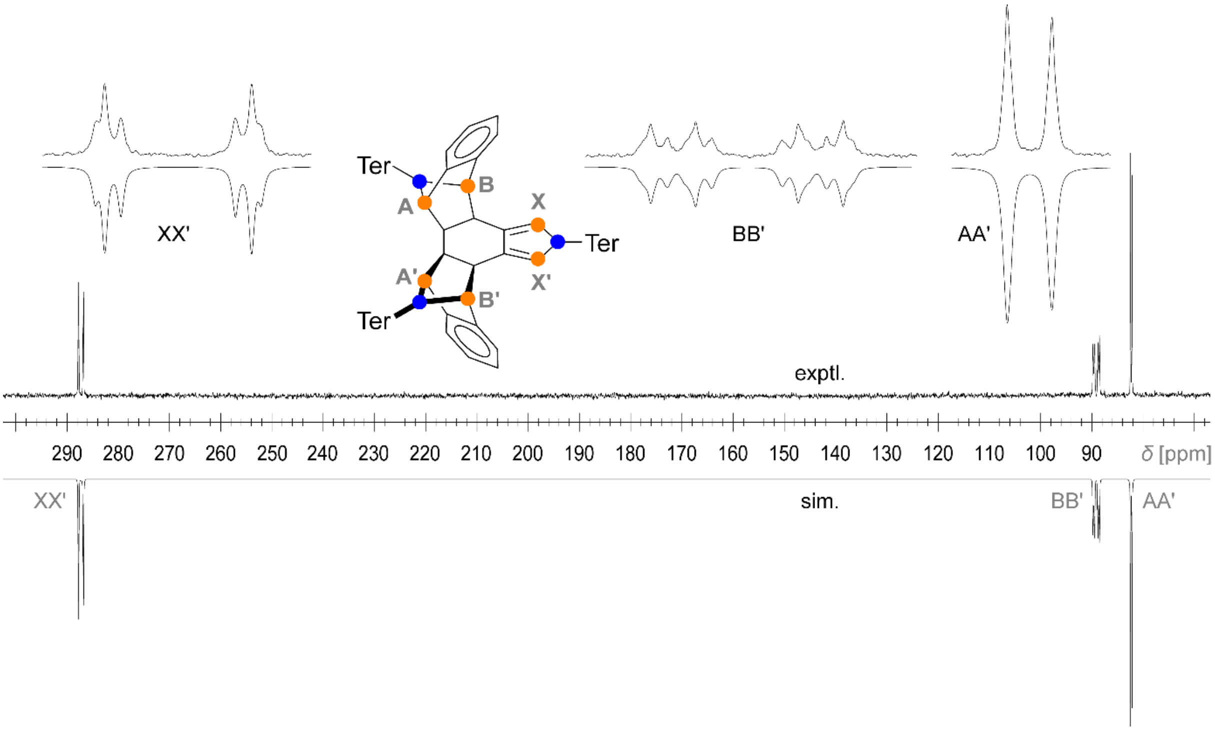

6 Computational details

6.1 General remarks

Computations were carried out using Gaussian09[12] or ORCA 4.2.1.[13]

Structure optimizations for **2Dmp**, **2Ter**, **2^{tBu}Bhp**, **benzene** and **indole** employed the hybrid DFT functional PBE0[14–16] in conjunction with Grimme's dispersion correction D3(BJ)[17,18] and the def2 basis set family[19] (notation PBE0-D3/def2-SVP or PBE0-D3/def2-TZVP).

Since biradicals require multi-determinantal wave functions, they cannot be accurately described by typical single-reference methods such as HF, DFT or coupled cluster theory. A qualitatively correct wave function can be obtained by multi-configurational SCF (MCSCF) methods which describe non-dynamic correlation, e.g. Complete Active Space SCF (CASSCF);[20–28] better descriptions including dynamic correlation require expensive multi-reference perturbation (MRPT) or multi-reference configuration-interaction (MRCI) calculations, which limit the size of the systems that can be computed. Nonetheless, previous investigations have shown that (especially non-hybrid) DFT methods can give reasonable results if the multi-configurational character is not too large.[29,30] Structure optimizations for biradicals **A**, **B**, **1^{tBu}Bhp** and **3Ter** were therefore carried out using the pure DFT exchange-correlation functional PBE,[14,15] which was previously shown to give reasonable results for biradicals **A** and **B**, especially regarding NMR data.[30] The PBE functional was used in conjunction with Grimme's dispersion correction D3(BJ)[17,18] and the def2-TZVP basis set[19] (notation PBE-D3/def2-TZVP). The resolution of identity (RI) approximation was employed, using the appropriate Coulomb fitting basis of the Ahlrichs group.[20] The stability of all Kohn-Sham wavefunctions was checked to verify the validity of the single-determinantal, restricted Kohn-Sham DFT approach. In accordance with CASSCF computations, which

indicate that the biradical character is not too large (vide infra), the restricted KS wavefunctions were stable in all instances with respect to symmetry breaking. All structures were fully optimized and confirmed as minima by frequency analyses. Chemical shifts and coupling constants were derived by the GIAO method.[31–35] The calculated absolute shifts ($\sigma_{calc,X}$) were referenced to the experimental absolute shift of 85% H_3PO_4 in the gas phase ($\sigma_{ref,1}$ = 328.35 ppm),[36] using PH_3 ($\sigma_{ref,2}$ = 594.45 ppm) as a secondary standard:[37]

$$\delta_{calc,X} = (\sigma_{ref,1} - \sigma_{ref,2}) - (\sigma_{calc,X} - \sigma_{calc,PH_3})$$
$$= \sigma_{calc,PH_3} - \sigma_{calc,X} - 266.1 \text{ ppm}$$

At the PBE-D3/def2-SVP level of theory, σ_{calc,PH_3} amounts to +617.22 ppm.

Please note that all computations were carried out for single, isolated molecules in the gas phase (ideal gas approximation). There may well be significant differences between gas phase and condensed phase.

6.2 Summary of calculated data

Table S4. Summary of calculated thermodynamic data (in a.u.).

Compound	PG	Opt. method	E_{tot}	U_{298}	H_{298}	G_{298}
**2Dmp** (*cis*)	C_s	PBE0-D3 def2-SVP	−2197.1328	−2196.8751	−2196.8742	−2196.9419
**2Dmp** (*trans*)	C_2		−2197.1309	−2196.8732	−2196.8722	−2196.9386
**2Ter** (*cis*)	C_s		−2815.3870	−2814.8408	−2814.8398	−2814.9451
**2Ter** (*trans*)	C_1		−2815.3793	−2814.8331	−2814.8322	−2814.9374
**2^{tBu}Bhp** (*cis*)	C_1		−3276.6333	−3275.9115	−3275.9105	−3276.0320
**2^{tBu}Bhp** (*trans*)	C_1		−3276.6271	−3275.9053	−3275.9044	−3276.0262
**A** ([P(μ-NTer)]$_2$)	D_2	PBE-D3 def2-TZVP	−2649.5088	−2648.6321	−2648.6312	−2648.7783
**B** (with Y=NDmp)	C_1		−3052.2911	−3051.2511	−3051.2502	−3051.4160
**1Dmp**	C_{2v}		−1278.0049	−1277.7622	−1277.7612	−1277.8213

Compound	PG	Opt. method	E_{tot}	U_{298}	H_{298}	G_{298}
**1Ter**	*C*		−1896.8174	−1896.2949	−1896.2939	-1896.3949
**1^{tBu}Bhp**	C_1		−2358.4956	−2357.8042	−2357.8033	−2357.9217
**3Ter**	C_2		−5690.5511	−5688.9759	−5688.9750	−5689.2155
**benzene**	D_{6h}	PBE0-D3 def2-TZVP	−232.0545			
**naphthalene**	D_{2h}		−385.5785			
**indole**	C_s		−363.5356			
**borazine**	D_{3h}		−242.4553			
**AH**	D_{2h}	PBE-D3 def2-TZVP	−793.0524			
**BH**	C_s		−886.4508			
**1H**	C_{2v}		−968.6068			

6.3 Comparison of the isomers of 2Dmp, 2Ter and 2^{tBu}Bhp

For **2Dmp**, **2Ter** and **2^{tBu}Bhp** two isomers are conceivable. In one, both Cl atoms are located on the same side of the central ring fragment (*cis* isomer), in the other, the Cl atoms are on opposite sides (*trans* isomer). Besides the *cis* isomer, the *trans* isomer is only observed in the ^{31}P NMR spectrum of **2Dmp**. The energetic difference between the isomers is shown in ***Table S5***. Values are calculated at the PBE0-D3/def2-SVP level of theory.

Table S5. Energetic comparison of the *cis* and *trans* isomers of **2Dmp**, **2Ter** and **2^{tBu}Bhp** (PBE0-D3/def2-SVP).

	**2Dmp**		**2Ter**		**2^{tBu}Bhp**	
	cis	*trans*	*cis*	*trans*	*cis*	*trans*
G_{298} (a.u.)	−2196.9419	−2196.9386	−2814.9451	−2814.9374	−3276.0320	−3276.0262
ΔG_r° (kJ/mol)	0	+8.8	0	+20.4	0	+15.2

6.4 Buried volumes and cone angles in 1Dmp, 1Ter and 1^{tBu}Bhp

The buried volumes caused by the different substituents in **1Dmp**, **1Ter** and **1^{tBu}Bhp** were calculated with the SambVca 2.1 application.[38] The structures used for the determination of the buried volume were optimized at the PBE-D3/def2-TZVP level of theory. For the calculations, the centre of the sphere was placed at the midpoint between the two P atoms. The value for the mesh spacing in the numerical integration was set to 0.01. The H atoms were included and the bond radii were scaled by 1.17. The $C_6H_4P_2N$ fragment in **1Dmp**, **1Ter** and **1^{tBu}Bhp** was removed for the calculations and therefore does not contribute to the buried volume

Table S6. Buried volumes[38] and cone angles[39]of the substituents in **1Dmp**, **1Ter** and **1^{tBu}Bhp**.

R	Dmp	Ter	^{tBu}Bhp
V_{bur} (r = 3.5 Å) [%]	27.5	36.2	41.3
V_{bur} (r = 6.0 Å) [%]	14.9	35.0	37.2
cone angle (d = 2.28 Å) [°]	161	212	217

Values of the cone angles were taken from data published by Schulz for a distance of 2.28 Å.[39] This is in agreement with the calculated distance between the centre of the P atoms and the *ipso*-C of the substituents (**1Dmp**: 2.28 Å, **1Ter**: 2.29 Å, **1^{tBu}Bhp**: 2.28 Å; PBE-D3/def2-TZVP). For the Dmp and the ^{tBu}Bhp substituent the Mes (2,4,6-trimethylphenyl) and ^{Me}Bhp (2,6-bis(diphenylmethyl)-4-methyl-phenyl) substituent were used for the determination of the cone angle. No significant deviation of the cone angle was assumed, because there are only variations in the substitution of the *para* position.

In the following the contour plots of the different substituents in **1Dmp**, **1Ter** and **1^{tBu}Bhp** are illustrated for sphere radii of 3.5 Å and 6.0 Å.

Figure S14: Orientation of the sterically demanding substituents in the contour plots in Table S7.

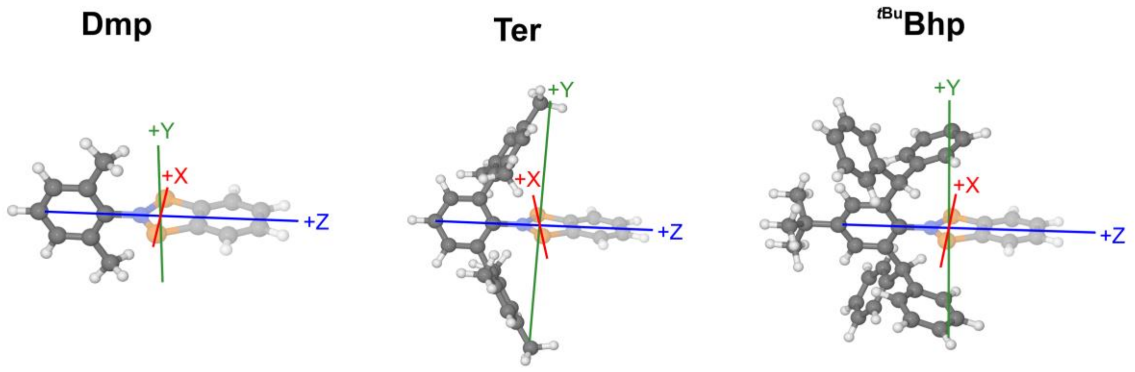

Table S7. Contour plots of the sterically demanding substituents in **1Dmp**, **1Ter** and **1^{tBu}Bhp** for radii of 3.5 Å and 6.0 Å.

	3.5 Å	6.0 Å
Dmp	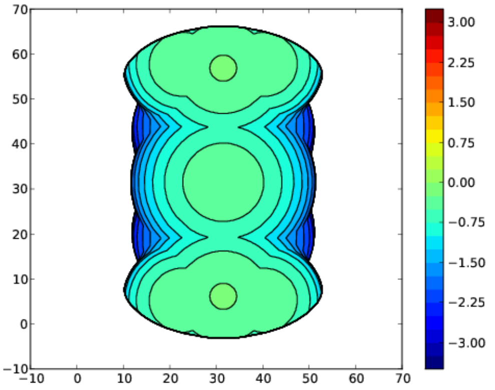	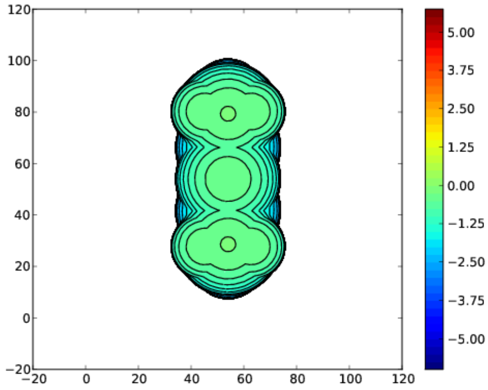
Ter	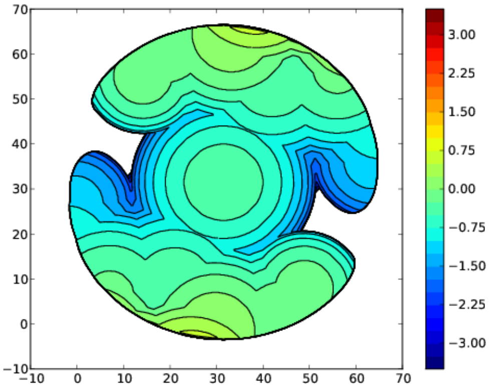	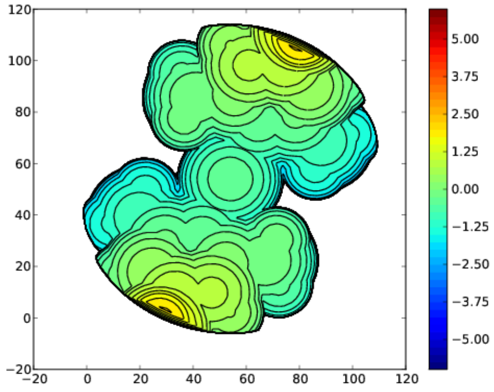

	3.5 Å	6.0 Å
***t*Bu**Bhp	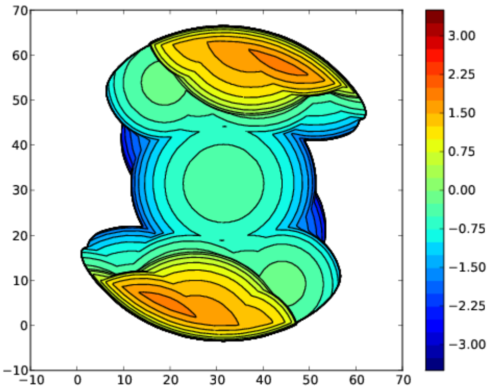	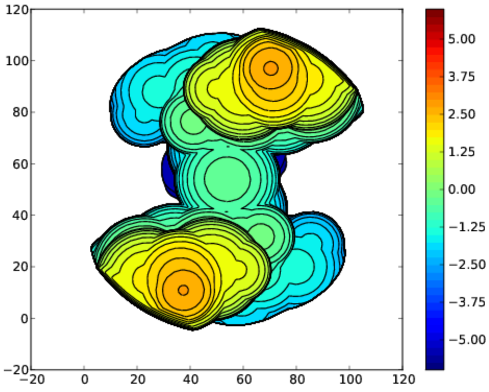

6.5 CASSCF computations of A, B, 1^{tBu}Bhp and 3Ter

The wave function of a biradical must be described by at least two determinants, since the frontier orbitals are nearly degenerate. In the following, the orbitals of the active space of **A**, **B**, **1^{tBu}Bhp** and **3Ter** are illustrated and their occupation numbers are given.

Table S8. Most important configurations that contribute to the CAS(2,2)/def2-TZVP wave function of **A** ($|c_i^2|$>0.01).

#	configuration	c_i^2
1	20	0.86
2	02	0.14

Table S9. Most important configurations that contribute to the CAS(6,4)/def2-TZVP wave function of **A** ($|c_i^2|$>0.01).

#	configuration	c_i^2
1	2220	0.87
2	2202	0.13

Figure S15: CAS(6,4)/def2-TZVP orbitals of **A**. The orbital occupation numbers of the CAS calculation are given.

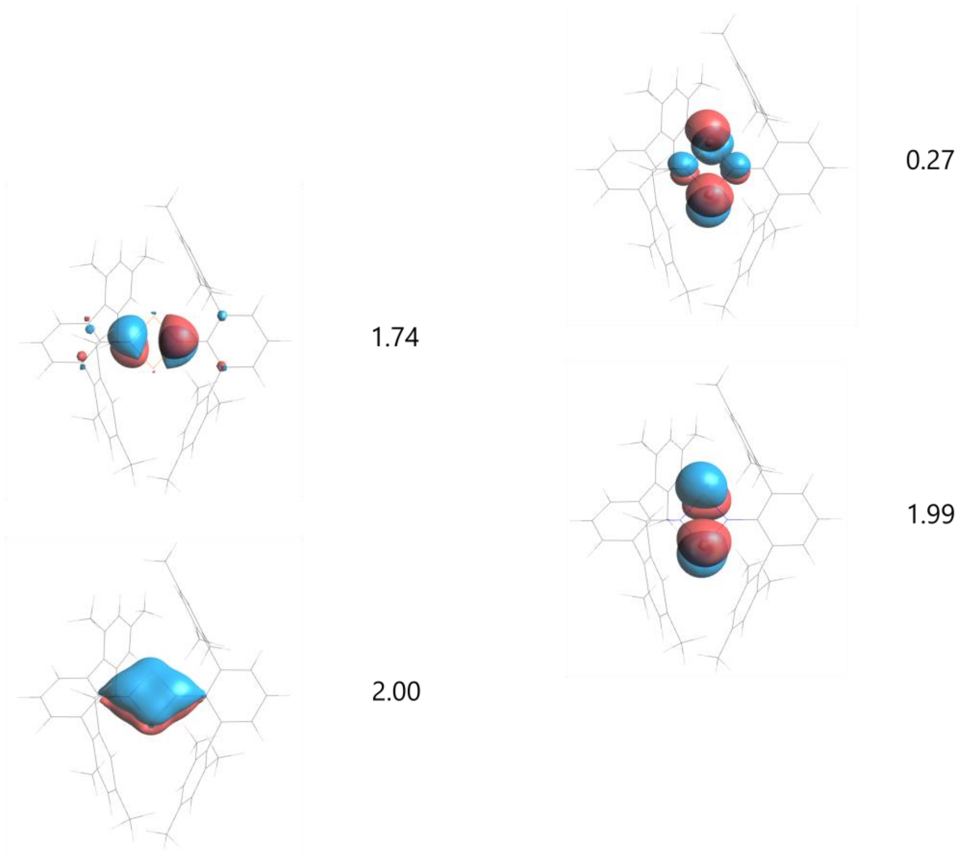

Table S10. Most important configurations that contribute to the CAS(2,2)/def2-TZVP wave function of **B** (with Y=NDmp) ($|c_i^2|>0.01$).

#	configuration	c_i^2
1	20	0.86
2	02	0.14

Table S11. Most important configurations that contribute to the CAS(8,6)/def2-TZVP wave function of **B** (with Y=NDmp) ($|c_i^2|>0.01$).

#	configuration	c_i^2
1	222200	0.83
2	222020	0.13
3	220202	0.02

Figure S16: CAS(8,6)/def2-TZVP orbitals of **B** (with Y=NDmp). The orbital occupation numbers of the CAS calculation are given.

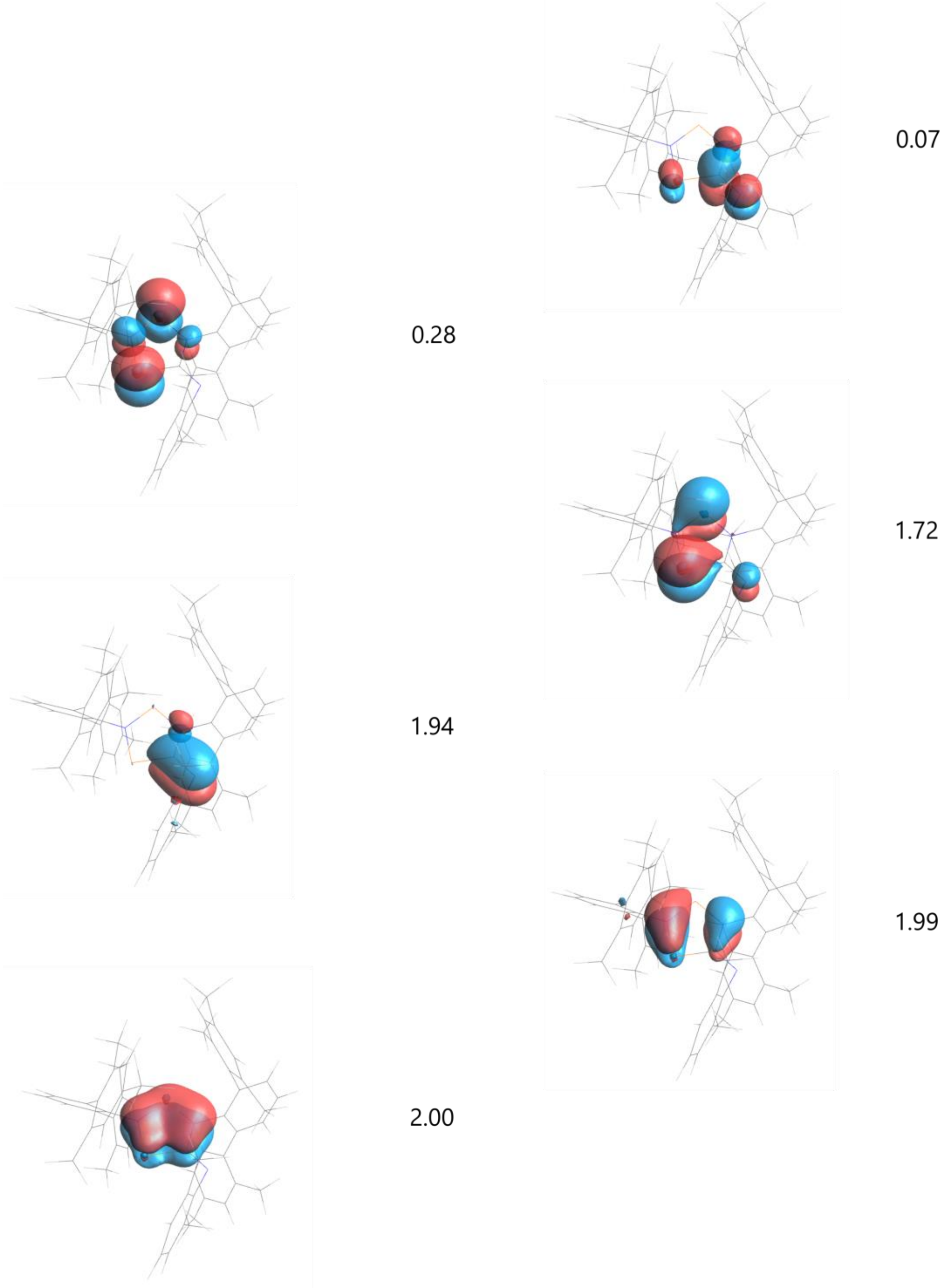

Table S12. Most important configurations that contribute to the CAS(2,2)/def2-TZVP wave function of **1^tBu^Bhp** ($|c_i^2|>0.01$).

#	configuration	c_i^2
1	20	0.91
2	02	0.09

Table S13. Most important configurations that contribute to the CAS(10,9)/def2-TZVP wave function of **1^tBu^Bhp** ($|c_i^2|>0.01$).

#	configuration	c_i^2
1	222220000	0.80
2	222202000	0.06
3	221211010	0.02
4	221120110	0.01
5	222111100	0.01

Figure S17: CAS(10,9)/def2-TZVP orbitals of **1^{tBu}Bhp**. The orbital occupation numbers of the CAS calculation are given.

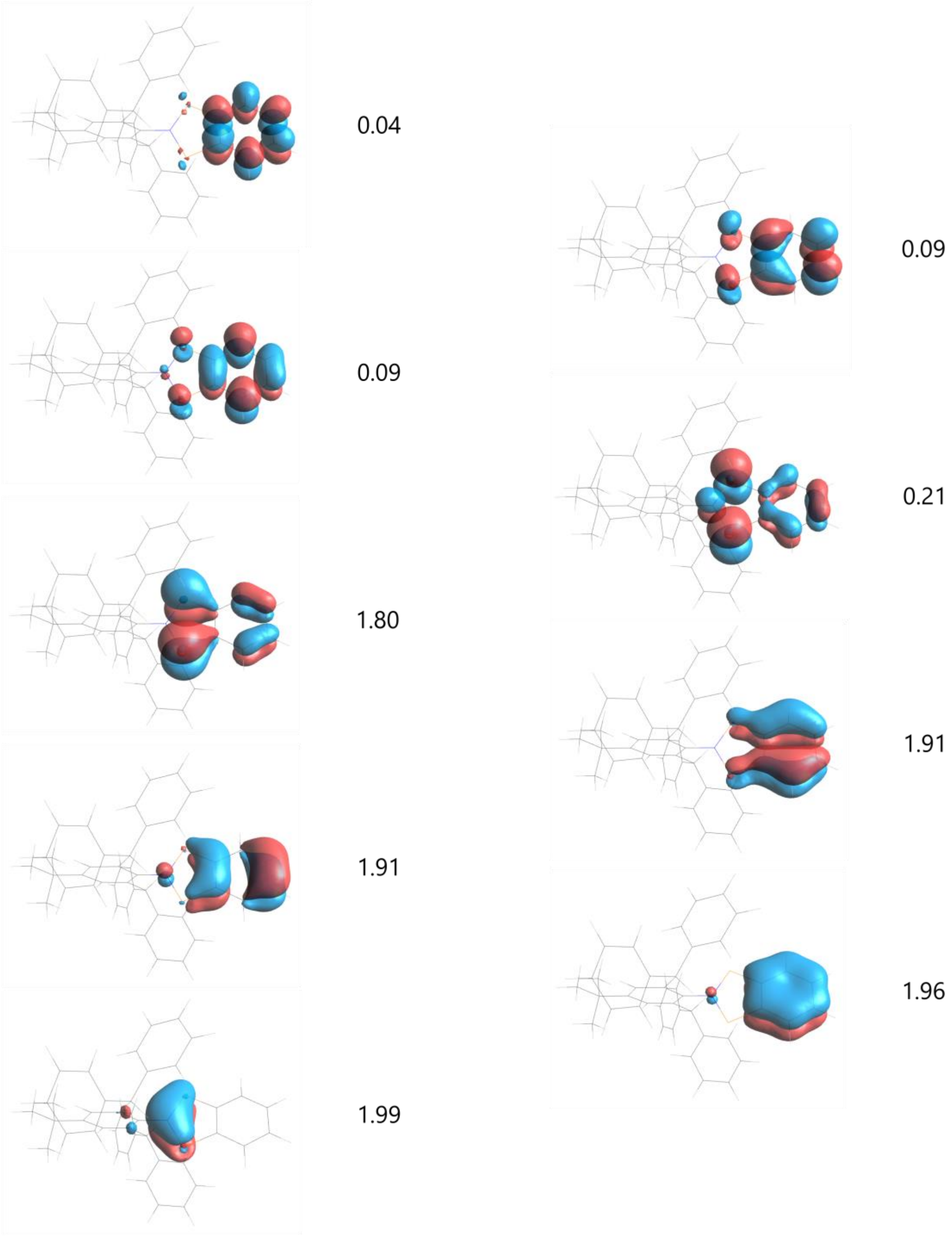

In case of **3Ter**, the CAS(6,4) results were approximated by a CAS(4,3) computation. In CAS(6,4), the composition of the active space was instable, as the lowest π-type orbital had an occupancy of nearly exactly 2 electrons (i.e. the results of CAS(6,4) and CAS(4,3) are identical for all intents and purposes).

Table S14. Most important configurations that contribute to the CAS(2,2)/def2-TZVP wave function of **3Ter** ($|c_i^2|>0.01$).

#	configuration	c_i^2
1	20	0.94
2	02	0.06

Table S15. Most important configurations that contribute to the CAS(4,3)/def2-TZVP wave function of **3Ter** ($|c_i^2|>0.01$).

#	configuration	c_i^2
1	220	0.94
2	202	0.05
3	022	0.01

Figure S18: CAS(4,3)/def2-TZVP orbitals of **3Ter**. The orbital occupation numbers of the CAS calculation are given.

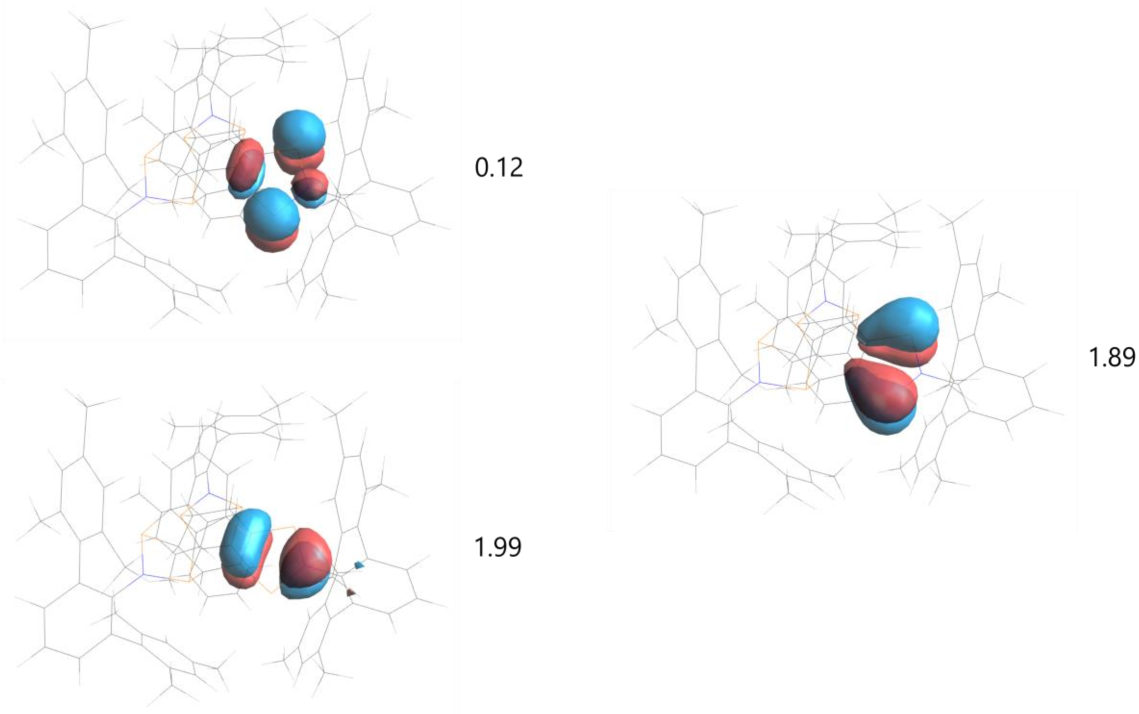

Table S16. Different parameters that describe the biradical character.[40–42] All calculations were performed with the def2-TZVP basis set.

		**A**	**B**[a]	**1^{tBu}Bhp**	**3Ter**
**CAS(2,2)**	**LUMO occ.**	0.28	0.28	0.18	0.12
	β[c]	0.28	0.28	0.18	0.12
**full π CAS**[b]	**LUMO occ.**	0.27	0.28	0.21	0.12
	β[c]	0.26	0.27	0.14	0.11
	**ΔE_{S-T}**[d] [kJ/mol]	−86.6	−57.6	−126.2	−170.6
**UHF NOs**	***y_0***[e]	0.30	0.40	0.23	0.10
	y_1[e]	0.00	0.01	0.01	0.00

[a] with Y = NDmp. [b] All π-type electrons of the central ring fragment were included in the active space (**A**: CAS(6,4), **B**: CAS(8,6), **1^{tBu}Bhp**: CAS(10,9), **3Ter**: CAS(6,4)). [c] $\beta = 2 \cdot c_2^2 / (c_1^2 + c_2^2)$.[40] [d] $\Delta E_{S-T} = E_S - E_T$. [e] $y_i = 1 - 2T_i / (1 + T_i^2)$, $T_i = (\eta_{HOMO-i} - \eta_{LUMO+i}) / 2$.[41,42]

6.6 Induced ring currents

To estimate the aromaticity of biradical **1**, the magnetically induced ring current density was computed using the gauge-including magnetically induced current (GIMIC) model,[43,44] as implemented in the GIMIC 2.1.4 code,[45,46] which was used in conjunction with the Gaussian09 interface.[47] To exclude any substituent effects on the ring current, a simple model system with R=H (i.e. **1H**) was employed. Nuclear shielding parameters were computed at the PBE-D3/def2-TZVP level of theory. Graphical representations of the current density were generated using ParaView's[48] streamline animation feature. Additionally, NICS(0) and NICS(1)$_{zz}$ values[49–51] were computed (PBE/def2-TZVP), which are often used as indicators for aromaticity. The calculated NICS(1)$_{zz}$ values nicely correspond to the results obtained using the GIMIC method.

For comparison, we additionally calculated benzene, naphthalene, and indole as reference molecules. The calculated values agree well with previously published results.[44,45,49,52,53] Moreover, [P(μ-NH)]$_2$ (**AH**) as a model for the established biradical [P(μ-NTer)]$_2$[54] was included in our computations.

Table S17. Integrated ring current susceptibilities and NICS values. For condensed ring systems, values are given for the five-membered (⬟) and six-membered part (⬢).

compound	ring current susceptibility [nA/T]			NICS(0)	NICS(1)$_{zz}$
	diatropic	paratropic	net induced		
**benzene**	17.1	−5.0	**12.1**	−8.2	**−30.2**
**naphthalene**	17.8	−4.7	**13.1**	−8.6	**−30.1**
**indole**	16.9 (⬟)<br>18.0 (⬢)	−4.8 (⬟)<br>−4.9 (⬢)	**12.1** (⬟)<br>**13.1** (⬢)	−12.6 (⬟)<br>−9.8 (⬢)	**−30.3** (⬟)<br>**−30.6** (⬢)
**borazine**	9.2	−6.0	**3.2**	−1.6	**−6.3**
**AH**	6.5	−4.4	**2.1**	−6.3	**−9.4**
**BH**	7.1	−3.7	**3.5**	−5.1	**−7.2**
**1H**	16.1 (⬟)<br>16.3 (⬢)	−2.6 (⬟)<br>−5.1 (⬢)	**13.5** (⬟)<br>**11.2** (⬢)	−11.7 (⬟)<br>−7.0 (⬢)	**−31.1** (⬟)<br>**−24.9** (⬢)

Figure S19: Streamline representations of the current densities of benzene (A), naphthalene (B), indole (C), borazine (D), **AH** (E), **BH** (F) and **1H** (G).

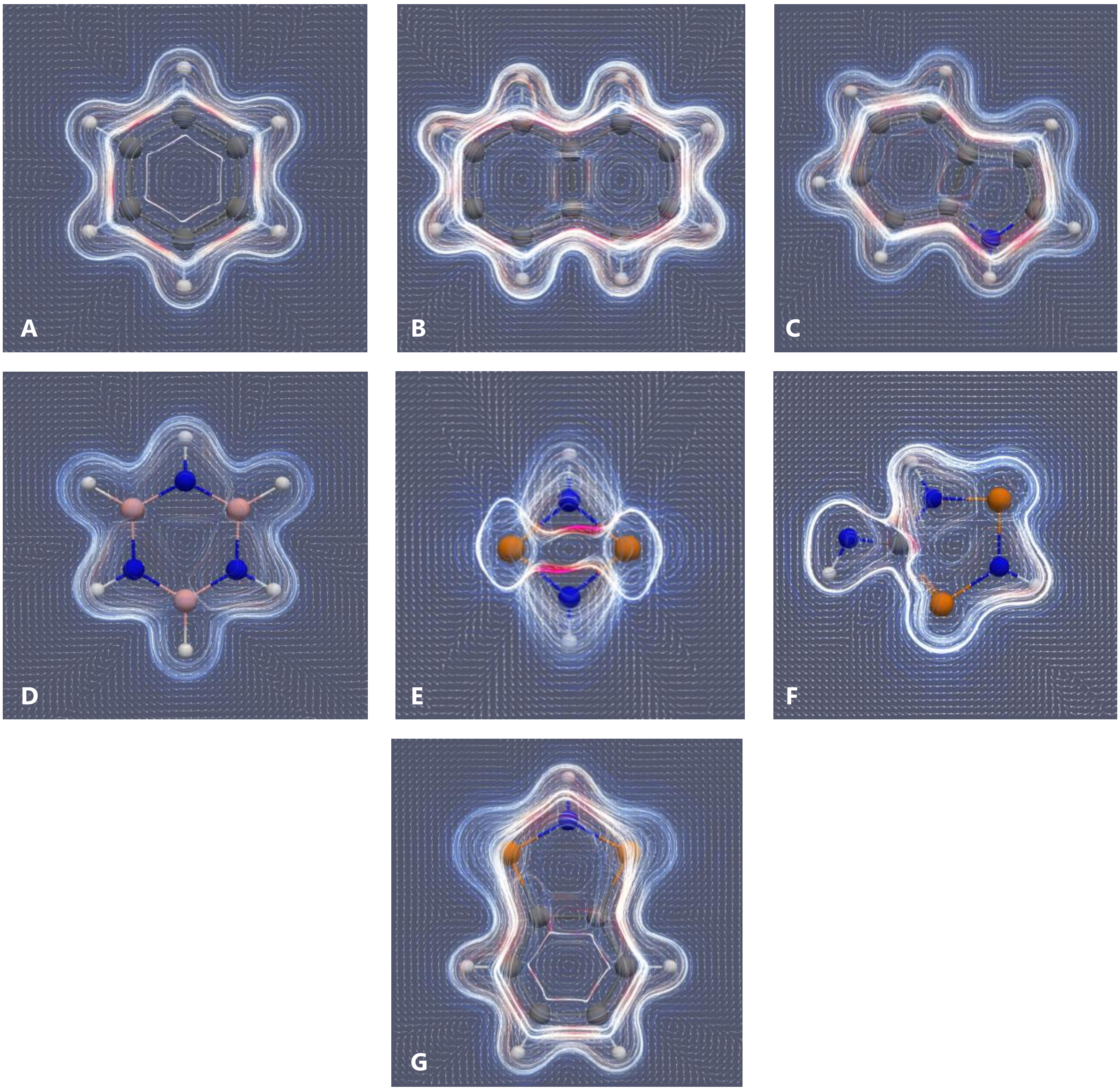

6.7 Comparison of optimized and crystal structures

Table S18. Selected experimental and calculated (PBE0-D3/def2-SVP) bond lengths in **2Dmp**. The MAE (mean absolute error) is 0.010 Å. The names of the atoms are taken from the crystal structure (*cf.* CIF file).

bond	calc. distance [Å]	exp. distance [Å]	absolute error [Å]
C6—P2	1.824	1.808	0.015
P2—Cl2	2.116	2.099	0.017
P2—N1	1.721	1.704	0.017
N1—P1	1.721	1.700	0.021
P1—Cl1	2.116	2.104	0.012
P1—C1	1.824	1.806	0.018
C1—C6	1.402	1.399	0.003
C1—C2	1.396	1.398	0.002
C2—C3	1.393	1.388	0.005
C3—C4	1.397	1.393	0.004
C4—C5	1.393	1.390	0.003
C5—C6	1.396	1.394	0.002

Table S19. Selected experimental and calculated (PBE0-D3/def2-SVP) angles in **2Dmp**. The MAE is 0.35°. The names of the atoms are taken from the crystal structure (*cf.* CIF file).

angle	calc. angle [°]	exp. angle [°]	absolute error [°]
C2—C1—C6	120.17	120.26	0.09
C2—C1—P1	123.75	123.99	0.24
C6—C1—P1	116.08	115.75	0.33
C3—C2—C1	119.46	119.23	0.23
C2—C3—C4	120.37	120.48	0.11
C5—C4—C3	120.37	120.54	0.17
C4—C5—C6	119.46	119.34	0.12
C5—C6—C1	120.17	120.13	0.04
C5—C6—P2	123.75	124.13	0.38
C1—C6—P2	116.08	115.73	0.35
P1—N1—P2	121.63	121.41	0.22
N1—P1—C1	92.89	93.61	0.72

angle	calc. angle [°]	exp. angle [°]	absolute error [°]
N1—P1—Cl1	106.24	105.79	0.45
C1—P1—Cl1	97.21	97.39	0.18
N1—P2—C6	92.89	93.44	0.55
N1—P2—Cl2	106.24	105.25	0.99
C6—P2—Cl2	97.21	98.08	0.87

Table S20. Selected experimental and calculated (PBE0-D3/def2-SVP) bond lengths in **2^{tBu}Bhp**. The MAE is 0.012 Å. The names of the atoms are taken from the crystal structure (*cf.* CIF file).

bond	calc. distance [Å]	exp. distance [Å]	absolute error [Å]
P1—N1	1.734	1.708	0.026
P1—Cl1	2.115	2.101	0.014
P1—C37	1.831	1.806	0.025
P2—N1	1.721	1.709	0.012
P2—C42	1.822	1.820	0.002
P2—Cl2	2.118	2.111	0.007
C37—C38	1.395	1.390	0.005
C37—C42	1.402	1.398	0.004
C38—C39	1.393	1.373	0.020
C39—C40	1.397	1.377	0.020
C40—C41	1.393	1.384	0.009
C41—C42	1.397	1.397	0.000

Table S21. Selected experimental and calculated (PBE0-D3/def2-SVP) angles in **2^{tBu}Bhp**. The MAE is 0.83°. The names of the atoms are taken from the crystal structure (*cf.* CIF file).

angle	calc. angle [°]	exp. angle [°]	absolute error [°]
N1—P1—C37	92.10	93.19	1.09
N1—P1—Cl1	104.97	104.60	0.37
C37—P1—Cl1	94.68	98.17	3.49
N1—P2—C42	93.00	92.69	0.31
N1—P2—Cl2	104.97	104.84	0.13
C42—P2—Cl2	98.41	94.14	4.27
P1—N1—P2	121.11	121.87	0.76
C38—C37—C42	120.07	119.90	0.17

angle	calc. angle [°]	exp. angle [°]	absolute error [°]
C38—C37—P1	123.57	124.10	0.53
C42—C37—P1	116.36	116.00	0.36
C39—C38—C37	119.50	119.70	0.20
C38—C39—C40	120.44	120.60	0.16
C39—C40—C41	120.26	120.90	0.64
C40—C41—C42	119.42	118.90	0.52
C41—C42—C37	120.30	119.80	0.50
C41—C42—P2	123.83	124.20	0.37
C37—C42—P2	115.84	116.00	0.16

Table S22. Selected experimental and calculated (PBE-D3/def2-TZVP) bond lengths in **3Ter**. The MAE is 0.014 Å. The names of the atoms are taken from the crystal structure (*cf*. CIF file).

bond	calc. distance [Å]	exp. distance [Å]	absolute error [Å]
P1—N1	1.722	1.698	0.024
P1—C6	1.738	1.724	0.014
P2—N1	1.722	1.698	0.024
P2—C1	1.738	1.719	0.019
P3—N2	1.743	1.727	0.016
P3—C55	1.845	1.834	0.011
P3—C2	1.904	1.879	0.025
P4—N2	1.738	1.715	0.023
P4—C60	1.837	1.827	0.010
P4—C3	1.909	1.893	0.016
P5—N3	1.738	1.725	0.013
P5—C85	1.837	1.831	0.006
P5—C4	1.909	1.902	0.007
P6—N3	1.743	1.719	0.024
P6—C90	1.845	1.829	0.016
P6—C5	1.904	1.880	0.024
C1—C6	1.404	1.397	0.007
C1—C2	1.498	1.509	0.011
C2—C3	1.552	1.559	0.007
C3—C4	1.545	1.554	0.009
C4—C5	1.552	1.558	0.006

bond	calc. distance [Å]	exp. distance [Å]	absolute error [Å]
C5—C6	1.498	1.505	0.007
C55—C56	1.394	1.383	0.011
C55—C60	1.410	1.404	0.006
C56—C57	1.400	1.383	0.017
C57—C58	1.396	1.374	0.022
C58—C59	1.399	1.399	0.000
C59—C60	1.393	1.381	0.012
C85—C86	1.393	1.382	0.011
C85—C90	1.410	1.408	0.002
C86—C87	1.399	1.376	0.023
C87—C88	1.396	1.381	0.015
C88—C89	1.400	1.377	0.023
C89—C90	1.394	1.390	0.004

Table S23. Selected experimental and calculated (PBE-D3/def2-TZVP) angles in **3Ter**. The MAE is 0.36°. The names of the atoms are taken from the crystal structure (*cf.* CIF file).

angle	calc. angle [°]	exp. angle [°]	absolute error [°]
N1—P1—C6	94.13	94.32	0.19
N1—P2—C1	94.13	94.43	0.30
N2—P3—C55	92.85	93.15	0.31
N2—P3—C2	92.79	92.65	0.14
C55—P3—C2	94.67	94.66	0.01
N2—P4—C60	93.27	93.47	0.20
N2—P4—C3	93.15	93.13	0.02
C60—P4—C3	93.51	93.64	0.13
N3—P5—C85	93.27	93.14	0.13
N3—P5—C4	93.15	93.30	0.15
C85—P5—C4	93.51	93.34	0.17
N3—P6—C90	92.85	93.25	0.41
N3—P6—C5	92.79	92.72	0.07
C90—P6—C5	94.67	94.81	0.14
P1—N1—P2	118.55	118.68	0.13
P4—N2—P3	107.91	108.31	0.40
P6—N3—P5	107.91	108.34	0.43

angle	calc. angle [°]	exp. angle [°]	absolute error [°]
C6—C1—C2	122.92	122.30	0.62
C6—C1—P2	116.59	116.30	0.29
C2—C1—P2	120.34	121.30	0.96
C1—C2—C3	118.01	118.50	0.50
C1—C2—P3	107.58	106.70	0.88
C3—C2—P3	108.78	108.60	0.18
C4—C3—C2	118.78	118.60	0.18
C4—C3—P4	108.87	109.30	0.43
C2—C3—P4	109.83	109.40	0.42
C3—C4—C5	118.78	118.50	0.28
C3—C4—P5	108.87	110.00	1.13
C5—C4—P5	109.83	109.10	0.73
C6—C5—C4	118.01	118.00	0.00
C6—C5—P6	107.58	107.20	0.38
C4—C5—P6	108.78	108.80	0.02
C1—C6—C5	122.92	123.70	0.78
C1—C6—P1	116.59	116.20	0.39
C5—C6—P1	120.34	119.90	0.44
C56—C55—C60	120.02	119.60	0.42
C56—C55—P3	127.15	127.60	0.45
C60—C55—P3	112.83	112.70	0.13
C55—C56—C57	119.33	119.80	0.47
C58—C57—C56	120.52	120.70	0.18
C57—C58—C59	120.43	120.30	0.13
C60—C59—C58	119.19	119.20	0.02
C59—C60—C55	120.50	120.40	0.10
C59—C60—P4	127.48	127.90	0.42
C55—C60—P4	112.00	111.70	0.30
C86—C85—C90	120.50	120.20	0.30
C86—C85—P5	127.48	128.30	0.82
C90—C85—P5	112.00	111.30	0.70
C87—C86—C85	119.19	119.50	0.31
C86—C87—C88	120.43	120.80	0.38
C89—C88—C87	120.52	120.30	0.22
C88—C89—C90	119.33	120.00	0.67
C89—C90—C85	120.02	119.10	0.92

angle	calc. angle [°]	exp. angle [°]	absolute error [°]
C89—C90—P6	127.15	127.70	0.55
C85—C90—P6	112.83	113.10	0.27
C86—C87—C88	120.43	120.80	0.38

Table S24. Selected experimental and calculated (PBE-D3/def2-TZVP) bond lengths in **1^{tBu}Bhp**. The MAE is 0.013 Å. The names of the atoms are taken from the crystal structure (*cf.* CIF file).

bond	calc. distance [Å]	exp. distance [Å]	absolute error [Å]
C42—P1	1.751	1.742	0.009
P1—N1	1.715	1.696	0.019
N1—P2	1.715	1.692	0.024
P2—C37	1.751	1.743	0.008
C42—C37	1.441	1.428	0.013
C37—C38	1.421	1.425	0.004
C38—C39	1.378	1.365	0.013
C39—C40	1.420	1.407	0.013
C40—C41	1.378	1.357	0.021
C41—C42	1.421	1.426	0.005

Table S25. Selected experimental and calculated (PBE-D3/def2-TZVP) angles in **1^{tBu}Bhp**. The MAE is 0.29°. The names of the atoms are taken from the crystal structure (*cf.* CIF file).

angle	calc. angle [°]	exp. angle [°]	absolute error [°]
C42—P1—N1	93.11	93.00	0.10
P1—N1—P2	121.28	121.62	0.33
N1—P2—C37	93.11	93.31	0.20
P2—C37—C42	116.25	115.78	0.47
P2—C37—C38	124.59	124.88	0.29
C42—C37—C38	119.15	119.28	0.13
C37—C38—C39	120.17	119.76	0.40
C38—C39—C40	120.68	120.92	0.24
C39—C40—C41	120.67	121.16	0.49
C40—C41—C42	120.17	120.15	0.01
C41—C42—C37	119.16	118.72	0.44
C41—C42—P1	124.59	124.99	0.40

6.8 Optimized structures (.xyz-files)

6.8.1 PH_3

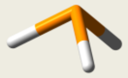

```
4
PH3 @ PBE-D3/def2-SVP
P          0.00000        0.00000        0.13345
H          0.00000        1.19835       -0.66725
H          1.03781       -0.59918       -0.66725
H         -1.03781       -0.59918       -0.66725
```

6.8.2 $C_6H_4P_2Cl_2N$-Dmp (2Dmp, *cis* isomer)

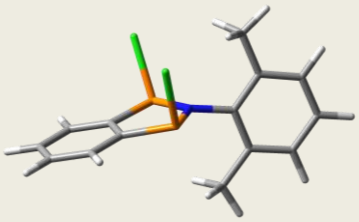

```
32
C6H4P2Cl2N-Dmp (cis isomer) @ PBE0-D3/def2-SVP
C          0.48850       -1.96978        0.70086
C          0.74663       -3.14909        1.40262
C          0.48850       -1.96978       -0.70086
P          0.15098       -0.36675        1.50262
C          1.00144       -4.32334        0.69851
H          0.73980       -3.15317        2.49571
C          1.00144       -4.32334       -0.69851
H          1.20342       -5.24891        1.24241
C          0.74663       -3.14909       -1.40262
H          1.20342       -5.24891       -1.24241
H          0.73980       -3.15317       -2.49571
P          0.15098       -0.36675       -1.50262
C          0.30653        1.89342        0.00000
C         -0.78741        2.77504        0.00000
C          1.63474        2.35738        0.00000
N          0.08659        0.47000        0.00000
C         -0.51891        4.14792        0.00000
C          0.78700        4.62661        0.00000
H         -1.35739        4.84886        0.00000
C          1.85631        3.73662        0.00000
H          0.97312        5.70323        0.00000
H          2.88284        4.11160        0.00000
Cl        -1.85530       -0.75943        2.04750
Cl        -1.85530       -0.75943       -2.04750
C          2.78206        1.39151        0.00000
H          2.75952        0.73635        0.88638
H          2.75952        0.73635       -0.88638
H          3.74239        1.92417        0.00000
C         -2.19876        2.27038        0.00000
H         -2.40003        1.64145       -0.87948
H         -2.40003        1.64145        0.87948
H         -2.90991        3.10708        0.00000
```

6.8.3 $C_6H_4P_2Cl_2N$-Dmp (2Dmp, *trans* isomer)

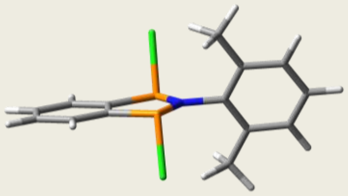

```
32
C6H4P2Cl2N-Dmp (trans isomer) @ PBE0-D3/def2-SVP
C          0.49653       0.49405      -2.08134
C          0.99725       0.98633      -3.28737
C         -0.49653      -0.49405      -2.08134
P          1.06024       1.07456      -0.44635
C          0.49636       0.49082      -4.48947
H          1.76787       1.76163      -3.28951
C         -0.49636      -0.49082      -4.48947
H          0.88238       0.87412      -5.43680
C         -0.99725      -0.98633      -3.28737
H         -0.88238      -0.87412      -5.43680
H         -1.76787      -1.76163      -3.28951
P         -1.06024      -1.07456      -0.44635
N          0.00000       0.00000       0.38659
Cl         0.00000       2.91584      -0.42684
Cl         0.00000      -2.91584      -0.42684
C          0.00000       0.00000       1.82315
C          1.11298      -0.52375       2.50896
C         -1.11298       0.52375       2.50896
C          1.09099      -0.50944       3.90655
C         -1.09099       0.50944       3.90655
C          0.00000       0.00000       4.60210
H          1.94709      -0.91416       4.45217
H         -1.94709       0.91416       4.45217
H          0.00000       0.00000       5.69465
C          2.29994      -1.07506       1.77581
H          1.99047      -1.70710       0.93151
H          2.92961      -0.26644       1.36929
H          2.92511      -1.67650       2.44977
C         -2.29994       1.07506       1.77581
H         -1.99047       1.70710       0.93151
H         -2.92961       0.26644       1.36929
H         -2.92511       1.67650       2.44977
```

6.8.4 $C_6H_4P_2Cl_2N$-Ter (2Ter, *cis* isomer)

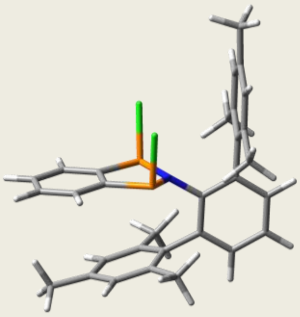

```
64
C6H4P2Cl2N-Ter (cis isomer) @ PBE0-D3/def2-SVP
C         -1.76014      -1.54411       0.70012
C         -2.55753      -2.44843       1.40247
C         -1.76014      -1.54411      -0.70012
P         -0.65012      -0.33207       1.49370
C         -3.35142      -3.35145       0.69857
H         -2.56040      -2.44807       2.49552
C         -3.35142      -3.35145      -0.69857
H         -3.97510      -4.06464       1.24266
C         -2.55753      -2.44843      -1.40247
H         -3.97510      -4.06464      -1.24266
H         -2.56040      -2.44807      -2.49552
P         -0.65012      -0.33207      -1.49370
C          1.48488       0.44554       0.00000
C          1.90643       1.78402       0.00000
C          2.42332      -0.60981       0.00000
N          0.08445       0.12305       0.00000
C          3.28039       2.05258       0.00000
```

C	4.21288	1.02004	0.00000
H	3.60899	3.09472	0.00000
C	3.78518	-0.30572	0.00000
H	5.28115	1.24914	0.00000
H	4.51060	-1.12261	0.00000
Cl	-2.09641	1.15206	1.86438
Cl	-2.09641	1.15206	-1.86438
C	1.91395	-2.00865	0.00000
C	1.62975	-2.65091	1.22124
C	1.62975	-2.65091	-1.22124
C	0.95740	-3.87599	1.19619
C	0.95740	-3.87599	-1.19619
C	0.58002	-4.48864	0.00000
H	0.71178	-4.36074	2.14585
H	0.71178	-4.36074	-2.14585
C	0.91617	2.89307	0.00000
C	0.45476	3.41451	-1.22027
C	0.45476	3.41451	1.22027
C	-0.49618	4.43572	-1.19776
C	-0.49618	4.43572	1.19776
C	-0.99362	4.95224	0.00000
H	-0.86662	4.83175	-2.14784
H	-0.86662	4.83175	2.14784
C	0.97299	2.88293	2.52503
H	2.06143	3.02573	2.61423
H	0.48773	3.37986	3.37588
H	0.79024	1.80076	2.62421
C	0.97299	2.88293	-2.52503
H	0.48773	3.37986	-3.37588
H	2.06143	3.02573	-2.61423
H	0.79024	1.80076	-2.62421
C	2.09027	-2.07714	-2.53238
H	3.12823	-2.38952	-2.73788
H	1.46476	-2.43131	-3.36364
H	2.07508	-0.97952	-2.53793
C	2.09027	-2.07714	2.53238
H	1.46476	-2.43131	3.36364
H	3.12823	-2.38952	2.73788
H	2.07508	-0.97952	2.53793
C	-2.06134	6.00721	0.00000
H	-1.99712	6.64857	-0.89111
H	-3.06318	5.54579	0.00000
H	-1.99712	6.64857	0.89111
C	-0.23117	-5.75027	0.00000
H	-0.03044	-6.36220	-0.89134
H	-0.03044	-6.36220	0.89134
H	-1.30746	-5.50914	0.00000

6.8.5 $C_6H_4P_2Cl_2N$-Ter (2Ter, *trans* isomer)

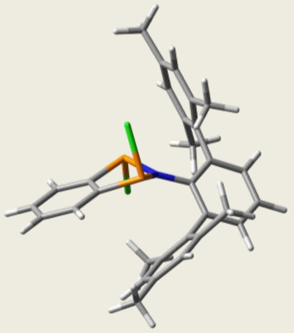

```
64
C6H4P2Cl2N-Ter (trans isomer) @ PBE0-D3/def2-SVP
P          1.08301        1.35482       -0.51480
Cl         1.06450        1.00487       -2.59291
N          0.12102        0.01502        0.11909
C         -0.38536        2.46640       -0.45359
P         -1.03930        0.49355        1.30238
C          0.30299       -1.34715       -0.24397
C         -0.51622        3.66052       -1.15983
C         -1.39759        2.05404        0.42167
Cl         0.23191        1.43529        2.76984
C          1.59808       -1.89940       -0.36521
H          0.26842        3.98562       -1.84806
C         -1.67875        4.41717       -1.00857
C         -2.55445        2.81302        0.57936
C          1.73829       -3.21485       -0.81525
C         -0.64539       -3.45949       -0.95290
H         -1.79806        5.34329       -1.57584
C         -2.69348        3.99575       -0.14728
H         -3.34611        2.47796        1.25434
H          2.74516       -3.62849       -0.90690
C          0.62753       -3.99371       -1.11954
H         -1.52999       -4.06298       -1.17098
H         -3.60226        4.59330       -0.04439
C         -0.83238       -2.14362       -0.51800
C         -2.22371       -1.64734       -0.30366
C         -2.92426       -2.07398        0.84874
C         -2.84525       -0.78139       -1.22222
C         -4.21393       -1.59968        1.07363
C         -4.13846       -0.32073       -0.94865
C         -4.83812       -0.71108        0.19121
H         -4.74230       -1.91721        1.97750
H         -4.61047        0.36451       -1.65903
C          2.78790       -1.09793        0.02109
C          2.97884       -0.79325        1.38826
C          3.70886       -0.64060       -0.93970
C          4.00870        0.07074        1.74825
C          4.73106        0.22343       -0.52966
C          4.87864        0.61776        0.79797
H          4.14140        0.32086        2.80487
H          5.42952        0.60378       -1.28106
H          0.75260       -5.01970       -1.47238
C          2.13211       -1.44357        2.44356
H          1.07158       -1.17461        2.35430
H          2.19472       -2.54052        2.36735
H          2.45773       -1.14325        3.44828
C          3.65378       -1.08087       -2.37495
H          4.28498       -1.97346       -2.52364
H          2.63678       -1.33833       -2.69393
H          4.03306       -0.29426       -3.04204
C         -2.26155       -2.95899        1.86208
H         -1.96513       -3.92872        1.43588
H         -1.33766       -2.48949        2.23985
H         -2.92409       -3.14503        2.71825
C         -2.14697       -0.32608       -2.46556
H         -1.45510       -1.08823       -2.84943
H         -2.87067       -0.07523       -3.25386
```

```
H          -1.54383        0.57373       -2.27159
C           5.93957        1.59646        1.20790
H           6.45128        1.27432        2.12754
H           6.69632        1.72697        0.42146
H           5.49856        2.58589        1.41340
C          -6.23301       -0.22235        0.45302
H          -6.97778       -0.98217        0.16282
H          -6.39077       -0.00793        1.52071
H          -6.45459        0.69067       -0.11782
```

6.8.6 $C_6H_4P_2Cl_2N$-$^{\mathit{t}Bu}$Bhp (2$^{\mathit{t}Bu}$Bhp, *cis* isomer)

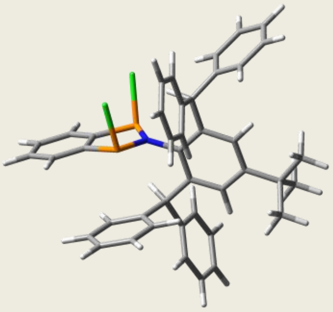

```
84
C6H4P2Cl2N-tBuBhp (cis isomer) @ PBE0-D3/def2-SVP
P           0.86880       -1.24301       -2.12351
Cl         -0.65289       -2.45220       -2.96482
N           0.36563       -0.95918       -0.50250
C           2.02144       -2.56682       -1.63523
P           0.76693       -2.06978        0.76791
C          -0.15249        0.34811       -0.21086
C           2.92561       -3.17253       -2.51047
C           1.99228       -2.93411       -0.28303
Cl         -0.68569       -3.60098        0.63408
C          -1.52622        0.56597       -0.05898
C           0.75317        1.42604       -0.10167
H           2.94869       -2.88723       -3.56554
C           3.79659       -4.14710       -2.02830
C           2.86493       -3.91183        0.19470
C          -1.97898        1.86571        0.19862
C          -2.51684       -0.58098       -0.10644
C           0.25801        2.69953        0.15119
C           2.24832        1.17215       -0.20082
H           4.50811       -4.62332       -2.70669
C           3.76529       -4.51487       -0.68090
H           2.84365       -4.19850        1.24909
H          -3.05293        2.01333        0.30293
C          -1.11222        2.94885        0.30672
H          -2.03194       -1.40203       -0.65987
C          -3.75652       -0.18939       -0.89048
C          -2.81718       -1.13652        1.27688
H           0.97143        3.52225        0.22289
H           2.40830        0.49062       -1.05404
C           2.76429        0.44797        1.02719
C           3.02883        2.42988       -0.53420
H           4.45354       -5.27817       -0.31029
C          -1.59412        4.37453        0.57164
C          -3.72709       -0.25512       -2.28794
C          -4.90993        0.29733       -0.26610
C          -3.73146       -2.19206        1.39836
C          -2.16149       -0.69033        2.42616
C           2.22869        0.68870        2.29805
C           3.80777       -0.47522        0.90261
C           3.78187        3.12439        0.41487
C           2.95771        2.93578       -1.83848
C          -3.11261        4.44358        0.73500
C          -0.93873        4.89686        1.85833
C          -1.18617        5.26386       -0.61234
H          -2.83398       -0.64331       -2.78481
```

```
C           -4.82039         0.16157        -3.04327
C           -6.00384         0.71894        -1.02101
H           -4.95410         0.33675         0.82516
H           -4.24435        -2.55819         0.50549
C           -3.98046        -2.78310         2.63064
C           -2.40627        -1.28633         3.66513
H           -1.44173         0.12774         2.35743
H            1.40574         1.39930         2.40634
C            2.71831         0.01567         3.41459
C            4.29333        -1.15595         2.01669
H            4.23151        -0.67630        -0.08505
H            3.85561         2.73632         1.43309
C            4.44119         4.30573         0.07108
C            3.61445         4.11274        -2.18330
H            2.36722         2.39870        -2.58652
H           -3.63781         4.09878        -0.16854
H           -3.41957         5.48369         0.92261
H           -3.45938         3.83555         1.58452
H           -1.21048         4.26768         2.72011
H           -1.27162         5.92583         2.06779
H            0.15873         4.91019         1.78232
H           -0.09564         5.27548        -0.75788
H           -1.51490         6.30174        -0.44367
H           -1.64588         4.90580        -1.54619
H           -4.78093         0.09773        -4.13354
C           -5.96252         0.65416        -2.41242
H           -6.89648         1.09672        -0.51609
H           -4.69270        -3.60890         2.70143
C           -3.31528        -2.33289         3.77304
H           -1.87583        -0.92611         4.55006
H            2.28244         0.20840         4.39768
C            3.74758        -0.91433         3.27654
H            5.09687        -1.88639         1.89606
H            5.02605         4.83693         0.82599
C            4.35839         4.80481        -1.22605
H            3.54787         4.49258        -3.20575
H           -6.82109         0.98054        -3.00429
H           -3.50585        -2.80108         4.74169
H            4.12282        -1.45228         4.15032
H            4.87566         5.72895        -1.49454
```

6.8.7 $C_6H_4P_2Cl_2N$-^{tBu}Bhp (2^{tBu}Bhp, *trans* isomer)

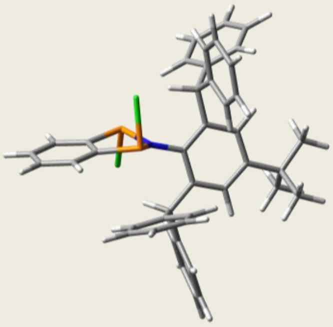

```
84
C6H4P2Cl2N-tBuBhp (trans isomer) @ PBE0-D3/def2-SVP
P           -0.32073         1.63444        -2.08275
Cl          -1.52563         0.26406        -3.15455
N           -0.36197         1.03727        -0.44670
C           -1.59578         2.89370        -1.72399
P           -0.83752         2.12749         0.80470
C            0.17013        -0.26217        -0.15738
C           -2.27370         3.63015        -2.69547
C           -1.84955         3.11201        -0.36384
Cl           0.76118         3.55493         0.83628
C            1.55020        -0.49017        -0.07970
C           -0.73517        -1.33172         0.02727
H           -2.08117         3.45507        -3.75712
C           -3.22214         4.57118        -2.29844
```

C	-2.79149	4.06133	0.02941
C	2.01025	-1.79021	0.16179
C	2.55494	0.64156	-0.17563
C	-0.23093	-2.60074	0.28641
C	-2.23380	-1.08083	-0.01144
H	-3.76866	5.14113	-3.05327
C	-3.47986	4.78565	-0.94279
H	-2.98933	4.23036	1.09095
H	3.08774	-1.94446	0.20169
C	1.14406	-2.86188	0.35173
H	2.03979	1.50296	-0.62985
C	3.69390	0.27045	-1.10852
C	3.00903	1.12469	1.19309
H	-0.94421	-3.41532	0.42306
H	-2.41382	-0.41044	-0.86412
C	-2.70275	-0.33214	1.21836
C	-3.02536	-2.34663	-0.28678
H	-4.22668	5.52419	-0.64215
C	1.62963	-4.28671	0.61340
C	3.51208	0.40372	-2.48946
C	4.90088	-0.25892	-0.64009
C	3.95921	2.15217	1.26626
C	2.45723	0.64390	2.38218
C	-2.15143	-0.56985	2.48262
C	-3.70744	0.63393	1.09442
C	-3.79675	-2.98359	0.68760
C	-2.95401	-2.91668	-1.56528
C	3.15500	-4.36886	0.67354
C	1.05952	-4.77506	1.95292
C	1.13226	-5.19527	-0.52082
H	2.57177	0.81817	-2.86482
C	4.50691	0.01226	-3.38153
C	5.89731	-0.65503	-1.53152
H	5.06555	-0.35066	0.43635
H	4.39174	2.54610	0.34308
C	4.34775	2.67997	2.49174
C	2.84411	1.17420	3.61410
H	1.70640	-0.14800	2.35019
H	-1.35818	-1.31406	2.58879
C	-2.58065	0.15378	3.59216
C	-4.13338	1.36442	2.20207
H	-4.14322	0.82784	0.11058
H	-3.87172	-2.54748	1.68607
C	-4.47457	-4.16890	0.39606
C	-3.62910	-4.09723	-1.85763
H	-2.34947	-2.42597	-2.33252
H	3.61960	-4.04815	-0.27119
H	3.46453	-5.40822	0.86111
H	3.56511	-3.74768	1.48455
H	1.39594	-4.13125	2.78043
H	1.39622	-5.80310	2.16083
H	-0.04059	-4.77785	1.95256
H	0.03434	-5.19615	-0.59200
H	1.46050	-6.23340	-0.35260
H	1.53100	-4.86214	-1.49121
H	4.34716	0.12720	-4.45645
C	5.70383	-0.52212	-2.90506
H	6.83399	-1.06676	-1.14750
H	5.08684	3.48425	2.52549
C	3.78993	2.19209	3.67489

```
H            2.39518         0.78682         4.53205
H           -2.13099        -0.03474         4.56991
C           -3.56664         1.12995         3.45412
H           -4.90860         2.12558         2.08361
H           -5.07318        -4.65319         1.17155
C           -4.39199        -4.73079        -0.87476
H           -3.56179        -4.52634        -2.86044
H            6.48613        -0.82912        -3.60334
H            4.09127         2.60904         4.63879
H           -3.89299         1.70757         4.32217
H           -4.92303        -5.65802        -1.10291
```

6.8.8 [P(μ-NTer)]$_2$ (A)

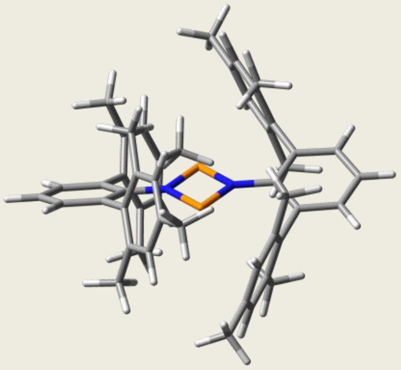

```
102
[P(μ-NTer)]2 @ PBE-D3/def2-TZVP
N            0.00000         0.00000        -1.11385
N           -0.00000         0.00000         1.11385
P            1.34262        -0.00000        -0.00000
P           -1.34262         0.00000        -0.00000
C           -0.00000         0.00000         2.51591
C            1.16113         0.38443         3.23104
C           -1.16113        -0.38443         3.23104
C            1.14008         0.37505         4.63003
C           -1.14008        -0.37505         4.63003
C           -0.00000         0.00000         5.33747
H            2.04398         0.68170         5.16021
H           -2.04398        -0.68170         5.16021
H           -0.00000         0.00000         6.42817
C            0.00000        -0.00000        -2.51591
C            1.16113        -0.38443        -3.23104
C           -1.16113         0.38443        -3.23104
C            1.14008        -0.37505        -4.63003
C           -1.14008         0.37505        -4.63003
C            0.00000        -0.00000        -5.33747
H            2.04398        -0.68170        -5.16021
H           -2.04398         0.68170        -5.16021
H            0.00000        -0.00000        -6.42817
C           -2.40636         0.82689        -2.54215
C           -3.50029        -0.05497        -2.41925
C           -2.50192         2.15062        -2.05422
C           -4.67137         0.40392        -1.80681
C           -3.69055         2.56569        -1.45122
C           -4.78609         1.70550        -1.31388
H           -5.51370        -0.28358        -1.69841
H           -3.75917         3.58786        -1.06848
C            2.40636        -0.82689        -2.54215
C            3.50029         0.05497        -2.41925
C            2.50192        -2.15062        -2.05422
C            4.67137        -0.40392        -1.80681
C            3.69055        -2.56569        -1.45122
C            4.78609        -1.70550        -1.31388
H            5.51370         0.28358        -1.69841
H            3.75917        -3.58786        -1.06848
C           -2.40636        -0.82689         2.54215
C           -3.50029         0.05497         2.41925
C           -2.50192        -2.15062         2.05422
C           -4.67137        -0.40392         1.80681
```

C	-3.69055	-2.56569	1.45122
C	-4.78609	-1.70550	1.31388
H	-5.51370	0.28358	1.69841
H	-3.75917	-3.58786	1.06848
C	2.40636	0.82689	2.54215
C	2.50192	2.15062	2.05422
C	3.50029	-0.05497	2.41925
C	3.69055	2.56569	1.45122
C	4.67137	0.40392	1.80681
C	4.78609	1.70550	1.31388
H	3.75917	3.58786	1.06848
H	5.51370	-0.28358	1.69841
C	-3.40133	1.48059	2.89146
H	-4.32826	2.02784	2.67536
H	-3.20718	1.54300	3.97221
H	-2.57107	2.00078	2.38874
C	-1.32928	-3.08748	2.15296
H	-0.93700	-3.13779	3.17924
H	-1.60766	-4.10002	1.83344
H	-0.50090	-2.74378	1.51210
C	-6.05331	-2.17771	0.65358
H	-5.85277	-2.57684	-0.35248
H	-6.52730	-2.98648	1.23121
H	-6.78210	-1.36176	0.55791
C	3.40133	-1.48059	2.89146
H	4.32826	-2.02784	2.67536
H	3.20718	-1.54300	3.97221
H	2.57107	-2.00078	2.38874
C	1.32928	3.08748	2.15296
H	0.50090	2.74378	1.51210
H	0.93700	3.13779	3.17924
H	1.60766	4.10002	1.83344
C	6.05331	2.17771	0.65358
H	5.85277	2.57684	-0.35248
H	6.52730	2.98648	1.23121
H	6.78210	1.36176	0.55791
C	6.05331	-2.17771	-0.65358
H	5.85277	-2.57684	0.35248
H	6.52730	-2.98648	-1.23121
H	6.78210	-1.36176	-0.55791
C	-6.05331	2.17771	-0.65358
H	-5.85277	2.57684	0.35248
H	-6.52730	2.98648	-1.23121
H	-6.78210	1.36176	-0.55791
C	-1.32928	3.08748	-2.15296
H	-0.93700	3.13779	-3.17924
H	-1.60766	4.10002	-1.83344
H	-0.50090	2.74378	-1.51210
C	1.32928	-3.08748	-2.15296
H	0.93700	-3.13779	-3.17924
H	1.60766	-4.10002	-1.83344
H	0.50090	-2.74378	-1.51210
C	3.40133	1.48059	-2.89146
H	4.32826	2.02784	-2.67536
H	3.20718	1.54300	-3.97221
H	2.57107	2.00078	-2.38874
C	-3.40133	-1.48059	-2.89146
H	-4.32826	-2.02784	-2.67536
H	-3.20718	-1.54300	-3.97221
H	-2.57107	-2.00078	-2.38874

6.8.9 {[PN(Ter)]$_2$(μ-CNDmp)} (B with Y=NDmp)

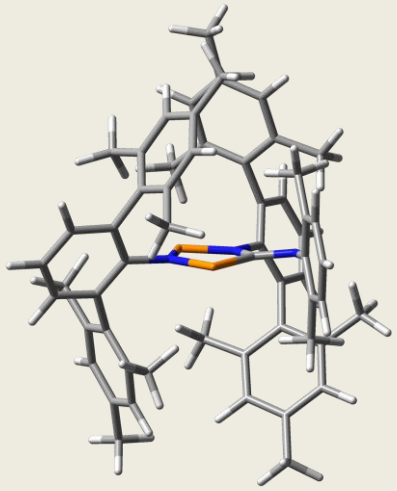

```
121
{[PN(Ter)]₂(μ-CNDmp)} @ PBE-D3/def2-TZVP
C          6.03865       -0.43584       -2.87928
C          4.59101       -0.83650       -2.77369
C          4.21068       -1.94551       -2.00743
C          3.59929       -0.15249       -3.47595
C          6.19087        0.15623        1.19247
C          2.88412       -2.37443       -1.93910
C          4.51308        1.52780       -0.09971
C          2.25250       -0.54262       -3.43227
C          4.95927        1.01782        1.12531
C          2.52429       -3.63806       -1.20676
C          2.91074        2.81763       -1.54141
C          1.89182       -1.64442       -2.63660
C          1.25690        0.18572       -4.29928
C          3.34056        2.27663       -0.20292
C          0.21064       -3.13495       -3.64528
C          3.12153       -2.12636        1.82848
C          4.21515        1.31060        2.26760
C          0.50863       -2.20554       -2.63758
C         -1.00174       -3.81511       -3.66433
C          2.58712        2.53299        0.96448
C          2.43453       -2.07832        4.24254
C          2.06483       -2.14912        2.89494
C          3.03881        2.07083        2.21469
C         -0.46144       -1.94006       -1.64514
C          1.48016       -2.09147        5.25829
C          1.35277        3.36244        0.84376
C          1.44329        4.73784        1.08853
C          0.68931       -2.24088        2.56075
C         -1.93127       -3.57418       -2.65833
C         -1.76981        3.94397       -2.58076
C          0.08842       -1.11590        0.69015
C          2.31187        2.37358        3.49909
C         -1.69086       -2.63443       -1.64515
C          0.13080       -2.17336        4.92310
C          0.34448        5.57537        0.91189
C          0.11146        2.82931        0.42448
C         -0.28570       -2.24261        3.58797
C         -3.48777       -0.30567       -1.87630
C         -0.86154        5.03902        0.47366
C         -1.00659        3.66419        0.22567
C         -1.74905       -2.32123        3.26243
C         -2.74758        3.42393       -1.55966
C         -2.78989       -2.42523       -0.65888
C         -3.66463       -1.33081       -0.79184
C         -2.35540        3.20383       -0.22184
C         -4.07066        3.16132       -1.93152
C         -2.13386       -4.61314        0.45288
C         -3.04636       -3.42526        0.30616
C         -4.75038       -1.21874        0.08664
C         -3.28768        2.71772        0.71903
C         -5.01433        2.68767       -1.01601
C         -2.90421        2.50357        2.15782
C         -4.15541       -3.28393        1.14339
C         -4.59888        2.46799        0.30069
C         -5.00962       -2.17818        1.06559
```

C	-6.42481	2.38213	-1.44107
C	-6.16129	-2.02342	2.02235
H	6.57723	-1.09064	-3.58290
H	6.14644	0.59539	-3.24330
H	6.55093	-0.51479	-1.90973
H	7.01796	0.58837	0.61001
H	4.97432	-2.51139	-1.46635
H	3.87776	0.70412	-4.09657
H	6.53315	0.02313	2.22755
H	5.09133	1.33336	-1.00447
H	5.98767	-0.84425	0.77694
H	3.69454	2.65958	-2.29243
H	1.31294	1.27306	-4.14163
H	2.11045	-4.38394	-1.90338
H	3.40998	-4.07574	-0.72866
H	0.96663	-3.32686	-4.40860
H	4.09816	-1.87872	2.26415
H	2.68342	3.89259	-1.49078
H	1.46940	0.00132	-5.36441
H	3.20361	-3.10607	1.33785
H	2.00061	2.31227	-1.90387
H	1.75651	-3.46175	-0.43730
H	0.22550	-0.13334	-4.10861
H	4.54692	0.92738	3.23661
H	3.49650	-2.00575	4.49045
H	-1.21952	-4.53365	-4.45608
H	2.90225	-1.39023	1.04268
H	2.40340	5.14255	1.41263
H	1.78644	-2.03316	6.30410
H	-1.41491	4.95446	-2.32859
H	-0.87472	3.30544	-2.64161
H	-2.22943	3.98053	-3.57682
H	-2.92262	-0.70358	-2.72928
H	2.20100	1.46183	4.10340
H	0.42968	6.64474	1.11097
H	-2.94973	0.58164	-1.50634
H	-2.88884	-4.09765	-2.65387
H	2.87573	3.10557	4.09973
H	-0.62867	-2.17352	5.70882
H	-2.02465	-3.33144	2.92889
H	1.31098	2.78493	3.32400
H	-4.46026	0.04841	-2.24305
H	-2.35594	-2.07946	4.14550
H	-1.73136	5.68183	0.32729
H	-4.36996	3.33294	-2.96909
H	-1.09814	-4.27671	0.61767
H	-2.13359	-5.24445	-0.44830
H	-2.03161	-1.63927	2.44940
H	-5.42084	-0.36167	-0.01607
H	-2.44254	-5.23762	1.30190
H	-2.17380	1.68484	2.26055
H	-2.43508	3.40197	2.58648
H	-6.53410	1.31835	-1.71163
H	-6.71799	2.97318	-2.31937
H	-4.34848	-4.05645	1.89303
H	-3.78408	2.24900	2.76256
H	-5.32056	2.10235	1.03619
H	-7.14115	2.58455	-0.63257
H	-6.91006	-1.31714	1.63808
H	-5.81362	-1.64103	2.99579

```
H          -6.65975       -2.98479        2.21259
N           0.30571       -2.29789        1.18998
N          -0.22458       -0.88681       -0.67479
N          -0.00261        1.39971        0.25364
P           0.19704        0.40070        1.66210
P          -0.24877        0.71702       -1.25358
```

6.8.10 $C_6H_4P_2N$-Dmp (1Dmp)

```
30
C6H4P2N-Dmp (1Dmp) @ PBE-D3/def2-TZVP
C          -0.00000        0.72015       -2.14193
C          -0.00000        1.41277       -3.38204
C          -0.00000       -0.72015       -2.14193
P          -0.00000        1.49479       -0.56735
C          -0.00000        0.70929       -4.56780
H          -0.00000        2.50480       -3.39082
C          -0.00000       -0.70929       -4.56780
H          -0.00000        1.24634       -5.51746
C          -0.00000       -1.41277       -3.38204
H          -0.00000       -1.24634       -5.51746
H          -0.00000       -2.50480       -3.39082
P          -0.00000       -1.49479       -0.56735
C           0.00000        0.00000        1.71402
C           1.23290       -0.00000        2.39305
C          -1.23290        0.00000        2.39305
N           0.00000        0.00000        0.26973
C           1.20715       -0.00000        3.79291
C           2.53485       -0.00000        1.64237
C           0.00000        0.00000        4.48898
H           2.15339       -0.00000        4.33741
C          -1.20715        0.00000        3.79291
H           0.00000        0.00000        5.58001
H          -2.15339        0.00000        4.33741
C          -2.53485        0.00000        1.64237
H           2.62165       -0.88213        0.99036
H           3.38299       -0.00000        2.33790
H           2.62165        0.88213        0.99036
H          -2.62165        0.88213        0.99036
H          -3.38299        0.00000        2.33790
H          -2.62165       -0.88213        0.99036
```

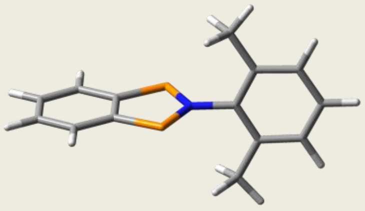

6.8.11 $C_6H_4P_2N$-Ter (1Ter)

```
62
C6H4P2N-Ter (1Ter) @ PBE-D3/def2-TZVP
C          -1.20347       -3.37485        0.03457
C          -1.22857       -1.97365        0.03776
C          -0.00012       -1.27769        0.00000
C           1.22825       -1.97380       -0.03778
C           1.20297       -3.37500       -0.03466
C          -0.00029       -4.07532       -0.00006
H          -2.15385       -3.91029        0.07094
H           2.15329       -3.91056       -0.07107
H          -0.00036       -5.16643       -0.00010
C           2.54347       -1.27203       -0.09598
C           3.02471       -0.77809       -1.32594
```

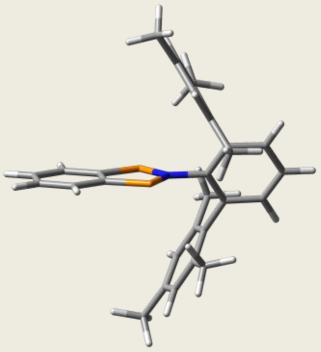

C	3.32415	-1.14814	1.07201
C	4.25949	-0.12193	-1.35397
C	4.55087	-0.47976	0.99774
C	5.03180	0.05325	-0.20166
H	4.62967	0.26002	-2.30949
H	5.14723	-0.37285	1.90804
C	-2.54371	-1.27172	0.09596
C	-3.32437	-1.14775	-1.07202
C	-3.02491	-0.77775	1.32593
C	-4.55102	-0.47923	-0.99776
C	-4.25962	-0.12146	1.35396
C	-5.03190	0.05382	0.20164
H	-5.14737	-0.37227	-1.90806
H	-4.62978	0.26050	2.30947
N	-0.00002	0.16521	0.00003
P	0.67033	1.01511	1.33425
C	-2.24510	-0.96624	2.59981
H	-2.85083	-0.68836	3.47221
H	-1.91749	-2.00924	2.72106
H	-1.33601	-0.34306	2.61099
C	-6.33317	0.80883	0.24912
H	-6.83785	0.67951	1.21675
H	-6.16486	1.88906	0.11082
H	-7.01810	0.47991	-0.54453
C	-2.85791	-1.71859	-2.38499
H	-3.54478	-1.44175	-3.19526
H	-1.85381	-1.35313	-2.64796
H	-2.79617	-2.81683	-2.34990
C	2.85760	-1.71887	2.38499
H	3.54451	-1.44210	3.19526
H	1.85356	-1.35327	2.64794
H	2.79572	-2.81711	2.34993
C	2.24483	-0.96643	-2.59981
H	2.85057	-0.68859	-3.47221
H	1.91710	-2.00938	-2.72109
H	1.33581	-0.34314	-2.61093
C	6.33314	0.80813	-0.24914
H	6.83781	0.67877	-1.21677
H	6.16494	1.88838	-0.11083
H	7.01805	0.47913	0.54450
C	-0.31891	2.58298	-0.64600
C	-0.62504	3.82435	-1.26757
C	0.31949	2.58291	0.64605
C	-0.31315	5.00896	-0.63716
H	-1.10919	3.83249	-2.24660
C	0.62603	3.82420	1.26755
C	0.31453	5.00889	0.63709
H	-0.54980	5.95916	-1.11860
H	1.11016	3.83223	2.24659
H	0.55148	5.95903	1.11848
P	-0.67033	1.01527	-1.33410

6.8.12 $C_6H_4P_2N$-^{tBu}Bhp (1^{tBu}Bhp)

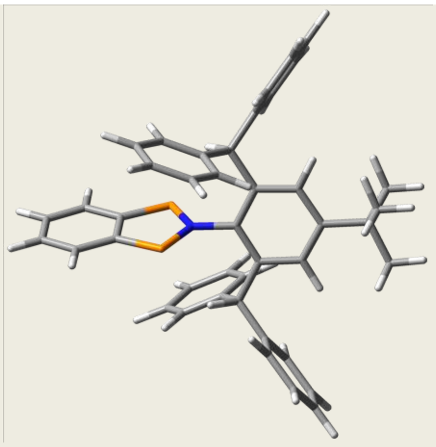

82

$C_6H_4P_2N$-^{tBu}Bhp (1^{tBu}Bhp) @ PBE-D3/def2-TZVP

P	0.25185	2.09012	-1.48738
N	0.07227	1.25418	-0.00030
C	0.27543	3.65946	-0.71180
P	-0.01544	2.09776	1.49072
C	-0.00372	-0.18721	-0.00327
C	0.13318	3.66390	0.72258
C	0.40614	4.89678	-1.39837
C	1.16892	-0.94571	-0.16933
C	-1.25402	-0.81827	0.16246
C	0.13933	4.90475	1.41517
C	0.40424	6.08236	-0.69562
H	0.50794	4.90174	-2.48585
C	1.06936	-2.34244	-0.16552
C	2.52129	-0.25182	-0.31847
C	-1.29956	-2.21246	0.15511
C	-2.52393	0.01551	0.31762
H	0.03910	4.91555	2.50275
C	0.27188	6.08628	0.71813
H	0.50629	7.02950	-1.22781
H	1.98534	-2.91536	-0.29792
C	-0.15228	-3.00035	-0.00575
H	2.37837	0.55688	-1.05576
C	3.58581	-1.16805	-0.90522
C	2.94378	0.43006	0.97298
H	-2.27072	-2.69032	0.28655
H	-2.29573	0.79688	1.06303
C	-3.68009	-0.78901	0.89474
C	-2.86898	0.75072	-0.96737
H	0.27480	7.03634	1.25488
C	-0.27556	-4.52709	0.00057
C	3.54572	-1.44971	-2.27909
C	4.58648	-1.76307	-0.12974
C	3.52923	1.70081	0.92426
C	2.75027	-0.17618	2.22098
C	-3.66636	-1.09598	2.26379
C	-4.74045	-1.26142	0.11432
C	-3.31084	2.07771	-0.90770
C	-2.74436	0.13660	-2.22031
C	1.08018	-5.21998	-0.19126
C	-0.87181	-4.97937	1.34932
C	-1.21363	-4.96266	-1.14381
H	2.76838	-0.98873	-2.89354
C	4.47341	-2.31163	-2.86158
C	5.51771	-2.62897	-0.71041
H	4.64269	-1.54146	0.93696
H	3.66759	2.18730	-0.04412
C	3.90050	2.36029	2.09581
C	3.13247	0.47530	3.39447
H	2.27293	-1.15698	2.27212
H	-2.84224	-0.73132	2.88196
C	-4.67856	-1.86427	2.83639
C	-5.75701	-2.03290	0.68522
H	-4.77532	-1.01804	-0.94851
H	-3.39355	2.56890	0.06465
C	-3.60853	2.78317	-2.07350

```
C          -3.05388        0.83532       -3.38802
H          -2.37831       -0.89051       -2.27986
H           1.78490       -4.96765        0.61452
H           1.54581       -4.94983       -1.15032
H           0.93990       -6.31051       -0.18244
H          -1.86219       -4.53665        1.52462
H          -0.21776       -4.68459        2.18289
H          -0.98099       -6.07431        1.36824
H          -2.21588       -4.52570       -1.03438
H          -1.32100       -6.05787       -1.15089
H          -0.81109       -4.65012       -2.11835
H           4.42693       -2.51556       -3.93283
C           5.46365       -2.90878       -2.07599
H           6.29180       -3.08338       -0.08940
H           4.33419        3.36017        2.04070
C           3.70303        1.74843        3.33593
H           2.96961       -0.00722        4.35979
H          -4.65051       -2.09030        3.90382
C          -5.72901       -2.33970        2.04579
H          -6.57681       -2.39199        0.06045
H          -3.92943        3.82424       -2.00993
C          -3.48098        2.16295       -3.31864
H          -2.94607        0.34534       -4.35729
H           6.19263       -3.58247       -2.52912
H           3.98701        2.26524        4.25403
H          -6.52402       -2.93976        2.49131
H          -3.70743        2.71500       -4.23220
```

6.8.13 $(C_6H_4P_2N\text{-}Ter)_3$ (3Ter)

186

$(C_6H_4P_2N\text{-}Ter)_3$ (3Ter)@ PBE-D3/def2-SVP

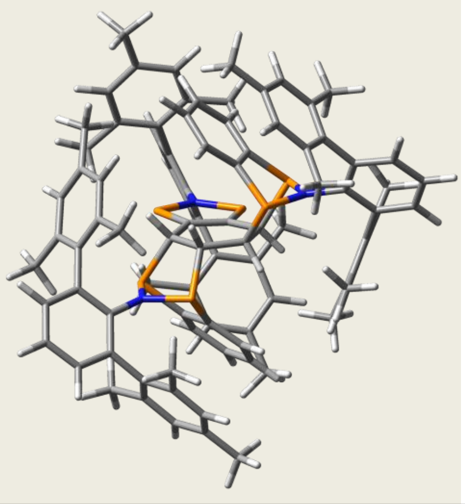

```
P          -0.35450        1.45367        2.58755
N          -0.00000       -0.00000        3.47684
C          -0.15776        0.68806        1.02633
P           0.35450       -1.45367        2.58755
C          -0.00000       -0.00000        4.91348
C           0.15776       -0.68806        1.02633
C          -0.20992        1.50822       -0.23078
C           1.19586       -0.29531        5.62308
C          -1.19586        0.29531        5.62308
C           0.20992       -1.50822       -0.23078
P           1.18713        2.81974       -0.10098
C          -0.08851        0.76929       -1.59194
H          -1.13035        2.12385       -0.24904
C           1.17057       -0.29368        7.03288
C           2.51213       -0.56767        4.96548
C          -1.17057        0.29368        7.03288
C          -2.51213        0.56767        4.96548
P          -1.18713       -2.81974       -0.10098
H           1.13035       -2.12385       -0.24904
C           0.08851       -0.76929       -1.59194
N           1.18376        3.10401       -1.84036
C           2.56666        1.58177       -0.22727
P           1.32039        1.55015       -2.64880
H          -1.00511        1.03371       -2.15930
H           2.10563       -0.51513        7.56972
C          -0.00000       -0.00000        7.74021
```

C	3.00716	-1.89682	4.89674
C	3.32033	0.51698	4.54350
H	-2.10563	0.51513	7.56972
C	-3.00716	1.89682	4.89674
C	-3.32033	-0.51698	4.54350
N	-1.18376	-3.10401	-1.84036
C	-2.56666	-1.58177	-0.22727
P	-1.32039	-1.55015	-2.64880
H	1.00511	-1.03371	-2.15930
C	1.24403	4.37610	-2.45868
C	2.64922	0.96815	-1.50214
C	3.47543	1.23078	0.78019
H	-0.00000	-0.00000	8.84053
C	4.30014	-2.11424	4.39184
C	2.16546	-3.06132	5.35500
C	4.61237	0.25050	4.05228
C	2.82185	1.93704	4.64128
C	-4.30014	2.11424	4.39184
C	-2.16546	3.06132	5.35500
C	-4.61237	-0.25050	4.05228
C	-2.82185	-1.93704	4.64128
C	-1.24403	-4.37610	-2.45868
C	-3.47543	-1.23078	0.78019
C	-2.64922	-0.96815	-1.50214
C	0.18725	5.30240	-2.24482
C	2.35060	4.72841	-3.27730
C	3.64165	0.01718	-1.77129
C	4.48213	0.28811	0.50186
H	3.40744	1.68696	1.77867
H	4.67953	-3.14751	4.33819
C	5.12303	-1.05566	3.96561
H	1.89832	-2.98039	6.42829
H	1.20837	-3.10287	4.79401
H	2.69415	-4.02182	5.20213
H	5.24436	1.09783	3.73882
H	2.45655	2.17100	5.66215
H	3.61741	2.66196	4.38225
H	1.96592	2.11235	3.95434
H	-4.67953	3.14751	4.33819
C	-5.12303	1.05566	3.96561
H	-1.89832	2.98039	6.42829
H	-1.20837	3.10287	4.79401
H	-2.69415	4.02182	5.20213
H	-5.24436	-1.09783	3.73882
H	-2.45655	-2.17100	5.66215
H	-3.61741	-2.66196	4.38225
H	-1.96592	-2.11235	3.95434
C	-2.35060	-4.72841	-3.27730
C	-0.18725	-5.30240	-2.24482
H	-3.40744	-1.68696	1.77867
C	-4.48213	-0.28811	0.50186
C	-3.64165	-0.01718	-1.77129
C	0.24656	6.56547	-2.86164
C	-0.97741	4.89979	-1.40046
C	2.36314	5.99220	-3.89912
C	3.51226	3.79728	-3.40835
H	3.71050	-0.44722	-2.76707
C	4.56734	-0.31066	-0.76458
H	5.20401	0.01998	1.28574
C	6.50444	-1.32739	3.42424

C	-6.50444	1.32739	3.42424
C	-2.36314	-5.99220	-3.89912
C	-3.51226	-3.79728	-3.40835
C	-0.24656	-6.56547	-2.86164
C	0.97741	-4.89979	-1.40046
H	-5.20401	-0.01998	1.28574
C	-4.56734	0.31066	-0.76458
H	-3.71050	0.44722	-2.76707
H	-0.57852	7.27548	-2.69618
C	1.32197	6.90851	-3.69422
C	-1.99159	4.08409	-1.96420
C	-1.05717	5.29980	-0.04197
H	3.22459	6.26171	-4.52965
C	4.45442	3.72293	-2.34707
C	3.68248	3.00186	-4.56834
H	5.36768	-1.03801	-0.97118
H	7.06220	-0.38985	3.23400
H	7.10264	-1.94379	4.12645
H	6.45576	-1.89224	2.46874
H	-7.06220	0.38985	3.23400
H	-7.10264	1.94379	4.12645
H	-6.45576	1.89224	2.46874
H	-3.22459	-6.26171	-4.52965
C	-1.32197	-6.90851	-3.69422
C	-3.68248	-3.00186	-4.56834
C	-4.45442	-3.72293	-2.34707
H	0.57852	-7.27548	-2.69618
C	1.05717	-5.29980	-0.04197
C	1.99159	-4.08409	-1.96420
H	-5.36768	1.03801	-0.97118
H	1.35185	7.89572	-4.17944
C	-3.06035	3.66583	-1.15178
C	-1.91736	3.66163	-3.40978
C	-2.13072	4.83589	0.74124
C	0.00000	6.19078	0.56026
C	5.53385	2.83258	-2.45587
C	4.30384	4.59349	-1.12588
C	4.77745	2.11787	-4.63084
C	2.69532	3.07800	-5.70590
H	-1.35185	-7.89572	-4.17944
C	-4.77745	-2.11787	-4.63084
C	-2.69532	-3.07800	-5.70590
C	-5.53385	-2.83258	-2.45587
C	-4.30384	-4.59349	-1.12588
C	2.13072	-4.83589	0.74124
C	-0.00000	-6.19078	0.56026
C	3.06035	-3.66583	-1.15178
C	1.91736	-3.66163	-3.40978
H	-3.84634	3.03432	-1.59299
C	-3.14073	4.01383	0.20964
H	-1.86883	4.53771	-4.08837
H	-0.99914	3.06990	-3.60996
H	-2.79141	3.04525	-3.69574
H	-2.17631	5.12588	1.80365
H	1.00465	5.72770	0.47152
H	0.05346	7.16905	0.04065
H	-0.19660	6.37657	1.63350
H	6.25418	2.76882	-1.62422
C	5.70988	2.01214	-3.58541
H	4.23631	5.66657	-1.39831

H	3.37255	4.35593	-0.56912
H	5.15245	4.45783	-0.42867
H	4.90077	1.49024	-5.52872
H	1.68204	2.77778	-5.36485
H	2.60407	4.10855	-6.10418
H	2.98804	2.40852	-6.53745
H	-4.90077	-1.49024	-5.52872
C	-5.70988	-2.01214	-3.58541
H	-2.60407	-4.10855	-6.10418
H	-2.98804	-2.40852	-6.53745
H	-1.68204	-2.77778	-5.36485
H	-6.25418	-2.76882	-1.62422
H	-4.23631	-5.66657	-1.39831
H	-3.37255	-4.35593	-0.56912
H	-5.15245	-4.45783	-0.42867
H	2.17631	-5.12588	1.80365
C	3.14073	-4.01383	0.20964
H	-1.00465	-5.72770	0.47152
H	-0.05346	-7.16905	0.04065
H	0.19660	-6.37657	1.63350
H	3.84634	-3.03432	-1.59299
H	1.86883	-4.53771	-4.08837
H	0.99914	-3.06990	-3.60996
H	2.79141	-3.04525	-3.69574
C	-4.24865	3.48675	1.08352
C	6.85696	1.03582	-3.64976
C	-6.85696	-1.03582	-3.64976
C	4.24865	-3.48675	1.08352
H	-5.16940	3.28767	0.50043
H	-3.94834	2.53208	1.56582
H	-4.49816	4.19562	1.89754
H	7.82903	1.53584	-3.45926
H	6.74723	0.24707	-2.87486
H	6.91692	0.53575	-4.63591
H	-6.91692	-0.53575	-4.63591
H	-7.82903	-1.53584	-3.45926
H	-6.74723	-0.24707	-2.87486
H	5.16940	-3.28767	0.50043
H	3.94834	-2.53208	1.56582
H	4.49816	-4.19562	1.89754

186

($(C_6H_4P_2N\text{-}Ter)_3$ (3Ter)@ PBE-D3/def2-TZVP

P	-0.00763	1.48016	2.60022
N	0.00000	0.00000	3.47991
C	0.00211	0.70211	1.04609
P	0.00763	-1.48016	2.60022
C	0.00000	0.00000	4.91758
C	-0.00211	-0.70211	1.04609
C	0.11272	1.51602	-0.20702
C	1.10677	-0.52578	5.62122
C	-1.10677	0.52578	5.62122
C	-0.11272	-1.51602	-0.20702
P	1.73145	2.51325	-0.10415
C	0.07130	0.76937	-1.56697
H	-0.66325	2.29422	-0.22153
C	1.08147	-0.52126	7.02336

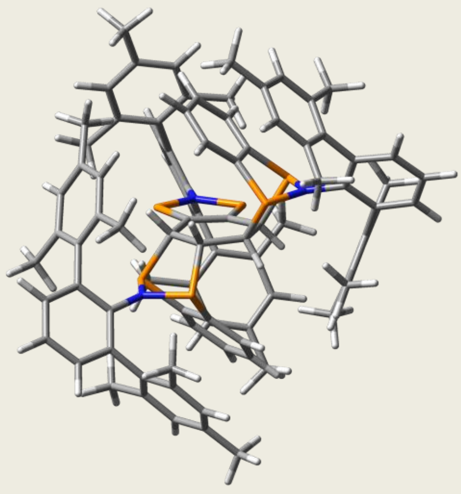

C	2.33825	-1.05911	4.96681
C	-1.08147	0.52126	7.02336
C	-2.33825	1.05911	4.96681
P	-1.73145	-2.51325	-0.10415
H	0.66325	-2.29422	-0.22153
C	-0.07130	-0.76937	-1.56697
N	1.77249	2.78619	-1.82555
C	2.82719	1.03334	-0.21994
P	1.59309	1.24833	-2.61508
H	-0.77117	1.21071	-2.12206
H	1.94477	-0.92429	7.55609
C	0.00000	0.00000	7.72684
C	2.54472	-2.45358	4.88553
C	3.35343	-0.16936	4.56354
H	-1.94477	0.92429	7.55609
C	-2.54472	2.45358	4.88553
C	-3.35343	0.16936	4.56354
N	-1.77249	-2.78619	-1.82555
C	-2.82719	-1.03334	-0.21994
P	-1.59309	-1.24833	-2.61508
H	0.77117	-1.21071	-2.12206
C	2.08593	4.01870	-2.44956
C	2.77576	0.41199	-1.48513
C	3.64040	0.50742	0.78280
H	0.00000	0.00000	8.81787
C	3.75784	-2.92966	4.38076
C	1.48816	-3.42519	5.33888
C	4.55678	-0.69202	4.07418
C	3.17417	1.32065	4.68123
C	-3.75784	2.92966	4.38076
C	-1.48816	3.42519	5.33888
C	-4.55678	0.69202	4.07418
C	-3.17417	-1.32065	4.68123
C	-2.08593	-4.01870	-2.44956
C	-3.64040	-0.50742	0.78280
C	-2.77576	-0.41199	-1.48513
C	1.23722	5.12946	-2.24264
C	3.23403	4.14178	-3.26343
C	3.54328	-0.72067	-1.74846
C	4.42341	-0.62004	0.50958
H	3.66920	0.96644	1.77218
H	3.91082	-4.01026	4.31504
C	4.78028	-2.06698	3.97163
H	1.26574	-3.31314	6.41050
H	0.54292	-3.26307	4.79874
H	1.80982	-4.45985	5.16181
H	5.34769	0.00189	3.77514
H	2.85555	1.60883	5.69403
H	4.10940	1.84758	4.45027
H	2.39843	1.68872	3.99005
H	-3.91082	4.01026	4.31504
C	-4.78028	2.06698	3.97163
H	-1.26574	3.31314	6.41050
H	-0.54292	3.26307	4.79874
H	-1.80982	4.45985	5.16181
H	-5.34769	-0.00189	3.77514
H	-2.85555	-1.60883	5.69403
H	-4.10940	-1.84758	4.45027
H	-2.39843	-1.68872	3.99005
C	-3.23403	-4.14178	-3.26343

C	-1.23722	-5.12946	-2.24264
H	-3.66920	-0.96644	1.77218
C	-4.42341	0.62004	0.50958
C	-3.54328	0.72067	-1.74846
C	1.54571	6.34826	-2.85726
C	0.01109	4.98393	-1.40811
C	3.49421	5.36930	-3.88668
C	4.19357	3.00936	-3.40377
H	3.51025	-1.19196	-2.73289
C	4.37971	-1.22576	-0.74737
H	5.06954	-1.02598	1.28786
C	6.07891	-2.61212	3.43983
C	-6.07891	2.61212	3.43983
C	-3.49421	-5.36930	-3.88668
C	-4.19357	-3.00936	-3.40377
C	-1.54571	-6.34826	-2.85726
C	-0.01109	-4.98393	-1.40811
H	-5.06954	1.02598	1.28786
C	-4.37971	1.22576	-0.74737
H	-3.51025	1.19196	-2.73289
H	0.88315	7.20099	-2.69573
C	2.66146	6.46934	-3.68556
C	-1.14308	4.40344	-1.97442
C	-0.00000	5.41344	-0.06491
H	4.38400	5.45978	-4.51309
C	5.11554	2.76167	-2.36211
C	4.20327	2.20622	-4.56112
H	5.00544	-2.09711	-0.94952
H	6.79246	-1.80641	3.22026
H	6.55161	-3.29505	4.16170
H	5.92005	-3.18706	2.51371
H	-6.79246	1.80641	3.22026
H	-6.55161	3.29505	4.16170
H	-5.92005	3.18706	2.51371
H	-4.38400	-5.45978	-4.51309
C	-2.66146	-6.46934	-3.68556
C	-4.20327	-2.20622	-4.56112
C	-5.11554	-2.76167	-2.36211
H	-0.88315	-7.20099	-2.69573
C	0.00000	-5.41344	-0.06491
C	1.14308	-4.40344	-1.97442
H	-5.00544	2.09711	-0.94952
H	2.88490	7.42158	-4.16905
C	-2.28257	4.24293	-1.17975
C	-1.15513	3.96305	-3.41370
C	-1.15437	5.21202	0.70111
C	1.20704	6.07194	0.54759
C	6.00748	1.69454	-2.48441
C	5.15438	3.64121	-1.14209
C	5.11077	1.14159	-4.63814
C	3.24554	2.46630	-5.69280
H	-2.88490	-7.42158	-4.16905
C	-5.11077	-1.14159	-4.63814
C	-3.24554	-2.46630	-5.69280
C	-6.00748	-1.69454	-2.48441
C	-5.15438	-3.64121	-1.14209
C	1.15437	-5.21202	0.70111
C	-1.20704	-6.07194	0.54759
C	2.28257	-4.24293	-1.17975
C	1.15513	-3.96305	-3.41370

H	-3.17494	3.79564	-1.62305
C	-2.30450	4.62341	0.16661
H	-0.91334	4.79591	-4.09085
H	-0.40134	3.18258	-3.60439
H	-2.13841	3.56262	-3.69363
H	-1.15130	5.52303	1.74941
H	2.10211	5.44279	0.43272
H	1.42769	7.03603	0.06512
H	1.05090	6.25099	1.61931
H	6.70931	1.49892	-1.66909
C	6.01321	0.86266	-3.60912
H	5.30886	4.69627	-1.41403
H	4.20847	3.59905	-0.57930
H	5.96005	3.33244	-0.46363
H	5.10615	0.51043	-5.53126
H	2.20280	2.38843	-5.34793
H	3.37120	3.47693	-6.10810
H	3.39183	1.74000	-6.50296
H	-5.10615	-0.51043	-5.53126
C	-6.01321	-0.86266	-3.60912
H	-3.37120	-3.47693	-6.10810
H	-3.39183	-1.74000	-6.50296
H	-2.20280	-2.38843	-5.34793
H	-6.70931	-1.49892	-1.66909
H	-5.30886	-4.69627	-1.41403
H	-4.20847	-3.59905	-0.57930
H	-5.96005	-3.33244	-0.46363
H	1.15130	-5.52303	1.74941
C	2.30450	-4.62341	0.16661
H	-2.10211	-5.44279	0.43272
H	-1.42769	-7.03603	0.06512
H	-1.05090	-6.25099	1.61931
H	3.17494	-3.79564	-1.62305
H	0.91334	-4.79591	-4.09085
H	0.40134	-3.18258	-3.60439
H	2.13841	-3.56262	-3.69363
C	-3.51511	4.37613	1.02362
C	6.95042	-0.31193	-3.68884
C	-6.95042	0.31193	-3.68884
C	3.51511	-4.37613	1.02362
H	-4.43740	4.38328	0.42603
H	-3.44667	3.39513	1.51978
H	-3.60846	5.13481	1.81322
H	7.98341	-0.02031	-3.44794
H	6.66034	-1.09226	-2.96672
H	6.94655	-0.76237	-4.69050
H	-6.94655	0.76237	-4.69050
H	-7.98341	0.02031	-3.44794
H	-6.66034	1.09226	-2.96672
H	4.43740	-4.38328	0.42603
H	3.44667	-3.39513	1.51978
H	3.60846	-5.13481	1.81322

6.8.14 Benzene

```
12
benzene (C6H6) @ PBE0-D3/def2-TZVP
C          0.00000        1.38777        0.00000
C          1.20185        0.69389        0.00000
C          1.20185       -0.69389        0.00000
C          0.00000       -1.38777        0.00000
C         -1.20185       -0.69389        0.00000
C         -1.20185        0.69389        0.00000
H          0.00000        2.47201        0.00000
H          2.14082        1.23601        0.00000
H          2.14082       -1.23601        0.00000
H          0.00000       -2.47201        0.00000
H         -2.14082       -1.23601        0.00000
H         -2.14082        1.23601        0.00000
```

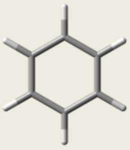

6.8.15 Naphthalene

```
18
naphthalene @ PBE0-D3/def2-TZVP
C          0.00000        2.41702        0.70431
C          0.00000        1.23625        1.39379
C          0.00000        0.00000        0.71108
C          0.00000        0.00000       -0.71108
C          0.00000        1.23625       -1.39379
C          0.00000        2.41702       -0.70431
H          0.00000       -1.23231        2.47877
H          0.00000        3.35933        1.23997
H          0.00000        1.23231        2.47877
C          0.00000       -1.23625        1.39379
C          0.00000       -1.23625       -1.39379
H          0.00000        1.23231       -2.47877
H          0.00000        3.35933       -1.23997
C          0.00000       -2.41702       -0.70431
C          0.00000       -2.41702        0.70431
H          0.00000       -1.23231       -2.47877
H          0.00000       -3.35933       -1.23997
H          0.00000       -3.35933        1.23997
```

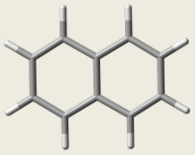

6.8.16 Indole

```
16
indole @ PBE0-D3/def2-TZVP
C          0.49212        0.61489        0.00000
C          0.00000       -0.71128        0.00000
C         -1.36054       -1.00005        0.00000
C         -2.23730        0.06625        0.00000
C         -1.77276        1.38927        0.00000
C         -0.42270        1.67193        0.00000
C          1.91904        0.52683        0.00000
C          2.23570       -0.79895        0.00000
H         -1.72196       -2.02240        0.00000
H         -3.30453       -0.12224        0.00000
H         -2.49107        2.20081        0.00000
H         -0.07422        2.69870        0.00000
```

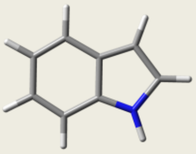

```
H          2.62228        1.34441        0.00000
H          1.04690       -2.54823        0.00000
H          3.20157       -1.27966        0.00000
N          1.08567       -1.54640        0.00000
```

6.8.17 borazine

```
12
borazine @ PBE0-D3/def2-TZVP
N          0.00000        1.40388        0.00000
B         -1.25149        0.72255        0.00000
N         -1.21580       -0.70194        0.00000
B          0.00000       -1.44510        0.00000
N          1.21580       -0.70194        0.00000
B          1.25149        0.72255        0.00000
H          0.00000        2.40997        0.00000
H         -2.28824        1.32112        0.00000
H         -2.08710       -1.20499        0.00000
H          0.00000       -2.64223        0.00000
H          2.08710       -1.20499        0.00000
H          2.28824        1.32112        0.00000
```

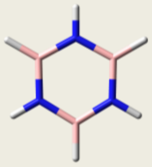

6.8.18 [P(μ-NH)]$_2$ (AH)

```
6
[P(μ-NH)]2 @ PBE-D3/def2-TZVP
H          0.00000        0.00000        2.10922
H          0.00000        0.00000       -2.10922
N          0.00000        0.00000        1.09588
N          0.00000        0.00000       -1.09588
P          0.00000        1.31759        0.00000
P          0.00000       -1.31759        0.00000
```

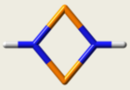

6.8.19 {[PN(H)]$_2$(μ-CNH)} (BH)

```
9
{[PN(H)]₂(μ-CNH)} @ PBE-D3/def2-TZVP
C          0.00000        1.15494        0.00000
N         -0.15681        2.43609        0.00000
N         -1.16095        0.33791        0.00000
N          0.55212       -1.34026        0.00000
P          1.49344        0.10694        0.00000
P         -1.11841       -1.34031        0.00000
H          1.03317       -2.23862        0.00000
H          0.74460        2.92175        0.00000
H         -2.04363        0.85162        0.00000
```

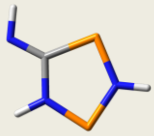

6.8.20 $C_6H_4P_2N$-H (1H)

```
14
C6H4P2NH @ PBE-D3/def2-TZVP
C          0.00000        0.72062       -0.18048
C          0.00000       -0.72062       -0.18048
C          0.00000       -1.41243       -1.42104
```

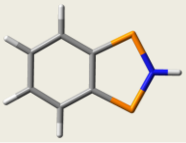

```
C          0.00000       -0.70924       -2.60669
C          0.00000        0.70924       -2.60669
C          0.00000        1.41243       -1.42104
H          0.00000       -2.50443       -1.43007
H          0.00000       -1.24651       -3.55619
H          0.00000        1.24651       -3.55619
H          0.00000        2.50443       -1.43007
H          0.00000        0.00000        3.22178
P          0.00000        1.49967        1.39430
P          0.00000       -1.49967        1.39430
N          0.00000        0.00000        2.20290
```

7 References

[1] C. B. Fischer, S. Xu, H. Zipse, *Chem. Eur. J.* **2006**, *12*, 5779–5784.

[2] P. Wucher, J. B. Schwaderer, S. Mecking, *ACS Catal.* **2014**, *4*, 2672–2679.

[3] F. Reiß, A. Schulz, A. Villinger, N. Weding, F. Reiss, A. Schulz, A. Villinger, N. Weding, *Dalton Trans.* **2010**, *39*, 9962–9972.

[4] J. Bresien, C. Hering-Junghans, A. Schulz, M. Thomas, A. Villinger, *Organometallics* **2018**, *37*, 2571–2580.

[5] P. H. M. Budzelaar, *gNMR for Windows*, IvorySoft, **2006**.

[6] G. M. Sheldrick, *Acta Crystallogr., Sect. A: Found. Adv.* **2015**, *71*, 3–8.

[7] G. M. Sheldrick, *Acta Crystallogr., Sect. C: Struct. Chem.* **2015**, *71*, 3–8.

[8] G. M. Sheldrick, *SADABS Version 2*, University of Göttingen, Germany, **2004**.

[9] J. Bresien, J. M. Goicoechea, A. Hinz, M. T. Scharnhölz, A. Schulz, T. Suhrbier, A. Villinger, *Dalton Trans.* **2019**, *48*, 3786–3794.

[10] B. Zhao, X. Peng, Z. Wang, C. Xia, K. Ding, *Chem. Eur. J.* **2008**, *14*, 7847–7857.

[11] L. S. Chen, G. J. Chen, C. Tamborski, *J. Organomet. Chem.* **1980**, *193*, 283–292.

[12] *Gaussian 09, Revision E.01*, M. J. Frisch, G. W. Trucks, H. B. Schlegel, G. E. Scuseria, M. A. Robb, J. R. Cheeseman, G. Scalmani, V. Barone, B. Mennucci, G. A. Petersson, H. Nakatsuji, M. Caricato, X. Li, H. P. Hratchian, A. F. Izmaylov, J. Bloino, G. Zheng, J. L. Sonnenberg, M. Hada, M. Ehara, K. Toyota, R. Fukuda, J. Hasegawa, M. Ishida, T. Nakajima, Y. Honda, O. Kitao, H. Nakai, T. Vreven, J. A. Montgomery Jr., J. E. Peralta, F. Ogliaro, M. Bearpark, J. J. Heyd, E. Brothers, K. N. Kudin, V. N. Staroverov, T. Keith, R. Kobayashi, J. Normand, K. Raghavachari, A. Rendell, J. C. Burant, S. S. Iyengar, J. Tomasi, M. Cossi, N. Rega, J. M. Millam, M. Klene, J. E. Knox, J. B. Cross, V. Bakken, C. Adamo, J. Jaramillo, R. Gomperts, R. E. Stratmann, O. Yazyev, A. J. Austin, R. Cammi, C. Pomelli, J. W. Ochterski, R. L. Martin, K. Morokuma, V. G. Zakrzewski, G. A. Voth, P. Salvador, J. J. Dannenberg, S. Dapprich, A. D. Daniels, O. Farkas, J. B. Foresman, J. V. Ortiz, J. Cioslowski, D. J. Fox, Gaussian, Inc., Wallingford CT, **2013**.

[13] F. Neese, *Wiley Interdiscip. Rev.: Comput. Mol. Sci.* **2018**, *8*, e1327.

[14] J. P. Perdew, K. Burke, M. Ernzerhof, *Phys. Rev. Lett.* **1996**, *77*, 3865–3868.

[15] J. P. Perdew, K. Burke, M. Ernzerhof, *Phys. Rev. Lett.* **1997**, *78*, 1396–1396.

[16] C. Adamo, V. Barone, *J. Chem. Phys.* **1999**, *110*, 6158–6170.

[17] S. Grimme, J. Antony, S. Ehrlich, H. Krieg, *J. Chem. Phys.* **2010**, *132*, 154104.

[18] S. Grimme, S. Ehrlich, L. Goerigk, *J. Comput. Chem.* **2011**, *32*, 1456–1465.

[19] F. Weigend, R. Ahlrichs, *Phys. Chem. Chem. Phys.* **2005**, *7*, 3297–305.

[20] D. Hegarty, M. A. Robb, *Mol. Phys.* **1979**, *38*, 1795–1812.

[21] R. H. A. Eade, M. A. Robb, *Chem. Phys. Lett.* **1981**, *83*, 362–368.

[22] H. B. Schlegel, M. A. Robb, *Chem. Phys. Lett.* **1982**, *93*, 43–46.

[23] P. E. M. Siegbahn, *Chem. Phys. Lett.* **1984**, *109*, 417–423.

[24] F. Bernardi, A. Bottoni, J. J. W. McDouall, M. A. Robb, H. B. Schlegel, *Faraday Symp. Chem. Soc.* **1984**, *19*, 137.

[25] M. A. Robb, U. Niazi, *Rep. Mol. Theory* **1990**, *1*, 23–55.

[26] M. Frisch, I. N. Ragazos, M. A. Robb, H. Bernhard Schlegel, *Chem. Phys. Lett.* **1992**, *189*, 524–528.

[27] N. Yamamoto, T. Vreven, M. A. Robb, M. J. Frisch, H. Bernhard Schlegel, *Chem. Phys. Lett.* **1996**, *250*, 373–378.

[28] M. Klene, M. A. Robb, M. J. Frisch, P. Celani, *J. Chem. Phys.* **2000**, *113*, 5653–5665.

[29] C. J. Cramer, *Essentials of Computational Chemistry, Theories and Models*, John Wiley & Sons, Ltd, Chichester, UK, **2004**.

[30] J. Bresien, T. Kröger-Badge, S. Lochbrunner, D. Michalik, H. Müller, A. Schulz, E. Zander, *Chem. Sci.* **2019**, *10*, 3486–3493.

[31] F. London, *J. Phys. Radium* **1937**, *8*, 397–409.

[32] R. McWeeny, *Phys. Rev.* **1962**, *126*, 1028–1034.

[33] R. Ditchfield, *Mol. Phys.* **1974**, *27*, 789–807.

[34] K. Wolinski, J. F. Hinton, P. Pulay, *J. Am. Chem. Soc.* **1990**, *112*, 8251.

[35] J. R. Cheeseman, G. W. Trucks, T. a Keith, M. J. Frisch, *J. Chem. Phys.* **1996**, *104*, 5497–5509.

[36] C. J. Jameson, A. De Dios, A. Keith Jameson, *Chem. Phys. Lett.* **1990**, *167*, 575–582.

[37] C. van Wüllen, *Phys. Chem. Chem. Phys.* **2000**, *2*, 2137–2144.

[38] L. Falivene, Z. Cao, A. Petta, L. Serra, A. Poater, R. Oliva, V. Scarano, L. Cavallo, *Nat. Chem.* **2019**, *11*, 872–879.

[39] A. Schulz, *Z. Anorg. Allg. Chem.* **2014**, *640*, 2183–2192.

[40] E. Miliordos, K. Ruedenberg, S. S. Xantheas, *Angew. Chem., Int. Ed.* **2013**, *52*, 5736–5739.

[41] K. Yamaguchi, *Chem. Phys. Lett.* **1975**, *33*, 330–335.

[42] M. Nakano, R. Kishi, T. Nitta, T. Kubo, K. Nakasuji, K. Kamada, K. Ohta, B. Champagne, E. Botek, K. Yamaguchi, *J. Phys. Chem. A* **2005**, *109*, 885–891.

[43] H. Fliegl, S. Taubert, O. Lehtonen, D. Sundholm, *Phys. Chem. Chem. Phys.* **2011**, *13*, 20500.

[44] D. Sundholm, H. Fliegl, R. J. F. Berger, *Wiley Interdiscip. Rev.: Comput. Mol. Sci.* **2016**, *6*, 639–678.

[45] J. Jusélius, D. Sundholm, J. Gauss, *J. Chem. Phys.* **2004**, *121*, 3952–3963.

[46] S. Taubert, D. Sundholm, J. Jusélius, *J. Chem. Phys.* **2011**, *134*, 054123.

[47] M. Rauhalahti, S. Taubert, D. Sundholm, V. Liégeois, *Phys. Chem. Chem. Phys.* **2017**, *19*, 7124–7131.

[48] J. Ahrens, B. Geveci, C. Law, *ParaView: An End-User Tool for Large Data Visualization*, Visualization Handbook, Elsevier, **2005**.

[49] Z. Chen, C. S. Wannere, C. Corminboeuf, R. Puchta, P. von Ragué Schleyer, *Chem. Rev.* **2005**, *105*, 3842–3888.

[50] P. von R. Schleyer, C. Maerker, A. Dransfeld, H. Jiao, N. J. R. van E. Hommes, *J. Am. Chem. Soc.* **1996**, *118*, 6317–6318.

[51] H. Fallah-Bagher-Shaidaei, C. S. Wannere, C. Corminboeuf, R. Puchta, P. v. R. Schleyer, *Org. Lett.* **2006**, *8*, 863–866.

[52] D. Sundholm, R. J. F. Berger, H. Fliegl, *Phys. Chem. Chem. Phys.* **2016**, *18*, 15934–15942.

[53] S. G. Patra, N. Mandal, *Int. J. Quantum Chem.* **2020**, *120*, 1–21.

[54] T. Beweries, R. Kuzora, U. Rosenthal, A. Schulz, A. Villinger, *Angew. Chem., Int. Ed.* **2011**, *50*, 8974–8978.
